# A systematic review of mathematical models of mosquito-borne pathogen transmission: 1970–2010

**DOI:** 10.1098/rsif.2012.0921

**Published:** 2013-04-06

**Authors:** Robert C. Reiner, T. Alex Perkins, Christopher M. Barker, Tianchan Niu, Luis Fernando Chaves, Alicia M. Ellis, Dylan B. George, Arnaud Le Menach, Juliet R. C. Pulliam, Donal Bisanzio, Caroline Buckee, Christinah Chiyaka, Derek A. T. Cummings, Andres J. Garcia, Michelle L. Gatton, Peter W. Gething, David M. Hartley, Geoffrey Johnston, Eili Y. Klein, Edwin Michael, Steven W. Lindsay, Alun L. Lloyd, David M. Pigott, William K. Reisen, Nick Ruktanonchai, Brajendra K. Singh, Andrew J. Tatem, Uriel Kitron, Simon I. Hay, Thomas W. Scott, David L. Smith

**Affiliations:** 1 Fogarty International Center, National Institutes of Health, Bethesda, MD, USA; 2 Department of Entomology, School of Veterinary Medicine, University of California, Davis, CA, USA; 3 Center for Vectorborne Diseases, School of Veterinary Medicine, University of California, Davis, CA, USA; 4 Department of Pathology, Microbiology, and Immunology, School of Veterinary Medicine, University of California, Davis, CA, USA; 5 Division of Integrated Biodefense, Georgetown University Medical Center, Washington DC, USA; 6 Department of Microbiology and Immunology, Georgetown University Medical Center, Washington DC, USA; 7 Graduate School of Environmental Sciences and Global Center of Excellence Program on Integrated Field Environmental Science, Hokkaido University, Sapporo, Japan; 8 Programa de Investigación en Enfermedades Tropicales, Escuela de Medicina Veterinaria, Universidad Nacional, Heredia, Costa Rica; 9 Institute of Tropical Medicine (NEKKEN) and Global Center of Excellence Program on Tropical and Emergent Infectious Diseases, Nagasaki University, Nagasaki, Japan; 10 The Rubenstein School of Environment and Natural Resources, University of Vermont, Burlington, VT, USA; 11 Department of Defense, Fort Detrick, MD, USA; 12 Center for Disease Dynamics, Economics and Policy, Washington, DC, USA; 13 Emerging Pathogens Institute, University of Florida, Gainesville, FL, USA; 14 Department of Biology, University of Florida, Gainesville, FL, USA; 15 Department of Geography, University of Florida, Gainesville, FL, USA; 16 Department of Environmental Studies, Emory University, Atlanta, GA, USA; 17 Center for Communicable Disease Dynamics, Department of Epidemiology, Harvard School of Public Health, Boston, MA, USA; 18 Department of Epidemiology, Johns Hopkins Bloomberg School of Public Health, Baltimore, MD, USA; 19 Malaria Research Institute, Johns Hopkins Bloomberg School of Public Health, Baltimore, MD, USA; 20 Malaria Drug Resistance and Chemotherapy Laboratory, Queensland Institute of Medical Research, Herston, Queensland, Australia; 21 Spatial Ecology and Epidemiology Group, Department of Zoology, Oxford University, Oxford, UK; 22 School of International and Public Affairs, Columbia University, New York, NY, USA; 23 Department of Microbiology and Immunology, Columbia University College of Physicians and Surgeons, New York, NY, USA; 24 Department of Ecology and Evolutionary Biology, Princeton University, Princeton, NJ, USA; 25 Center for Advanced Modeling, Department of Emergency Medicine, Johns Hopkins University, Baltimore, MD, USA; 26 Department of Biological Sciences, Eck Institute for Global Health, University of Notre Dame, Notre Dame, IN, USA; 27 Department of Infectious Disease Epidemiology, Imperial College, London, UK; 28 Department of Disease Control, London School of Hygiene and Tropical Medicine, London, UK; 29 School of Biological and Biomedical Sciences, Durham University, Durham, UK; 30 Department of Mathematics and Biomathematics Graduate Program, North Carolina State University, Raleigh, NC, USA; 31 Department of Geography and Environment, University of Southampton, Highfield, Southampton, UK

**Keywords:** infectious disease dynamics, vector-borne disease, epidemiology, dengue, West Nile, filariasis

## Abstract

Mathematical models of mosquito-borne pathogen transmission originated in the early twentieth century to provide insights into how to most effectively combat malaria. The foundations of the Ross–Macdonald theory were established by 1970. Since then, there has been a growing interest in reducing the public health burden of mosquito-borne pathogens and an expanding use of models to guide their control. To assess how theory has changed to confront evolving public health challenges, we compiled a bibliography of 325 publications from 1970 through 2010 that included at least one mathematical model of mosquito-borne pathogen transmission and then used a 79-part questionnaire to classify each of 388 associated models according to its biological assumptions. As a composite measure to interpret the multidimensional results of our survey, we assigned a numerical value to each model that measured its similarity to 15 core assumptions of the Ross–Macdonald model. Although the analysis illustrated a growing acknowledgement of geographical, ecological and epidemiological complexities in modelling transmission, most models during the past 40 years closely resemble the Ross–Macdonald model. Modern theory would benefit from an expansion around the concepts of heterogeneous mosquito biting, poorly mixed mosquito-host encounters, spatial heterogeneity and temporal variation in the transmission process.

## 1. Introduction

More than a century has passed since Ross first described malaria transmission mathematically [1,2] and more than 50 years since Macdonald updated and extended Ross's theory and applied it to the Global Malaria Eradication Programme (GMEP, 1955–1969) [3–6]. The origin of the Ross–Macdonald theory was a pair of malaria models that Ross published in 1908 and 1911 [1,2]. After the Second World War, Macdonald picked up where Ross left off and focused on developing a highly applied theory to complement the global public health rollout of DDT, the creation of the World Health Organization and burgeoning enthusiasm for malaria eradication. The state of mathematical theory was solidified in the 1950s by Macdonald [5,7] and in the 1960s by Garrett-Jones [8,9]. The Macdonald era effectively came to a close with Macdonald's death in 1967, posthumous publication of his last paper in 1968 [10], and the end of the GMEP in 1969. By that point, Ross's vision had been fulfilled by the development of a fully quantitative theory, consisting of a set of linked concepts, notation and metrics for understanding and measuring mosquito-borne pathogen transmission and control. A detailed account of the development of the theory up to this point in history was recently published [11].

Since the conclusion of the GMEP, the theory of mosquito-borne pathogen transmission has expanded around popular themes from that era. Along the way, it has also been shaped by contemporary public health challenges. These include a renewed interest in malaria eradication [12], an expanding global dengue epidemic [13,14], the enormous global health burden of filariasis [15–18], outbreaks of chikungunya virus around the Indian Ocean [19], epidemics of Rift Valley Fever and concerns about its potential range expansion [20] and the epidemic invasion of West Nile virus into the New World [14,21]. These diverse challenges have resulted in models developed by many authors working on many different pathogens, with different constraints on measuring transmission, different mosquitoes, different immune responses and with different tools and public health concerns.

Advancing the theory of mosquito-borne pathogen transmission into the future first requires an assessment of developments that have been made since the time of Macdonald. To answer the challenge of describing and summarizing recent advances, we developed a bibliography of mechanistic models of mosquito-borne pathogen transmission and a system for classifying the full range of biological assumptions that these models have made. In taking such a quantitative and analytical approach, we were able to identify patterns in the literature not readily assessed in a standard review article. The fruits of this effort are summaries of the literature at several levels of detail: (i) a database that contains the results of classifying 388 models; (ii) a set of 85 tables and figures that summarize the results of this classification by pathogen and over time; and (iii) a numerical score for each model between 0 and 15, called the *RM* index, that describes in how many ways each model has relaxed core assumptions of the Ross–Macdonald model. Because complexity is not an unconditionally beneficial property of a model, this index should not be interpreted as a measure of quality but as an indication of dissimilarity from the Ross–Macdonald model. On the whole, though, the collection of these scores and their components across all models allows for identification of areas of research interest within the field since 1970. This inventory of models then culminates with a discussion of how the field has marshalled around a few major themes yet has neglected several topics that have been broached but that have received insufficient attention.

## 2. Methods

Our intent in this study was to identify and review as many publications as possible that had the following properties: (i) it was about a mosquito-borne pathogen, (ii) it included some sort of equation, and (iii) the underlying model was mechanistic in its approach to the study of transmission. We consider mechanistic models to be those in which the equations, formulae or computer simulations are based on assumptions about the processes or proximate causal mechanisms under consideration. These stand in contrast to purely descriptive or statistical models that seek to fit data without consideration of underlying biological mechanisms.

### 2.1. Bibliographic compilation

To identify a set of publications that embodied these properties, seven authors (C.M.B., T.N., L.F.C., A.M.E., D.B.G., A.L.M., J.R.C.P.) first conducted a literature search of the Science Citation Index Expanded covering the years 1900–2009. Publications returned by this literature search consisted of any article or proceedings paper with both of the following in its title, keyword list or abstract: (i) the name of any of several common mosquito-borne pathogens or pathogen-bearing mosquito species and (ii) at least one of a list of words related to mathematical, simulation or statistical modelling. This search identified a total of 2026 publications, of which 182 were subsequently determined to have used mechanistic models.

Although we were willing to accept the inevitability that our bibliography might never be truly complete, the fact that our initial search resulted in a bibliography of only 182 publications was a concern. We therefore expanded on this initial bibliography by several ad hoc methods, including examination of our own personal bibliographies, perusal of the literature cited by the initial 182 publications, Internet database searches using those authors' last names to find other publications and personal contact with some of those authors. After expanding the bibliography by these methods and making the decision to review models published before 1970 separately [11], the bibliography included 523 publications. To finalize the bibliography, six authors (R.C.R., T.A.P., C.M.B., T.N., T.W.S., D.L.S.) read the remaining publications and excluded from further review any publications that clearly did not fit the criteria described earlier as well as other papers that were not about transmission. Some of the publications excluded on this premise focused instead on infection within a single host, mosquito population dynamics or mosquito population genetics, whereas others were purely descriptive models that lacked a mechanistic underpinning. This resulted in a final bibliography of 325 publications, which is available in the electronic supplementary material, S1.

The collection of models that we then analysed, however, was somewhat larger than this collection of publications because there is not a one-to-one correspondence between models and publications. In some cases, two or more models were presented, for the first time, within a single publication. In other cases, multiple publications presented the same analysis on the same models. Consequently, our final collection of models was the result of lumping and splitting the content of publications such that each model–analysis pair was included only once. If the same model was analysed in two different ways in two different publications, then these publications were analysed separately. Multiple models from a single publication were more often split than a single model from multiple publications was lumped, resulting in a final collection of 388 models (see the electronic supplementary material, table S1).

### 2.2. Model classification

To evaluate each model in a standardized way, 26 reviewers (R.C.R., T.A.P., C.M.B., T.N., L.F.C., A.L.M., J.R.C.P., D.B., C.B., C.C., D.A.T.C., A.J.G., M.L.G., P.W.G., D.M.H., G.J., E.Y.K., E.M., S.W.L., A.L.L., D.M.P., W.K.R., N.R., B.K.S., A.J.T., D.L.S.) used a 79-part questionnaire (see the electronic supplementary material, S2) to evaluate the richness of biological details incorporated by models. To compensate for differences between reviewers, each model was scored twice by two different reviewers. For some models, the two resulting scores for the same model were considerably different from each other. In these cases, two reviewers (R.C.R. and T.A.P.) went through those scores individually and either deemed one of the scores preferable, or, if neither was adequate, then the model was scored a third time. Each scoring thus constitutes a consensus view from multiple evaluations of a model by multiple people. The questionnaire and the final database resulting from this process are available in the electronic supplementary material, S2 and S3, respectively.

In designing the questionnaire, our goal was to encompass the full spectrum of biological details that have been included in mechanistic models of mosquito-borne pathogen transmission in the past 40 years (table 1). Three major sections of the questionnaire focus on the three essential components common to all of these models: a host, a mosquito and encounters between them. The host section comprised five questions about population dynamics and nine questions about infection dynamics. The mosquito section was divided into four questions about aquatic ecology, six questions about adult ecology, and seven questions about infection dynamics. The section about encounters between hosts and mosquitoes consisted of five questions about topics such as heterogeneous biting (i.e. mosquitoes bite some hosts more than others) and mixing between hosts and mosquitoes. The questionnaire also contained sections about spatial dynamics (three questions), control (two questions) and approaches to analysing models (one question). A small number of models made simplifying assumptions about transmission such that mosquitoes were not included in the model. The structure of the questionnaire diverted evaluators of these models to special sections, depending on whether the model was structured similarly to one for directly transmitted pathogens (two questions) or whether the model assumed that mosquito dynamics were ‘fast’ and effectively equilibrated on the relatively ‘slow’ time-scale of infection dynamics in vertebrate hosts (three questions). The logic structure of the questionnaire also meant that not every question was answered for every model. Consequently, results about some sections of the questionnaire were limited to a subset of models included in the inventory.

**Table 1.** Overview of the questionnaire used for model classification.

section	topic	questions
spatial dynamics	spatial configuration	33, 34
	which species moves	35
aquatic mosquito ecology	adult emergence	36
	larval population dynamics	37, 38
	differences across space	39
quasi-direct transmission	how it was implemented	40, 41
minimal mosquito assumption	how it was implemented	42–44
adult mosquito ecology	demography	45, 46
	blood feeding	47, 48
	differences across space	49
	other	50
mosquito infection dynamics	host infection states	51, 52
	pathogen latency	53, 54
	other	55
	differences among types	56, 57
host population dynamics	host attributes	58
	population dynamics	59–61
	differences across space	62
host infection dynamics	host infection states	63–65
	waning immunity	66
	clinical outcomes	67
	superinfection	68, 69
	differences among types	70, 71
mixing and biting	biting distribution on hosts	72, 73
	assumptions about mixing	74
	transmission efficiencies	75, 76
control	types considered	77
	aspects analysed	78
analysis	types performed	79

### 2.3. Ross–Macdonald dissimilarity index

Although there is value in the complex and nuanced dataset that resulted from classifying models according to the questionnaire, we also wanted to quantitatively assess in a straightforward way the extent to which models of the past 40 years adhered to or departed from Ross–Macdonald assumptions. To accomplish this, we identified a set of 15 core questions from the questionnaire that embodied consensus features of the Ross–Macdonald model (table 2). We then evaluated whether each model agreed with the consensus Ross–Macdonald assumption corresponding to each of the 15 questions on the questionnaire (table 2). If a model's assumptions matched those of the Ross–Macdonald model or made even simpler assumptions, the model's Ross–Macdonald dissimilarity index (or *RM* index for short) remained unchanged. If, for a particular question, the model expanded on the Ross–Macdonald assumption, then the *RM* index for that model was augmented by 1. For example, if a model explicitly incorporated a pathogen latency period in the mosquito (question 53), then its *RM* index was augmented by 1 because the Ross–Macdonald model incorporated this feature only implicitly. This procedure was repeated for each of the 15 questions in table 2. Thus, values of the *RM* index range from 0 (identical to or simpler than Ross–Macdonald) to 15 (more complicated than Ross–Macdonald in every way we measured).

**Table 2.** Questions and responses used to create *RM* index.

questions	Ross–Macdonald assumption	refinement (*RM*+1)
question 25. Which one of the following best describes the way aquatic populations were modelled?	implicitly	explicitly
question 28. How many spatial locations were included in or implied by the model?	one place with no immigration or emigration	there was more than one location or place; or the model included terms describing immigration
question 29. How many mosquito taxa, genotypes or phenotypes were considered?	one	more than one
question 30. How many pathogen taxa, genotypes or phenotypes were considered?	one	more than one
question 31. How many vertebrate taxa, genotypes or phenotypes were considered?	one	more than one
question 46. What assumptions were made about adult mosquito mortality in the absence of control?	constant *per capita* mortality	any further refinement
question 47. What assumptions were made about mosquito blood feeding rates in the absence of control?	blood feeding occurred at a constant *per capita* rate	any further refinement
question 48. What assumption was made about the proportion of blood meals taken on the pathogen's host(s)?	feeding on other vertebrate hosts was included only implicitly or not at all	any further refinement (excluding one only based on there being multiple host species)
question 53. Did the model consider pathogen latency in mosquitoes?	implicitly	explicitly
question 66. Was it possible for immunity to wane?	no	yes
question 68. Was it possible for a vertebrate host to be ‘superinfected’ or ‘co-infected?’	no	yes
question 72. How were blood meals distributed among vertebrate hosts?	homogenously	heterogeneously
question 74. Which one of the following describes mixing?	well-mixed	not well-mixed
question 75. Which of the following parameters or terms describe transmission from the infectious mosquito to its vertebrate host?	set to constant	differed based on some aspect of system
question 76. Which of the following parameters or terms describes transmission from the infectious host to the mosquito?	set to constant	differed based on some aspect of system

Analysis of the *RM*-index data involved examining the distribution of *RM*-index values across all models, across models of each pathogen, and by which core assumptions contributed the most to *RM*-index values. We also examined which core assumptions tended to be relaxed together in the same model to determine which combination of assumptions may be over- or underrepresented in the literature. Finally, we calculated an evenness index of *RM*-index contributions from different core assumptions to determine the extent to which refinements of the Ross–Macdonald model have been isolated to a limited subset of core assumptions. The evenness index we used [22] is related to a type of entropy measure and varies between 0 (only one assumption contributes to *RM* index) and 1 (even distribution).

## 3. Results

Evaluating 388 models according to a 79-part questionnaire produced a wealth of information. To satisfy the curiosities of readers who wish to examine this information at various levels of detail, we present the results in three ways. First, the greatest amount of detail is contained in the full database that resulted from evaluation of the questionnaire for each model (see the electronic supplementary material, S3). Second, a more accessible but still comprehensive presentation of the data is available in a collection of 85 tables in the electronic supplementary material, S4. Third, we highlight only the most striking results below, in the figures, and with the *RM*-index analysis. It is important to note that for ease of interpretation, we often report the number of elaborations on the Ross–Macdonald model relative to the fraction of models to which they are pertinent; as such, for many of the proportions presented, the denominator equals the fraction of pertinent models, not the total number of models considered. For example, out of 388 total models, 139 consider control, and thus results concerning control present the per cent of models out of those 139 that investigate a particular control strategy.

### 3.1. Pathogen

Consistent with the long history of malaria as an object of mathematical modelling and its heavy burden on public health worldwide, it is not surprising that over half of the models we reviewed concerned malaria (59%, 230/388; electronic supplementary material, table S15). Dengue was the second most frequently modelled pathogen (20%, 77/388; electronic supplementary material, table S15), and West Nile virus was third (8%, 31/388; electronic supplementary material, table S15). Several other pathogens have been modelled less frequently, including filariasis and viruses associated with Rift Valley fever, yellow fever, chikungunya, Ross River fever, Japanese encephalitis, Murray Valley encephalitis and western equine encephalitis.

**Figure 1.**
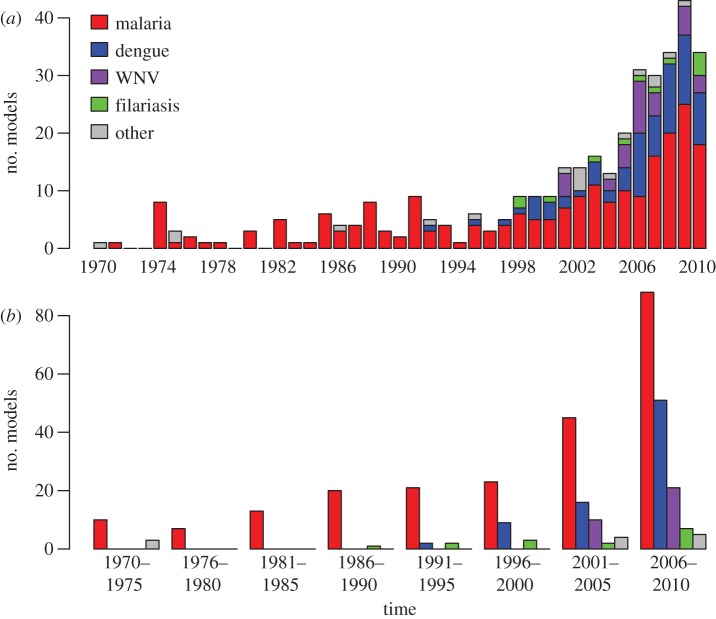
Temporal trend in the publication of models included in the bibliography, grouped by pathogen and binned by (*a*) year and (*b*) 5-year period.

With few exceptions, all of the models in our inventory before the 1990s concerned malaria (figure 1). Models of other pathogens started appearing somewhat more frequently in the 1990s (25%, 14/56), and by the 2000s, models of other pathogens comprised almost half of all models in our inventory from that time period (49%, 133/272). Of particular note, models of West Nile virus only began appearing after its spread across North America in 1999, and an increase in the publication of models of dengue has accompanied the growth of the worldwide epidemic of dengue in recent years. On the whole, the publication of models of all mosquito-borne pathogens has been increasing over the past 40 years (figure 1), with 53 per cent published between 2005 and 2010 (207/388; figure 1).

### 3.2. Host

**Figure 2.**
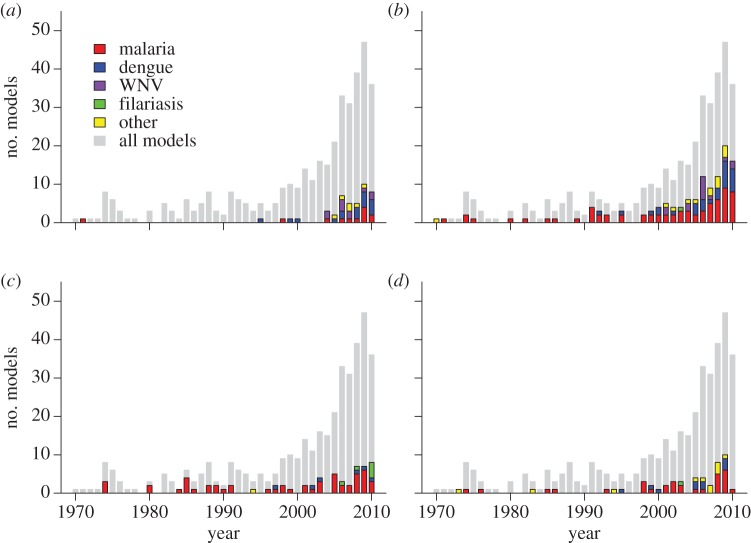
Themes and trends. (*a*) Number by pathogen (bars) and relative per cent (points) of models that explicitly modelled aquatic mosquito populations by year (question 25). (*b*) Number by pathogen and relative per cent of models that modelled pathogen latency in mosquitoes by year (question 53). (*c*) Number by pathogen and relative per cent of models that incorporated potential co-infections or superinfection by year (question 68). (*d*) Number by pathogen and relative per cent of models that used a simulation-based approach by year (questions 51 and 63).

Owing to the short time scales usually considered by models of mosquito-borne pathogen transmission, it is not surprising that many models did not incorporate host population dynamics (37%, 119/318, electronic supplementary material, table S57). Many more, however, explicitly modelled host infection dynamics with at least one state of infection (e.g. exposed or infectious classes; 85%, 318/373; electronic supplementary material, table S19) but not clinical outcomes of infection (e.g. mild or severe symptoms; 24%, 75/318; electronic supplementary material, table S65). The most common complications to a basic model of host infection were the inclusion of waning immunity (29%, 92/318; electronic supplementary material, table S64), simultaneous infection with multiple pathogens (20%, 65/318; electronic supplementary material, table S67), and differences in infection dynamics based on host age (18%, 57/318; electronic supplementary material, table S55). As with many of the results, waning immunity and simultaneous infection with multiple pathogens appear to be commonly examined complexities in large part because of their relevance to the issues of drug resistance and superinfection in malaria, which is the most commonly modelled disease. Proportionally fewer models have allowed for multiple pathogen types or strains in recent years, however, and a larger share of them have been applied to filariasis and dengue (figure 2*c*).

Additional details about hosts were often included when multiple host species or types were modelled. The most common differences between multiple host species or types were the intensity of infection and infectiousness (38%, 15/39), their attractiveness to mosquitoes (46%, 18/39) and the duration of the infectious period (38%, 15/39; electronic supplementary material, table S69). Heterogeneities among different host species and across space were modelled simultaneously only once [23].

### 3.3. Mosquito

**Figure 3.**
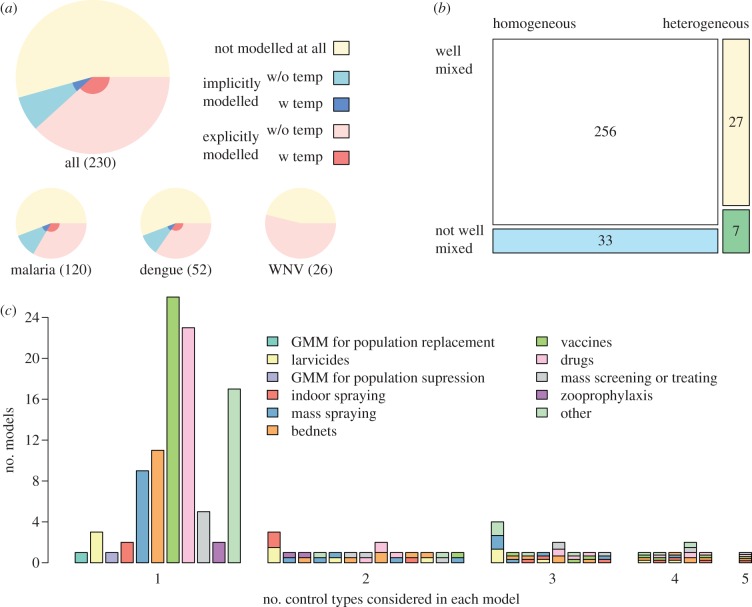
Selected results. (*a*) Assumptions about pathogen latency in mosquitoes, grouped by pathogen and in total (numbers of each in parentheses). The range of assumptions includes: pathogen latency was not modelled at all (yellow); it was either implicitly or explicitly modelled (blue, red); and it was modelled with or without temperature dependence (dark, light). (*b*) Assumptions about the mixing of mosquito–host encounters (well-mixed or not well-mixed, top versus bottom rectangles) and the distribution of blood meals on hosts (homogeneous or heterogeneous, left versus right rectangles). The area of each square corresponds to the proportion of models that make each combination of assumptions, and colour denotes difference from the Ross–Macdonald model. (*c*) Number of models that included individual control measures and combinations thereof. Each bar represents a unique combination of control measures included in at least one model. Bars are grouped according to how many control measures appeared in a single model, and multicoloured bars indicate which control measures comprised each combination.

Consistent with the fact that mosquitoes are, by definition, essential to the transmission of mosquito-borne pathogens, most models explicitly modelled mosquito populations with at least one state variable (62%, 231/373; electronic supplementary material, table S18). A majority of models with explicit mosquito dynamics implemented various aspects of mosquito biology with constant or constant *per capita* rates, including death (82%, 190/231; electronic supplementary material, table S38) and blood feeding (74%, 172/231). The aquatic phase of the mosquito life cycle was not often included explicitly in models (12%, 45/373; electronic supplementary material, table S17), although more models have accounted for it in recent years (figure 2*a*). Density dependence in the aquatic phase was included even less often (62%, 28/45; electronic supplementary material, table S29). Pathogen latency in mosquitoes was ignored completely in 38 per cent of models of mosquito infection dynamics (88/230; electronic supplementary material, table S48; figure 3*a*), and its dependence on temperature was treated in only 16 per cent of those that considered it at all (23/142; electronic supplementary material, table S49; figure 3*a*). Of those models that both implicitly included the aquatic phase of the mosquito and explicitly included mosquitoes, over half of these models assumed that mosquito density was constant (61%, 72/119, electronic supplementary material, table S37), whereas only 14 per cent varied it either sinusoidally or based on a pattern derived from data (17/119; electronic supplementary material, table S37). Moreover, the proportion of models including some form of pathogen latency in mosquitoes has been relatively consistent over time (figure 2*b*).

### 3.4. Mosquito-host encounters

Given the opportunity for a mosquito to take a blood meal on any one of several individual hosts, nearly all models (82%, 303/369; electronic supplementary material, table S72) assumed that blood meal had an equal probability of taking place on any of the available hosts (i.e. homogeneous distribution of blood meals; figure 3*b*). Depending on the extent to which mosquito and host populations are well-mixed, a given mosquito may only have the opportunity to take a blood meal on a limited subset of hosts in the population. The most common assumption (78%, 291/373; electronic supplementary material, table S75) was that contacts were well mixed, i.e. there was an equal probability of any given mosquito encountering any given host (figure 3*b*). This assumption is especially unrealistic at large spatial scales, yet only 17 per cent of models included two or more spatial locations (64/371; electronic supplementary material, table S20). Inclusion of space in a model usually made it necessary to also model movement patterns of mosquitoes or hosts. Host movement was modelled in 69 per cent (44/64) of those models, mosquito movement in 59 per cent (38/64) and both in 38 per cent (24/64; electronic supplementary material, table S27). Only 17 papers estimated parameters relevant to spatial dynamics (host search or selection by mosquitoes, mosquito movement or host movement; electronic supplementary material, table S5), as the remainder of models either lacked relevant data or were framed generally and not around any particular place.

### 3.5. Transmission

Models without explicit representation of mosquitoes either assumed that exposure to pathogens was determined by a quantity such as vectorial capacity (23%, 85/373; electronic supplementary material, table S18) or borrowed directly from theory and models used for directly transmitted pathogens (9%, 32/373; electronic supplementary material, table S18). When mosquito infection dynamics were explicitly modelled, a very common simplifying assumption was that the mosquito-to-host and host-to-mosquito transmission probabilities were constant parameters (87%, 325/373, electronic supplementary material, table S76, and 82%, 304/373, electronic supplementary material, table S78, respectively) rather than depending on pathogen load or other factors. Analysing transmission metrics was a very common technique, and almost half of the models in our inventory either estimated transmission with data or provided data that could be used to do so (39%, 152/388; electronic supplementary material, table S8). The most frequently estimated transmission metrics were *R*_0_ or some other reproductive number (22%, 85/388), the force of infection (16%, 63/388), the prevalence of infection in vertebrate hosts (19%, 75/388), vectorial capacity (15%, 60/388) and the entomological inoculation rate (14%, 54/388; electronic supplementary material, table S7). Temporally varying transmission dynamics at short or long time scales received minimal attention, and the implications of seasonal variation were not studied commonly (13%, 52/388; electronic supplementary material, table S84).

### 3.6. Control

Although the field of mosquito-borne pathogen transmission is inherently an applied one, only 37 per cent of models included a control measure (139/373; electronic supplementary material, table S24). The remainder appear to be less overtly applied and to focus more narrowly on basic understanding of transmission dynamics. Control was considered even less often in models of dengue (25%, 19/76) and West Nile (32%, 10/31) compared with malaria (47%, 102/219) and filariasis (46%, 6/13; electronic supplementary material, table S24). It is important to note that some models that explicitly consider control of mosquito-borne pathogens were excluded from our inventory on the premise that they did not explicitly link to a transmission model. This was likely the case for a number of models of genetically modified mosquitoes and larval control.

Drugs were the most commonly modelled type of control for malaria and likewise overall (26%, 36/139; electronic supplementary material, table S80; figure 3*c*). Adult mosquito spraying (23%, 32/139), larvicides (11%, 15/139) and bednets (20%, 28/139) were also commonly modelled (see the electronic supplementary material, table S80; figure 3*c*). Even though vaccines are not yet available for most mosquito-borne diseases, they were commonly modelled as a precursor to anticipated vaccine development and distribution (24%, 34/139, electronic supplementary material, table S80; figure 3*c*). Most models of control considered only one type (73%, 102/139, electronic supplementary material, table S81; figure 3*c*), but 28 unique combinations of up to five controls have been modelled at least once (figure 3*c*). In particular, entomological controls (i.e. spraying adults, larvicides) are commonly considered in combination with other controls (see the electronic supplementary material, table S80; figure 3*c*). Efficacy is typically the only aspect of control analysed (74%, 103/139), with financial or operational constraints considered far less often (9%, 13/139; electronic supplementary material, table S82).

### 3.7. Ross–Macdonald dissimilarity index

**Figure 4.**
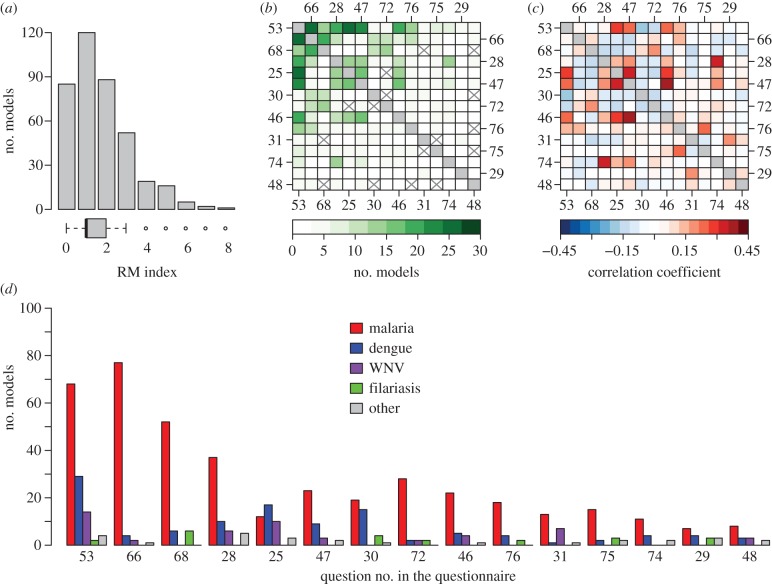
Analysis of RM-index values, which quantify in how many ways models differ from core assumptions of the Ross–Macdonald theory and range from 0 to 15. (*a*) Distribution of RM-index values across all models. (*b*) Number of models in which a particular pair of core assumptions differed from Ross–Macdonald simultaneously. (*c*) Correlation coefficients for each pair of core assumptions. Pairs with positive correlations frequently appeared together or were frequently omitted together, whereas pairs with negative correlations tended not to appear together in the same models. (*d*) Number of models, grouped by pathogen, in which each of the 15 core assumptions differed from those of the Ross–Macdonald model. Core assumptions are specified by the questions from the questionnaire presented in table 2.

The largest *RM* index that we observed was eight out of a maximum of 15, with 53 per cent of models having a score of 0 or 1 and 76 per cent having a score of 2 or less (figure 4*a*). Across all pathogens, the most common refinements to the Ross–Macdonald framework were modelling pathogen latency in mosquitoes (32%, 125/388, question 53), waning immunity (22%, 86/388, question 66), simultaneous infection with multiple pathogens (17%, 65/388, question 68) and multiple spatial locations (16%, 64/388, question 28). The least common refinements were assuming that mosquito-host encounters are not well-mixed (5%, 21/388, question 74), modelling multiple mosquito species or types (4%, 15/388, question 29) and blood feeding on non-host species (3%, 12/388, question 48). All other refinements were modelled between 23 and 45 times, which highlights the predominance of waning immunity, multiple pathogen types and, especially, pathogen latency in mosquitoes as commonly explored model refinements. The inclusion of such details has been facilitated more in recent years by the increasing adoption of simulation-based approaches to modelling transmission (figure 2*d*).

Certain pairs of core assumptions were commonly relaxed together (figure 4*b*). For example, when models explicitly incorporated pathogen latency (question 53), they also tended to explicitly model aquatic mosquito populations (question 25) or to allow for waning immunity (question 66). Co-occurrence of some pairs was due in part to the fact that they were common in general (e.g. questions 53 and 66). Co-occurrence of other pairs appeared to reflect a common underlying biological theme, such as mosquito ecology (e.g. questions 25, 46, 47 and 53; figure 4*b*). In contrast to the commonness of pairs of assumptions, a different comparison is made when one looks at which pairs of core assumptions are highly correlated (either in their joint presence or joint absence; figure 4*c*). For example, the most negatively correlated pair was explicit modelling of aquatic mosquito populations (question 25) and allowance for superinfection in vertebrate hosts (Question 68; *r* =−0.14), which clearly pertain to different modelling themes (e.g. mosquito ecology, host infection dynamics).

Both the magnitude and composition of the *RM* index differed somewhat for models of different pathogens (figure 4*d*). Although the median *RM* index for all pathogens was 1, the maximum—which reflects the variance—was greater for malaria (8) than for all other pathogens (7). The composition of the *RM* index also differed by pathogen. Explicitly modelling aquatic populations (question 25) was one of the most common refinements of models of dengue (22%, 17/77) and West Nile (32%, 10/31), yet it was among the least common refinements for models of malaria (5%, 12/230) and was never included in models of filariasis (0%, 0/16; figure 4*d*). This pattern could be partially attributable to the notion that modelling aquatic dynamics enhances the realism of fine-scale temporal dynamics of mosquito populations. In that case, these details would be of greatest importance for pathogens whose hosts remain infectious for relatively short periods of time during which that fine temporal variation matters most (i.e. dengue, West Nile). Whether models considered multiple types of pathogens, mosquitoes or hosts also varied by pathogen. In accordance with their biology, modelling multiple pathogen strains (question 30) has been relatively common in models of dengue (19%, 15/77) and modelling multiple host species (Question 31) has been common in models of West Nile (23%, 7/31; figure 4*d*).

Calculation of the evenness index showed that, in general, models of malaria addressed the full range of core Ross–Macdonald assumptions with relatively equal effort (evenness = 0.9), whereas models of dengue (0.86), West Nile (0.73) and filariasis (0.69) tended to focus effort on pathogen-specific subsets of core assumptions (figure 4*d*). Models of other pathogens as a group devoted relatively equal attention to the full range of core Ross–Macdonald assumptions (0.93), which reflects the fact that models of the several pathogens comprising that group tended to each focus on limited, but complementary subsets of core assumptions. The breadth of core assumptions addressed by models of malaria, in contrast, is likely due to the historical and numerical prominence of those models.

## 4. Discussion

Over the past 40 years, mathematical models have expanded on the simple but elegant themes introduced by the Ross–Macdonald model. The theory now includes a rich set of models describing immunity, complex infection dynamics, seasonality, stochasticity, superinfection, pathogen evolution, mosquito aquatic ecology, hydrology, heterogeneous biting, host and mosquito behaviour, spatial dynamics, clinical disease, and multiple host and mosquito species. Despite these numerous expansions to the theory, our literature review found that most models published in the past 40 years have adopted most of the same simplifying assumptions used by Ross and Macdonald. Although models typically differ from the Ross–Macdonald model in at least one way, few differ in more than three ways, and most of the attention has been focused on relatively few modelling themes. Some of the assumptions that have been questioned least are those of homogeneous mosquito biting, well-mixed encounters between mosquitoes and hosts, and temporal constancy (figure 3*b*).

### 4.1. Modelling themes

The elaboration on the Ross–Macdonald model that has taken place over the last 40 years can be summarized in several overarching themes. One major theme is the role of temperature in driving patterns of transmission. It was recognized early in the study of mosquito-borne pathogens that transmission is often highly seasonal, which first appeared in the 1970s in models with seasonally forced mosquito densities [24]. Since then, models have incorporated temperature dependence into specific components of the life cycle of mosquitoes and pathogens within them, including pathogen latency in mosquitoes (figure 3*a*), larval development rates, blood feeding rates and adult survival ([25–27] and others thereafter). Much of this relatively recent interest in temperature dependence was prompted by rising concern about climate change and the potential expansion of the geographical range of malaria and dengue [27,28]. The impact of rising temperatures on the geographical range of malaria remains difficult to assess, however, given competing forces such as vector control and economic development that have contracted its range [29].

Perhaps one of the reasons why seasonality has been so difficult to model is that the distribution of mosquitoes is, like most species distributions, affected by multiple interacting factors. There has been a need to develop better models of mosquito population dynamics, including the ecology of immature mosquitoes in their aquatic habitats, but such models remain uncommon. The most common convention has been to consider emergence of adults from aquatic habitats as a parameter, perhaps with seasonal forcing, but to ignore the dynamics of mosquitoes in their aquatic habitat. Some models have recently attempted to incorporate larval ecology, but this has usually been done in large computer simulation models, such as CIMSiM [25,30], HYDREMATS [23,31] and a malaria model by Depinay *et al.* [32]. In fact, models of this kind have been used increasingly with the growing power and availability of computing resources (figure 2*d*).

These details of mosquito ecology often require elaboration on the spatial landscape on which these ecological processes unfold. Research on this topic has roots as far back as 1905, when Ross published a model of random movement of adult mosquitoes and the geographical extent of a zone required to eliminate malaria from an area with larval source management [33]. Otherwise, explicit recognition of space was not integrated into transmission models until the early 1970s, when partial differential equation models with diffusive movement of vectors and hosts were used. Since Bailey's summary of this approach [34], this class of models has largely been neglected. A more recent development has been the use of patch-based metapopulation models, such as [35], with increasingly well-developed notions of mosquito and human movement. In addition to variety in how spatial units are defined, there has also been enormous variety in the scales at which spatial patterns are investigated, ranging from a single population [36] to the entire planet [29]. This diversity of spatial models clearly reflects the diversity of purposes for which they have been constructed.

Perhaps the most common innovations on the Ross–Macdonald model have been more ‘realistic’ models of pathogen infection in the host. In malaria, these began with models of superinfection [37,38], and extended next to consider partial immunity [37,39], realistic infections [40] and then complicated within-host models [41]. Compartment models have been developed for arboviral diseases [34,42], and in the case of dengue, these have been expanded to consider strain interactions, including antibody-dependent enhancement [43] and temporary cross-immunity [44,45]. In filariasis, models with multiple infections have been called ‘macroparasite models’, which count the number of worms per host. A question that has been discussed for decades in models of filariasis is the possibility of a backwards bifurcation because of host–parasite interactions [46–48]. More recently, this phenomenon has also been proposed for malaria [49,50] and dengue [51].

### 4.2. Modelling deficiencies

Although progress has been made in marshalling efforts around a few important themes, there is still work to be carried out on these and other topics. Based on their underrepresentation in the inventory and the importance that empirical studies assign to them, we propose that the following themes deserve more attention hereafter: (i) variation in individual host attributes and their consequences for heterogeneous biting [52,53], (ii) poorly mixed mosquito-host encounters [54], and (iii) spatial heterogeneity as well as temporal variation [55,56].

Host heterogeneity and its consequences for transmission have been addressed with models before. The first paper to do so in the context of mosquito-borne pathogens was Hairston & de Meillon's [57] discussion of the efficiency versus intensity of filariasis transmission when biting is highly unequal. These ideas stem from work on sexually and other directly transmitted pathogens and have been applied in a number of important works on mosquito-borne pathogens since [58–61]. Heterogeneities among hosts have also been incorporated into models with multiple host species [62,63], which have been published increasingly since the invasion of West Nile virus to North America. Especially for pathogens with an enzootic cycle, variation among host species may be amplified by spatial variation in vertebrate host species' densities [64]. Nonetheless, our inventory suggests that the examination of host heterogeneities has been limited mostly to these few pioneering works, despite their demonstrated impact on fundamental concepts, such as the basic reproductive number and the efficacy of control measures [60,61,65,66]. Certainly, there is much potential to push these ideas further and especially to apply them in specific contexts and to connect them with data. Modern empirical techniques, such as blood meal analysis and analyses of pathogen ancestral relationships, could stimulate breakthroughs in this area.

Not only has heterogeneity in the preferences of mosquitoes for certain hosts been largely ignored, but so too has spatial heterogeneity in mosquito density and factors that underlie it (but see Le Menach *et al*. [67]). Mosquito aggregation at locations that oft-bitten hosts frequent accentuates heterogeneous transmission further than what accounting for their individual attributes would suggest. The impact of all of these heterogeneities in a model of transmission dynamics, however, hinges on the model's assumptions about mixing. Some 78 per cent of models we inventoried assumed that encounters between hosts and mosquitoes were well-mixed, rendering the impact of these heterogeneities moot. Well-mixed models effectively average over these heterogeneities, when, in reality, heterogeneities are present at very fine spatial scales [53] and transmission dynamics tend to behave differently at different scales [68,69]. Some models [23,31] have incorporated details of fine-scale spatial heterogeneity in mosquito density by, for instance, linking remotely sensed abiotic data to mosquito density in specific geographical areas of interest. These efforts are a good start, but an unequivocal and empirically supported demonstration of the unique importance of multiple types of heterogeneity impacting the dynamics of mosquito-borne pathogen transmission will require a concerted effort addressing multiple types of heterogeneity. Progress in this area will ultimately require that models address several important complexities simultaneously, rather than in a piecemeal fashion as has been the norm for the past 40 years.

Temporal variation similarly received very little attention in the models we surveyed. In some systems, seasonal variation in transmission and disease has been shown to correlate with climatological drivers, such as rainfall and temperature [39]. In other systems where disease is endemic, models have rarely been leveraged to investigate the causes of interannual variation in epidemics and their severity, with the exception of models that have accounted for the additional forcing that may be related to El Niño and other interannual climatological drivers [70]. At extremely fine temporal scales, temperature fluctuations within a day were recently shown to have consequences for factors such as pathogen latency in mosquitoes [71,72], yet this variation has not been considered in models either.

### 4.3. Model complexity

One possible reason the aspects of transmission we identified have not been more thoroughly addressed is that these features are inherently more difficult to parametrize than others. Much of the challenge lies in the fact that it is difficult to identify the level of spatial and temporal detail that is necessary in a model while simultaneously collecting sufficient data to parametrize those levels of detail. To know what level of detail is sufficient, one needs a model that allows for excessive detail and a considerable amount of data to parametrize it. Only then will an optimum become identifiable. After gaining some understanding of how transmission behaves at different scales, a secondary goal could be to identify and evaluate targeted control efforts that take advantage of the natural scales of transmission. The quest for this type of understanding dates back to Ross [33] but remains elusive due in part to the fact that it has gone unnoticed by many modelling studies. The vast majority of models either did not include data, or the data–model combination was designed only to estimate one to two parameters at a time (82%, 318/388, electronic supplementary material, table S6).

The issue of deciding on the appropriate level of detail to include in a model, however, depends very much on its purpose. A relevant philosophical perspective to bear in mind when interpreting results from our inventory is Levins' idea that inherent trade-offs exist between different types of models, namely along three axes: realism, generality and precision [73]. For example, the models of Ross and Macdonald could be described as only somewhat realistic, quite general and imprecise. By contrast, the Garki's model [39] is a good example of a model that is realistic and precise, but of limited generality. It is important to note that models at any of these extremes should not be construed as being inherently more or less valuable based simply on this premise. Simple, directed analyses often yield more meaningful results than ones that incorporate interesting, but empirically unjustified complexities, though complex models are, in some cases, justified on the basis of available data and the intent of the model. In either case, the complexity of a model should be dictated by its purpose, and it should be recognized that theory as a whole advances when consensus is built around principles that have support from these varied approaches.

### 4.4. The legacy of Ross and Macdonald

Our *RM* index analysis shows that the models developed by Ross, Macdonald and others in the early- to mid-twentieth century have left an indelible mark on modern theory. The reasons for this influence are clear. The models are biologically motivated, they helped make advances in guiding public health policy for malaria during the GMEP, and they are simple enough to provide a common language for scientists, public health professionals and policy-makers working towards a common goal.

In many ways, the essence and appeal of the Ross–Macdonald theory of transmission can be distilled down to a single quantity: vectorial capacity. This influential quantity attempts to summarize the extent to which mosquitoes propagate pathogens among hosts and is at the heart of the basic reproductive number for mosquito-borne pathogens. Moreover, its formulation is parsimonious, relying on parameter averages and linear relationships, and it allows for straightforward prediction of the efficacy of control measures via the estimation of component parameters and consideration of their exponentiation. For example, on this basis, the effect of reducing adult mosquito survival is expected to grow approximately cubically with additional control efforts, whereas reducing mosquito densities via larval habitat reduction is expected to only have a linear effect [74].

Insightful as these metrics may appear to be, it is important to bear in mind that they are only useful to the extent that they reflect reality. For instance, the impact of control on transmission depends not only on how changes in a parameter affect a quantity such as vectorial capacity but also on how much variation there is in those parameters to begin with. Given limited resources to implement controls, the question of how much of a monetary or operational commitment is required to effect a given change in a parameter is also crucial, yet only 13 models in our inventory considered costs or operational constraints of control (see the electronic supplementary material, table S66). Even more troubling is the fact that estimates of vectorial capacity based on estimates of its component parameters tend to be inaccurate [75] and to not scale properly with transmission intensity [76]. These significant obstacles to translating a simple theory into actionable policy recommendations in a complex world all point towards the need to recast modern theory around the empirically supported complexities that our inventory suggests have been largely ignored.

Advancing theory in this way will require not only the ability to pose the right question or to construct an appropriate model but also the means to analyse and interpret those models. In the past 40 years, the most prominent analyses have centred around thresholds (e.g. *R*_0_) and steady states (e.g. equilibrium disease prevalence; electronic supplementary material, tables S16 and S67). These metrics are indeed useful for gaining insights into the biological factors that contribute to transmission and the promise of control measures, but this is true only insofar as these metrics properly capture necessary nuances of the transmission process. For questions involving spatial, temporal, and inter-individual heterogeneities, underused tools must be leveraged (e.g. matrix representations of the basic reproductive number, time- or space-varying estimates of the force of infection) and new concepts and metrics developed. The seeds for this new generation of theoretical innovation have been sewn by exemplary papers highlighted herein. It is now time to look to them as the basis for developing new models, designing experiments and answering questions of scientific and medical importance.

### 4.5. A direction forward

There appears to be a need for the theory of mosquito-borne pathogen transmission to identify ecological conditions under which the Ross–Macdonald model can be appropriately applied, as well as to extend the theory and develop new means of analysis when those conditions are not met. The Ross–Macdonald framework has provided tremendous insights since its inception over 100 years ago, but routine and uncritical application may now be limiting progress. Moving forward will require a greater emphasis on variation in individual host attributes and their consequences for heterogeneous biting, the concept of poorly mixed mosquito-host encounters and temporal variation. Although these concepts have been successfully addressed at times, they have not been widely appreciated or used. Focusing more modelling effort on these heterogeneities should elevate the theory of mosquito-borne pathogen transmission, making it more robust, accurate and useful for addressing the profound public health challenges currently posed by mosquito-borne pathogens.

## Supplementary Material

Supporting Information 1

## Supplementary Material

Supporting Information 2

## Supplementary Material

Supporting Information 3

## Supplementary Material

Supporting Information 4
