# Supplementary material for: A systematic review of mathematical models of mosquito-borne pathogen transmission: 1970–2010
Source: J R Soc Interface. 2013 Apr 6;10(81):20120921. doi: 10.1098/rsif.2012.0921 (PMC3627099; doi:10.1098/rsif.2012.0921)
Supplement: Supporting Information 1 [file rsif20120921supp1.pdf]

References

[1] L J Abu-Raddad, P Patnaik, and J G Kublin. Dual infection with HIV and malaria fuels the spread of both diseases in sub-Saharan Africa. *Science*, 314(5805):1603–1606, 2006.

[2] B Adams and M Boots. Modelling the relationship between antibody-dependent enhancement and immunological distance with application to dengue. *Journal of Theoretical Biology*, 242(2):337–346, 2006.

[3] B Adams and M Boots. How important is vertical transmission in mosquitoes for the persistence of dengue? Insights from a mathematical model. *Epidemics*, 2:1–10, 2010.

[4] B Adams, E C Holmes, C Zhang, M P Mammen, S Nimmannitya, S Kalayanarooj, and M Boots. Cross-protective immunity can account for the alternating epidemic pattern of dengue virus serotypes circulating in Bangkok. *Proceedings of the National Academy of Sciences of the United States of America*, 103(38):14234–14239, 2006.

[5] B Adams and D D Kapan. Man bites mosquito: understanding the contribution of human movement to vector-borne disease dynamics. *PLoS One*, 4(8):e6763, 2009.

[6] G Adler. A deterministic model of vector-borne epidemics using partial differential equations. *Mathematical Biosciences*, 28:301–320, 1976.

[7] R Aguas, L J White, R W Snow, and M G Gomes. Prospects for malaria eradication in sub-Saharan Africa. *PLoS One*, 3(3):e1767, 2008.

[8] M Aguiar, N Stollenwerk, and B W Kooi. Torus bifurcations, isolas and chaotic attractors in a simple dengue fever model with ADE and temporary cross immunity. *Int. J. Computer Math.*, 86(10&11):1867–1877, 2009.

[9] D Alonso, M J Bouma, and M Pascual. Epidemic malaria and warmer temperatures in recent decades in an East African highland. *Proc Biol Sci*, 2010.

[10] S J Aneke. Mathematical modelling of drug resistant malaria parasites and vector populations. *Mathematical Methods in the Applied Sciences*, 25(4):335–346, 2002.

[11] K C Ang and Z Li. Modelling the spread of dengue in Singapore, 1999.

[12] F Ariey, J B Duchemin, and V Robert. Metapopulation concepts applied to falciparum malaria and their impacts on the emergence and spread of chloroquine resistance. *Infect Genet Evol*, 2(3):185–192, 2003.

[13] J Arino, C Bowman, A Gumel, and S Portet. Effect of pathogen-resistant vectors on the transmission dynamics of a vector-borne disease. *J. Biol. Dynamics*, 1:320–346, 2007.

[14] J L Aron. Dynamics of Acquired-Immunity Boosted by Exposure to Infection. *Mathematical Biosciences*, 64(2):249–259, 1983.

[15] J L Aron. Mathematical modeling of immunity to malaria. *Mathematical Biosciences*, 90(1-2):385–396, 1988.

[16] J L Aron and R M May. The population dynamics of malaria. In R M Anderson, editor, *Population Dynamics and Infectious Disease*, chapter 5, pages 139–179. Chapman and Hall, London, UK, 1982.

[17] Joan L Aron. Acquired immunity dependent upon exposure in an SIRS epidemic model. *Mathematical Biosciences*, 88(1):37–47, 1988.

[18] Y Artzy-Randrup, D Alonso, and M Pascual. Transmission intensity and drug resistance in malaria population dynamics: implications for climate change. *PLoS One*, 5(10):e13588, 2010.

[19] M P Atkinson, Z Su, N Alphey, L S Alphey, P G Coleman, and L M Wein. Analyzing the control of mosquito-borne diseases by a dominant lethal genetic system. *Proceedings of the National Academy of Sciences of the United States of America*, 104(22):9540–9545, 2007.

[20] P Auger, E Kouokam, G Sallet, M Tchuente, and B Tsanou. The Ross-Macdonald model in a patchy environment. *Mathematical Biosciences*, 216(2):123–131, 2008.

[21] N Bacaer. Approximation of the basic reproduction number R0 for vector-borne diseases with a periodic vector population. *Bulletin of Mathematical Biology*, 69(3):1067–1091, 2007.

[22] N Bacaer and C Sokhna. A reaction-diffusion system modeling the spread of resistance to an antimalarial drug. *Mathematical Biosciences and Engineering*, 2(2):227–238, 2005.

[23] F Ball. The threshold behaviour of epidemic models. *J. Appl. Prob.*, 20:227–241, 1983.

[24] L M Bartley, C A Donnelly, and G P Garnett. The seasonal pattern of dengue in endemic areas: Mathematical models of mechanisms. *Transactions of the Royal Society of Tropical Medicine and Hygiene*, 96(4):387–397, 2002.

[25] M G Basanez and D J Rodriguez. Dinamica de transmision y modelos matematicos en enfermedades transmitidas por vectores. *Entomotropica*, 19(3):113–134, 2004.

[26] S Bianco, L B Shaw, and I B Schwartz. Epidemics with multistrain interactions: the interplay between cross immunity and antibody-dependent enhancement. *Chaos*, 19(4):43123, 2009.

[27] D J Bicout and P Sabatier. Mapping Rift Valley fever vectors and prevalence using rainfall variations. *Vector-Borne and Zoonotic Diseases*, 4(1):33–42, 2004.

[28] L Billings, A Fiorillo, and I B Schwartz. Vaccinations in disease models with antibody-dependent enhancement. *Math Biosci*, 211(2):265–281, 2008.

[29] L Billings, I B Schwartz, L B Shaw, M McCrary, D S Burke, and D A T Cummings. Instabilities in multiserotype disease models with antibody-dependent enhancement. *Journal of Theoretical Biology*, 246(1):18–27, 2007.

[30] K Blayneh, Y Z Cao, and H D Kwon. Optimal control of vector-borne diseases: Treatment and prevention. *Discrete and Continuous Dynamical Systems-Series B*, 11(3):587–611, 2009.

[31] Kbenesh W Blayneh, Abba B Gumel, Suzanne Lenhart, and Tim Clayton. Backward bifurcation and optimal control in transmission dynamics of west nile virus. *Bulletin of Mathematical Biology*, 72(4):1006–1028, 2010.

[32] A Bomblies, J B Duchemin, and E A B Eltahir. Hydrology of malaria: Model development and application to a Sahelian village. *Water Resources Research*, 44(12):W12445, 2008.

[33] A Bomblies and E A Eltahir. Assessment of the impact of climate shifts on malaria transmission in the Sahel. *Ecohealth*, 6(3):426–437, 2009.

[34] M F Boni, D L Smith, and R Laxminarayan. Benefits of using multiple first-line therapies against malaria. *Proc Natl Acad Sci U S A*, 105(37):14216–14221, 2008.

[35] C Bowman, A B Gumel, P van den Driessche, J Wu, and H Zhu. A mathematical model for assessing control strategies against West Nile virus. *Bulletin of Mathematical Biology*, 67(5):1107–1133, 2005.

[36] K Brugger and F Rubel. Simulation of climate-change scenarios to explain Usutu virus dynamics in Austria. *Preventive Veterinary Medicine*, 88(1):24–31, 2009.

[37] M N Burattini, M Chen, A Chow, F A B Coutinho, K T Goh, L F Lopez, S Ma, and E Massad. Modelling the control strategies against dengue in Singapore. *Epidemiology and Infection*, 136(3):309–319, 2008.

[38] M N Burattini, F A B Coutinho, and E Massad. A hypothesis for explaining single outbreaks (like the Black Death in European cities) of vector-borne infections. *Medical Hypotheses*, 73(1):110–114, 2009.

[39] M N Burattini, E Massad, and F A B Coutinho. Malaria transmission rates estimated from serological data. *Epidemiology and Infection*, 111(3):503–523, 1993.

[40] M N Burattini, E Massad, F A B Coutinho, and R G Baruzzi. Malaria prevalence amongst Brazilian Indians assessed by a new mathematical model. *Epidemiology and Infection*, 111(3):525–537, 1993.

[41] L M Cai, S M Guo, X Z Li, and M Ghosh. Global dynamics of a dengue epidemic mathematical model. *Chaos Solitons & Fractals*, 42(4):2297–2304, 2009.

[42] L.-M. Cai and X.-Z. Li. Global analysis of a vector-host epidemic model with nonlinear incidences. *Appl. Math. Comp.*, 217:3531–3541, 2010.

[43] N Cancre, A Tall, C Rogier, J Faye, O Sarr, J F Trape, A Spiegel, and F Bois. Bayesian analysis of an epidemiologic model of Plasmodium falciparum malaria infection in Ndiop, Senegal. *American Journal of Epidemiology*, 152(8):760–770, 2000.

[44] P Carnevale, M Lallemant, M Molinier, J Mouchet, and J Coz. [The evaluation of the critical levels of malaria transmission in a stable endemic area (author's transl)]. *Rev Epidemiol Sante Publique*, 30(1):49–70, 1982.

[45] R Carter. Spatial simulation of malaria transmission and its control by malaria transmission blocking vaccination. *International Journal for Parasitology*, 32:1617–1624, 2002.

[46] E Chikaki and H Ishikawa. A dengue transmission model in Thailand considering sequential infections with all four serotypes. *J Infect Dev Ctries*, 3(9):711–722, 2009.

[47] N Chitnis, J M Cushing, and J M Hyman. Bifurcation analysis of a mathematical model for malaria transmission. *Siam Journal on Applied Mathematics*, 67(1):24–45, 2006.

[48] N Chitnis, J M Hyman, and J M Cushing. Determining important parameters in the spread of malaria through the sensitivity analysis of a mathematical model. *Bulletin of Mathematical Biology*, 70(5):1272–1296, 2008.

[49] N Chitnis, A Schapira, T Smith, and R Steketee. Comparing the effectiveness of malaria vector-control interventions through a mathematical model. *Am J Trop Med Hyg*, 83(2):230–240, 2010.

[50] N Chitnis, Thomas A Smith, and R Steketeeb. A mathematical model for the dynamics of malaria in mosquitoes feeding on a heterogeneous host population. *Journal of Biological Dynamics*, 2(3):259–285, 2008.

[51] C Chiyaka, W Garira, and S Dube. Transmission model of endemic human malaria in a partially immune population. *Mathematical and Computer Modelling*, 46(5-6):806–822, 2007.

[52] C Chiyaka, W Garira, and S Dube. Effects of treatment and drug resistance on the transmission dynamics of malaria in endemic areas. *Theoretical Population Biology*, 75(1):14–29, 2009.

[53] C Chiyaka, Z Mukandavire, P Das, F Nyabadza, S D Hove-Musekwa, and H Mwambi. Theoretical analysis of mixed Plasmodium malariae and Plasmodium falciparum infections with partial cross-immunity. *J Theor Biol*, 263(2):169–178, 2010.

[54] C Chiyaka, J M Tchuenche, W Garira, and S Dube. A mathematical analysis of the effects of control strategies on the transmission dynamics of malaria. *Applied Mathematics and Computation*, 195(2):641–662, 2008.

[55] Y H Choi, C Comiskey, M D A Lindsay, J A Cross, and M Anderson. Modelling the transmission dynamics of Ross River virus in Southwestern Australia. *IMA Journal of Mathematics Applied in Medicine and Biology*, 19(1):61–74, 2002.

[56] G Chowell, P Diaz-Duenas, J C Miller, A Alcazar-Velazco, J M Hyman, P W Fenimore, and C Castillo-Chavez. Estimation of the reproduction number of dengue fever from spatial epidemic data. *Mathematical Biosciences*, 208(2):571–589, 2007.

[57] S Cirino and J A L Da Silva. Modelo Epidemiologico SEIR de Transmissao da Dengue em Redes de Populacoes Acopladas. *Tend. Mat. Apl. Comput.*, 5(1):55–64, 2004.

[58] G E Coelho, M N Burattini, G Teixeira Mda, F A Coutinho, and E Massad. Dynamics of the 2006/2007 dengue outbreak in Brazil. *Mem Inst Oswaldo Cruz*, 103(6):535–539, 2008.

[59] J M Cohen, B Moonen, R W Snow, and D L Smith. How absolute is zero? An evaluation of historical and current definitions of malaria elimination. *Malar J*, 9:213, 2010.

[60] D Collett and M S Lye. Modelling the effect of intervention on the transmission of malaria in East Malaysia. *Stat Med*, 6(7):853–861, 1987.

[61] C Cosner, J C Beier, R S Cantrell, D Impoinvil, L Kapitanski, M D Potts, A Troyo, and S Ruan. The effects of human movement on the persistence of vector-borne diseases. *Journal of Theoretical Biology*, 258(4):550–560, 2009.

[62] F A Coutinho, M N Burattini, L F Lopez, and E Massad. Threshold conditions for a non-autonomous epidemic system describing the population dynamics of dengue. *Bull Math Biol*, 68(8):2263–2282, 2006.

[63] F A B Coutinho, M N Burattini, L F Lopez, and E Massad. An approximate threshold condition for non-autonomous system: An application to a vector-borne infection. *Mathematics and Computers in Simulation*, 70(3):149–158, 2005.

[64] M H Craig, R W Snow, and D le Sueur. A climate-based distribution model of malaria transmission in sub-Saharan Africa. *Parasitol Today*, 15(3):105–111, 1999.

[65] A P Cross and B Singer. Modeling the development of resistance of Plasmodium falciparum to antimalarial drugs. *Transactions of the Royal Society of Tropical Medicine and Hygiene*, 85(3):349–355, 1991.

[66] G Cruz-Pacheco, L Esteva, J A Montano-Hirose, and C Vargas. Modelling the dynamics of West Nile Virus. *Bulletin of Mathematical Biology*, 67(6):1157–1172, 2005.

[67] Gustavo Cruz-Pacheco, Lourdes Esteva, and Cristobal Vargas. Seasonality and outbreaks in West Nile virus infection. *Bull. Math. Biol.*, 71(6):1378–1393, 2009.

[68] D A Cummings, S Iamsirithaworn, J T Lessler, A McDermott, R Prasanthong, A Nisalak, R G Jarman, D S Burke, and R V Gibbons. The impact of the demographic transition on dengue in Thailand: insights from a statistical analysis and mathematical modeling. *PLoS Med*, 6(9):e1000139, 2009.

[69] D A Cummings, I B Schwartz, L Billings, L B Shaw, and D S Burke. Dynamic effects of antibody-dependent enhancement on the fitness of viruses. *Proc Natl Acad Sci U S A*, 102(42):15259–15264, 2005.

[70] T De-Camino-Beck, M A Lewis, and P van den Driessche. A graph-theoretic method for the basic reproduction number in continuous time epidemiological models. *Journal of Mathematical Biology*, 59(4):503–516, 2009.

[71] P P de Moor and F E Steffens. Computer-simulated model of an arthropod-borne virus transmission cycle, with special reference to chikungunya virus. *Transactions of the Royal Society of Tropical Medicine and Hygiene*, 64(6):927–934, 1970.

[72] N Degallier, C Favier, J P Boulanger, and C Menkes. Imported and autochthonous cases in the dynamics of dengue epidemics in Brazil. *Rev Saude Publica*, 43(1):1–7, 2009.

[73] M Derouich and A Boutayeb. Dengue fever: Mathematical modelling and computer simulation. *Applied Mathematics and Computation*, 177(2):528–544, 2006.

[74] M Derouich, A Boutayeb, and E H Twizell. A model of dengue fever. *Biomed Eng Online*, 2:4, 2003.

[75] A P K Dezoysa, C Mendis, A C Gamagemendis, S Weerasinghe, P R J Herath, and K N Mendis. A mathematical model for Plasmodium vivax malaria transmission: Estimation of the impact of transmission-blocking immunity in an endemic area . *Bulletin of the World Health Organization*, 69(6):725–734, 1991.

[76] K Dietz. Transmission and control of arbovirus diseases. In D Ludwig and K L Cooke, editors, *Epidemiology*, pages 104–121. SIAM, Philadelphia, 1975.

[77] K. Dietz. *Models for vector-borne parasitic diseases*, pages 264–277. Lecture Notes in Biomathematics. Springer-Verlag, Berlin, 1980.

[78] K Dietz. Mathematical models for transmission and control of malaria. In W Wernsdorfer and I McGregor, editors, *Principles and Practice of Malaria*, chapter 37, pages 1091–1133. Churchill Livingstone, Edinburgh, UK, 1988.

[79] K Dietz. The estimation of the basic reproduction number for infectious diseases. *Statistical Methods in Medical Research*, 2(1):23–41, 1993.

[80] K Dietz, L Molineaux, and A Thomas. A malaria model tested in the African savannah. *Bull World Health Organ*, 50(3-4):347–357, 1974.

[81] Klaus Dietz. Malaria Models. *Advances in Applied Probability*, pages 208–210, 1971.

[82] A Dobson and J Foufopoulos. Emerging infectious pathogens of wildlife. *Philosophical Transactions of the Royal Society of London Series B-Biological Sciences*, 356(1411):1001–1012, 2001.

[83] C J Drakeley, P H Corran, P G Coleman, J E Tongren, S L McDonald, I Carneiro, R Malima, J Lusingu, A Manjurano, W M Nkya, M M Lemnge, J Cox, H Reyburn, and E M Riley. Estimating medium- and long-term trends in malaria transmission by using serological markers of malaria exposure. *Proc Natl Acad Sci U S A*, 102(14):5108–5113, 2005.

[84] H P Duerr, K Dietz, and M Eichner. Determinants of the eradicability of filarial infections: a conceptual approach. *Trends in Parasitology*, 21(2):88–96, 2005.

[85] J E Duque, M A Navarro-Silva, and D Y Trejos. Simulating management of Aedes aegypti(Diptera: Culicidae)and its effects in a dengue epidemic. *Revista Colombiana De Entomologia*, 35(1):66–72, 2009.

[86] C Dye. Vectorial capacity: must we measure all its components? *Parasitol Today*, 2(8):203–209, 1986.

[87] C Dye. The analysis of parasite transmission by bloodsucking insects. *Annu Rev Entomol*, 37:1–19, 1992.

[88] C Dye. Approaches to vector control: new and trusted. 5. The epidemiological context of vector control. *Trans R Soc Trop Med Hyg*, 88(2):147–149, 1994.

[89] C Dye and G Hasibeder. Population dynamics of mosquito-borne disease: effects of flies which bite some people more frequently than others. *Trans R Soc Trop Med Hyg*, 80(1):69–77, 1986.

[90] C Dye and B G Williams. Non-linearities in the dynamics of indirectly-transmitted infections (or, Does having a vector make a difference?). In B T Grenfell and A P Dobson, editors, *Ecology of Infectious Diseases in Natural Populations*, pages 260–279. Cambridge University Press, Cambridge, UK, 1995.

[91] R A Erickson, S M Presley, L J S Allen, K R Long, and S B Cox. A dengue model with a dynamic Aedes albopictus vector population. *Ecological Modelling*, 221(24):2899–2908, 2010.

[92] L Esteva and C Vargas. Analysis of a dengue disease transmission model. *Math Biosci*, 150(2):131–151, 1998.

[93] L Esteva and C Vargas. A model for dengue disease with variable human population. *J Math Biol*, 38(3):220–240, 1999.

[94] L Esteva and C Vargas. Influence of vertical and mechanical transmission on the dynamics of dengue disease. *Math Biosci*, 167(1):51–64, 2000.

[95] L Esteva and C Vargas. Coexistence of different serotypes of dengue virus. *Journal of Mathematical Biology*, 46(1):31–47, 2003.

[96] C Favier, K Chalvet-Monfray, P Sabatier, R Lancelot, D Fontenille, and M A Dubois. Rift Valley fever in West Africa: the role of space in endemicity. *Tropical Medicine & International Health*, 11(12):1878–1888, 2006.

[97] C Favier, N Degallier, M G Rosa-Freitas, J P Boulanger, J R Costa Lima, J F Luitgards-Moura, C E Menkes, B Mondet, C Oliveira, E T S Weimann, and P Tsouris. Early determination of the reproductive number for vector-borne diseases: The case of dengue in Brazil. *Tropical Medicine & International Health*, 11(3):332–340, 2006.

[98] C Favier, D Schmit, Muller-Graf C D., B Cazelles, N Degallier, B Mondet, and M A Dubois. Influence of spatial heterogeneity on an emerging infectious disease: the case of dengue epidemics. *Proc Biol Sci*, 272(1568):1171–1177, 2005.

[99] Z L Feng and J X Velasco-Hernandez. Competitive exclusion in a vector-host model for the dengue fever. *Journal of Mathematical Biology*, 35(5):523–544, 1997.

[100] N Ferguson, R Anderson, and S Gupta. The effect of antibody-dependent enhancement on the transmission dynamics and persistence of multiple-strain pathogens. *Proc.Natl.Acad.Sci.U.S.A*, 96(2):790–794, 1999.

[101] N M Ferguson, C A Donnelly, and R M Anderson. Transmission dynamics and epidemiology of dengue: insights from age-stratified sero-prevalence surveys. *Philos Trans R Soc Lond B Biol Sci*, 354(1384):757–768, 1999.

[102] J A N Filipe, E M Riley, C J Drakeley, C J Sutherland, and A C Ghani. Determination of the processes driving the acquisition of immunity to malaria using a mathematical transmission model. *Plos Computational Biology*, 3(12):2569–2579, 2007.

[103] D A Focks, R J Brenner, J Hayes, and E Daniels. Transmission thresholds for dengue in terms of Aedes aegypti pupae per person with discussion of their utility in source reduction efforts. *American Journal of Tropical Medicine and Hygiene*, 62(1):11–18, 2000.

[104] D A Focks, E Daniels, D G Haile, and J E Keesling. A simulation model of the epidemiology of urban dengue fever: Literature analysis, model development, preliminary validation, and samples of simulation results. *American Journal of Tropical Medicine and Hygiene*, 53(5):489–506, 1995.

[105] Dana A Focks, Richard J Brenner, Dave D Chadee, and James H Trosper. The use of spatial analysis in the control and risk assessment of vector-borne diseases. *American Entomologist*, 45(3):173–183, 1999.

[106] J Freeman, K F Laserson, I Petralanda, and A Spielman. Effect of chemotherapy on malaria transmission among Yanomani Amerindians: Simulated consequences of placebo treatment. *American Journal of Tropical Medicine and Hygiene*, 60(5):774–780, 1999.

[107] H D Gaff, D M Hartley, and Nicole P Leahy. An epidemiological model of Rift Valley fever. *Electronic Journal of Differential Equations*, 2007:115, 2007.

[108] H F Gagliardi, F A B da Silva, and D Alves. Automata network simulator applied to the epidemiology of urban dengue fever. pages 297–304, 2006.

[109] M Gambhir and E Michael. Complex ecological dynamics and eradicability of the vector borne macroparasitic disease, lymphatic filariasis. *PLoS One*, 3(8):e2874, 2008.

[110] S Gandon, M J Mackinnon, S Nee, and A F Read. Imperfect vaccines and the evolution of pathogen virulence. *Nature*, 414(6865):751–756, 2001.

[111] S M Garba and A B Gumel. Effect of cross-immunity on the transmission dynamics of two strains of dengue. *Int. J. Computer Math.*, 87(10):2361–2384, 2010.

[112] S M Garba, A B Gumel, and M R Abu Bakar. Backward bifurcations in dengue transmission dynamics. *Mathematical Biosciences*, 215(1):11–25, 2008.

[113] J Gaudart, O Toure, N Dessay, A L Dicko, S Ranque, L Forest, J Demongeot, and O K Doumbo. Modelling malaria incidence with environmental dependency in a locality of Sudanese savannah area, Mali. *Malaria Journal*, 8:61, 2009.

[114] A Gemperli, P Vounatsou, N Sogoba, and T Smith. Malaria mapping using transmission models: application to survey data from Mali. *Am J Epidemiol*, 163(3):289–297, 2006.

[115] P W Gething, D L Smith, A P Patil, A J Tatem, R W Snow, and S I Hay. Climate change and the global malaria recession. *Nature*, 465(7296):342–345, 2010.

[116] A C Ghani, C J Sutherland, E M Riley, C J Drakeley, J T Griffin, R D Gosling, and J A Filipe. Loss of population levels of immunity to malaria as a result of exposure-reducing interventions: consequences for interpretation of disease trends. *PLoS One*, 4(2):e4383, 2009.

[117] A K Ghosh and P K Tapaswi. Dynamics of Japanese encephalitis: A study in mathematical epidemiology. *IMA Journal of Mathematics Applied in Medicine and Biology*, 16(1):1–27, 1999.

[118] K Glass. Ecological mechanisms that promote arbovirus survival: a mathematical model of Ross River virus transmission. *Transactions of the Royal Society of Tropical Medicine and Hygiene*, 99(4):252–260, 2005.

[119] R D Gosling, A C Ghani, J L Deen, L von Seidlein, B M Greenwood, and D Chandramohan. Can changes in malaria transmission intensity explain prolonged protection and contribute to high protective efficacy of intermittent preventive treatment for malaria in infants? *Malaria Journal*, 7:54, 2008.

[120] S A Gourley, R S Liu, and J H Wu. Eradicating vector-borne diseases via age-structured culling. *Journal of Mathematical Biology*, 54(3):309–335, 2007.

[121] J T Griffin, T D Hollingsworth, L C Okell, T S Churcher, M White, W Hinsley, T Bousema, C J Drakeley, N M Ferguson, M G Basanez, and A C Ghani. Reducing Plasmodium falciparum malaria transmission in Africa: a model-based evaluation of intervention strategies. *PLoS Med*, 7(8), 2010.

[122] D A Griffiths. A bivariate birth-death process which approximates to the spread of a disease involving a vector. *J. Appl. Prob.*, 9:65–75, 1972.

[123] W Gu, C M Mbogo, J I Githure, J L Regens, G F Killeen, C M Swalm, G Yan, and J C Beier. Low recovery rates stabilize malaria endemicity in areas of low transmission in coastal Kenya. *Acta Trop*, 86(1):71–81, 2003.

[124] W Gu and R J Novak. Habitat-based modeling of impacts of mosquito larval interventions on entomological inoculation rates, incidence, and prevalence of malaria. *Am J Trop Med Hyg*, 73(3):546–552, 2005.

[125] W Gu and R J Novak. Agent-based modelling of mosquito foraging behaviour for malaria control. *Trans R Soc Trop Med Hyg*, 103(11):1105–1112, 2009.

[126] W Gu and R J Novak. Predicting the impact of insecticide-treated bed nets on malaria transmission: the devil is in the detail. *Malar J*, 8:256, 2009.

[127] W D Gu, J L Regens, J C Beier, and R J Novak. Source reduction of mosquito larval habitats has unexpected consequences on malaria transmission. *Proceedings of the National Academy of Sciences of the United States of America*, 103(46):17560–17563, 2006.

[128] H L Guyatt, R W Snow, and D B Evans. Malaria epidemiology and economics: the effect of delayed immune acquisition on the cost-effectiveness of insecticide-treated bednets. *Philos Trans R Soc Lond B Biol Sci*, 354(1384):827–835, 1999.

[129] R Hagmann, J D Charlwood, V Gil, C Ferreira, V do Rosario, and T A Smith. Malaria and its possible control on the island of Principe. *Malaria Journal*, 2:15, 2003.

[130] M E Halloran and C J Struchiner. Modeling transmission dynamics of stage-specific malaria vaccines. *Parasitol Today*, 8(3):77–85, 1992.

[131] M E Halloran, C J Struchiner, and A Spielman. Modeling malaria vaccines. II: Population effects of stage-specific malaria vaccines dependent on natural boosting. *Math Biosci*, 94(1):115–149, 1989.

[132] P A Hancock and H C J Godfray. Application of the lumped age-class technique to studying the dynamics of malaria-mosquito-human interactions. *Malaria Journal*, 6:98, 2007.

[133] P A Hancock, M B Thomas, and H C J Godfray. An age-structured model to evaluate the potential of novel malaria-control interventions: a case study of fungal biopesticide sprays. *Proceedings of the Royal Society of London Series B-Biological Sciences*, 276:71–80, 2009.

[134] N A Hartemink, S A Davis, P Reiter, Z Hubalek, and J A P Heesterbeek. Importance of bird-to-bird transmission for the establishment of West Nile virus. *Vector-Borne and Zoonotic Diseases*, 7(4):575–584, 2007.

[135] G Hasibeder and C Dye. Population dynamics of mosquito-borne disease: persistence in a completely heterogeneous environment. *Theor Popul Biol*, 33(1):31–53, 1988.

[136] G R Hosack, P A Rossignol, and P van den Driessche. The control of vector-borne disease epidemics. *Journal of Theoretical Biology*, 255(1):16–25, 2008.

[137] M B Hoshen and A P Morse. A weather-driven model of malaria transmission. *Malaria Journal*, 3:32, 2004.

[138] X L Hu, Y S Liu, and J H Wu. Culling structured hosts to eradicate vector-borne diseases. *Mathematical Biosciences and Engineering*, 6(2):301–319, 2009.

[139] H Ishikawa, A Ishii, N Nagai, H Ohmae, M Harada, S Suguri, and J Leafasia. A mathematical model for the transmission of Plasmodium vivax malaria. *Parasitology International*, 52(1):81–93, 2003.

[140] M A Janssen and W J Martens. Modeling malaria as a complex adaptive system. *Artif Life*, 3(3):213–236, 1997.

[141] T H Jetten, W J M Martens, and W Takken. Model simulations to estimate malaria risk under climate change. *Journal of Medical Entomology*, 33(3):361–371, 1996.

[142] J F Jiang and Z P Qiu. The complete classification for dynamics in a nine-dimensional West Nile virus model. *Siam Journal on Applied Mathematics*, 69(5):1205–1227, 2009.

[143] J F Jiang, Z P Qiu, J H Wu, and H P Zhu. Threshold Conditions for West Nile Virus Outbreaks. *Bulletin of Mathematical Biology*, 71:627–647, 2009.

[144] A Kammanee, N Kanyamee, and I M Tang. Basic reproduction number for the transmission of Plasmodium vivax malaria. *Southeast Asian J Trop Med Public Health*, 32(4):702–706, 2001.

[145] I Kawaguchi, A Sasaki, and M Boots. Why are dengue virus serotypes so distantly related? Enhancement and limiting serotype similarity between dengue virus strains. *Proceedings of the Royal Society of London Series B-Biological Sciences*, 270(1530):2241–2247, 2003.

[146] I Kawaguchi, A Sasaki, and M Mogi. Combining zooprophylaxis and insecticide spraying: a malaria-control strategy limiting the development of insecticide resistance in vector mosquitoes. *Proc Biol Sci*, 271(1536):301–309, 2004.

[147] B H Kay, A J Saul, and A McCullagh. A mathematical model for the rural amplification of Murray Valley encephalitis virus in southern Australia. *American Journal of Epidemiology*, 125(4):690–705, 1987.

[148] V M Kenkre, R R Parmenter, L D Peixoto, and L Sadasiv. A theoretical framework for the analysis of the West Nile virus epidemic. *Mathematical and Computer Modelling*, 42(3-4):313–324, 2005.

[149] G F Killeen, J Kihonda, E Lyimo, F R Oketch, M E Kotas, E Mathenge, J A Schellenberg, C Lengeler, T A Smith, and C J Drakeley. Quantifying behavioural interactions between humans and mosquitoes: evaluating the protective efficacy of insecticidal nets against malaria transmission in rural Tanzania. *BMC Infect Dis*, 6:161, 2006.

[150] G F Killeen, B G Knols, and W Gu. Taking malaria transmission out of the bottle: implications of mosquito dispersal for vector-control interventions. *Lancet Infect Dis*, 3(5):297–303, 2003.

[151] G F Killeen, F E McKenzie, B D Foy, C Bogh, and J C Beier. The availability of potential hosts as a determinant of feeding behaviours and malaria transmission by African mosquito populations. *Trans R Soc Trop Med Hyg*, 95(5):469–476, 2001.

[152] G F Killeen, F E McKenzie, B D Foy, C Schieffelin, P F Billingsley, and J C Beier. A simplified model for predicting malaria entomologic inoculation rates based on entomologic and parasitologic parameters relevant to control. *American Journal of Tropical Medicine and Hygiene*, 62(5):535–544, 2000.

[153] G F Killeen, F E McKenzie, B D Foy, C Schieffelin, P F Billingsley, and J C Beier. The potential impact of integrated malaria transmission control on entomologic inoculation rate in highly endemic areas. *American Journal of Tropical Medicine and Hygiene*, 62(5):545–551, 2000.

[154] G F Killeen, A Seyoum, and B G Knols. Rationalizing historical successes of malaria control in Africa in terms of mosquito resource availability management. *Am J Trop Med Hyg*, 71(2 Suppl):87–93, 2004.

[155] G F Killeen and T A Smith. Exploring the contributions of bed nets, cattle, insecticides, and excitorepellency to malaria control: A deterministic model of mosquito host-seeking behaviour and mortality. *Transactions of the Royal Society of Tropical Medicine and Hygiene*, 101(9):867–880, 2007.

[156] G F Killeen, T A Smith, H M Ferguson, H Mshinda, S Abdulla, C Lengeler, and S P Kachur. Preventing childhood malaria in Africa by protecting adults from mosquitoes with insecticide-treated nets. *PLoS Med*, 4(7):e229, 2007.

[157] A M Kilpatrick, P Daszak, S J Goodman, H Rogg, L D Kramer, V Cedeno, and A A Cunningham. Predicting pathogen introduction: West Nile virus spread to Galaipagos. *Conserv Biol*, 20(4):1224–1231, 2006.

[158] Joel Kingsolver. Mosquito Host Choice and the Epidemiology of Malaria. *The American Naturalist*, 130(6):811–827, 1987.

[159] A E Kiszewski and A Spielman. Virulence of vector-borne pathogens. A stochastic automata model of perpetuation. *Ann N Y Acad Sci*, 740:249–259, 1994.

[160] E Y Klein, D L Smith, M F Boni, and R Laxminarayan. Clinically immune hosts as a refuge for drug-sensitive malaria parasites. *Malaria Journal*, 7:67, 2008.

[161] J C Koella. On the use of mathematical models of malaria transmission. *Acta Trop*, 49(1):1–25, 1991.

[162] J C Koella and R Antia. Epidemiological models for the spread of anti-malarial resistance. *Malaria Journal*, 2:3, 2003.

[163] J C Koella and C Boete. A model for the coevolution of immunity and immune evasion in vector-borne diseases with implications for the epidemiology of malaria. *Am Nat*, 161(5):698–707, 2003.

[164] J C Koella and L Zaghloul. Using evolutionary costs to enhance the efficacy of malaria control via genetically manipulated mosquitoes. *Parasitology*, 135(13):1489–1496, 2008.

[165] R Kongnuy and P Pongsumpun. Mathematical Modeling for Dengue Transmission with the Effect of Season. *International Journal of Biological and Medical Sciences*, 5(2):74–78, 2010.

[166] S Lawpoolsri, E Y Klein, P Singhasivanon, S Yimsamran, N Thanyavanich, W Maneeboonyang, L L Hungerford, J H Maguire, and D L Smith. Optimally timing primaquine treatment to reduce Plasmodium falciparum transmission in low endemicity Thai-Myanmar border populations. *Malaria Journal*, 8:159, 2009.

[167] R Laxminarayan. Act now or later? Economics of malaria resistance. *Am J Trop Med Hyg*, 71(2 Suppl):187–195, 2004.

[168] R Laxminarayan, M Over, and D L Smith. Will a global subsidy of new antimalarials delay the emergence of resistance and save lives? *Health Aff (Millwood)*, 25(2):325–336, 2006.

[169] A Le Menach, F E McKenzie, A Flahault, and D L Smith. The unexpected importance of mosquito oviposition behaviour for malaria: Non-productive larval habitats can be sources for malaria transmission. *Malaria Journal*, 4:23, 2005.

[170] A Le Menach, S Takala, F E McKenzie, A Perisse, A Harris, A Flahault, and D L Smith. An elaborated feeding cycle model for reductions in vectorial capacity of night-biting mosquitoes by insecticide-treated nets. *Malar J*, 6:10, 2007.

[171] M A Lewis, M Krkosek, and M Wonham. Dynamics of emerging wildlife disease. In S Sivaloganathan, editor, *New Perspectives in Mathematical Biology*, volume 57, pages 61–82. AMS/Fields Inst., Providence, RI, 2010.

[172] M A Lewis, J Renclawowicz, P van den Driessche, and M Wonham. A comparison of continuous and discrete-time West Nile virus models. *Bulletin of Mathematical Biology*, 68(3):491–509, 2006.

[173] Mark Lewis, Joanna Rencawowicz, and P van Driessche. Traveling Waves and Spread Rates for a West Nile Virus Model. *Bulletin of Mathematical Biology*, 68(1):3–23, 2006.

[174] J Li. A malaria model with partial immunity in humans. *Mathematical Biosciences and Engineering*, 5(4):789–801, 2008.

[175] J Li, R M Welch, U S Nair, T L Sever, D E Irwin, C Cordon-Rosales, and N Padilla. Dynamic malaria models with environmental changes. *Proceedings of the Thirty-Fourth Southeastern Symposium on System Theory*, pages 396–400, 2002.

[176] C Linard, N Poncon, D Fontenille, and E F Lambin. A multi-agent simulation to assess the risk of malaria re-emergence in southern France. *Ecological Modelling*, 220(2):160–174, 2009.

[177] S W Lindsay and M H Birley. Climate change and malaria transmission. *Ann Trop Med Parasitol*, 90(6):573–588, 1996.

[178] S W Lindsay and W J Martens. Malaria in the African highlands: past, present and future. *Bull World Health Organ*, 76(1):33–45, 1998.

[179] H L Liu, H B Xu, J Y Yu, and G T Zhu. Stability results on age-structured SIS epidemic model with coupling impulsive effect. *Discrete Dynamics in Nature and Society*, 2006:1–11, 2006.

[180] H L Liu, J Y Yu, and G T Zhu. Analysis of a vector-host malaria model with impulsive effect and infection-age. *Advances in Complex Systems*, 9(3):237–248, 2006.

[181] R S Liu, J P Shuai, J H Wu, and H P Zhu. Modeling spatial spread of West Nile virus and impact of directional dispersal of birds. *Mathematical Biosciences and Engineering*, 3(1):145–160, 2006.

[182] A L Lloyd, J Zhang, and A M Root. Stochasticity and heterogeneity in host-vector models. *Journal of the Royal Society Interface*, 4(16):851–863, 2007.

[183] Luiz Fernandes Lopez, Francisco Antonio Bezerra Coutinho, Marcelo Nascimento Burattini, and Eduardo Massad. Threshold conditions for infection persistence in complex host-vectors interactions. *Comptes rendus biologies*, 325(11):1073–1084, 2002.

[184] C C Lord and J F Day. Simulation studies of St. Louis encephalitis and West Nile viruses: the impact of bird mortality. *Vector borne and zoonotic diseases (Larchmont, NY)*, 1(4):317–329, 2001.

[185] C C Lord and J F Day. Simulation studies of St. Louis encephalitis virus in south Florida. *Vector borne and zoonotic diseases (Larchmont, NY)*, 1(4):299–315, 2001.

[186] Cynthia C Lord. Seasonal population dynamics and behaviour of insects in models of vector-borne pathogens. *Physiological Entomology*, 29(3):214–222, 2004.

[187] J Lourenço, M Recker, and R Rico-Hesse. Viral and Epidemiological Determinants of the Invasion Dynamics of Novel Dengue Genotypes. *Plos Neglected Tropical Diseases*, 4(11):e894, 2010.

[188] P M Luz, C T Codeco, E Massad, and C J Struchiner. Uncertainties regarding dengue modeling in Rio de Janeiro, Brazil. *Memorias Do Instituto Oswaldo Cruz*, 98(7):871–878, 2003.

[189] N A Maidana and H M Yang. Describing the geographic spread of dengue disease by traveling waves. *Mathematical Biosciences*, 215(1):64–77, 2008.

[190] P Marcati and M A Pozio. Global asymptotic stability for a vector disease model with spatial spread. *J Math Biol*, 9(2):179–187, 1980.

[191] C A Marques, O P Forattini, and E Massad. The Basic Reproduction Number for Dengue Fever in Sao-Paulo State, Brazil - 1990-1991 Epidemic. *Transactions of the Royal Society of Tropical Medicine and Hygiene*, 88(1):58–59, 1994.

[192] P Martens, R S Kovats, S Nijhof, P de Vries, M T J Livermore, D J Bradley, J Cox, and A J McMichael. Climate change and future populations at risk of malaria. *Global Environmental Change-Human and Policy Dimensions*, 9:S89–S107, 1999.

[193] W J Martens. Climate change and malaria: exploring the risks. *Med War*, 11(4):202–213, 1995.

[194] W J M Martens, T H Jetten, J Rotmans, and L W Niessen. Climate-Change and Vector-Borne Diseases - a Global Modeling Perspective. *Global Environmental Change-Human and Policy Dimensions*, 5(3):195–209, 1995.

[195] P H Martin and M G Lefebvre. Malaria and climate: Sensitivity of malaria potential transmission to climate. *Ambio*, 24(4):200–207, 1995.

[196] E Massad, R H Behrens, M N Burattini, and F A B Coutinho. Modeling the risk of malaria for travelers to areas with stable malaria transmission. *Malaria J.*, 8:296, 2009.

[197] E Massad, F A B Coutinho, M N Burattini, and M Amaku. Estimation of Ro from the initial phase of an outbreak of a vector-borne infection. *Trop. Med. Int. Health*, 15(1):120–126, 2010.

[198] E Massad, F A B Coutinho, M N Burattini, and L F Lopez. The risk of yellow fever in a dengue-infested area. *Transactions of the Royal Society of Tropical Medicine and Hygiene*, 95(4):370–374, 2001.

[199] E Massad, F A B COUTINHO, S Ma, and M N Burattini. A hypothesis for the 2007 dengue outbreak in Singapore. *Epidemiology and Infection*, 138(7):951–957, 2009.

[200] E Massad and O P Forattini. Modelling the temperature sensitivity of some physiological parameters of epidemiologic significance. *Ecosystem Health*, 4(2):119–129, 1998.

[201] E Massad, S Ma, M N Burattini, Y Tun, F A B Coutinho, and L W Ang. The risk of chikungunya fever in a dengue-endemic area. *Journal of Travel Medicine*, 15(3):147–155, 2008.

[202] R J Maude, W Pontavornpinyo, S Saralamba, R Aguas, S Yeung, A M Dondorp, N P Day, N J White, and L J White. The last man standing is the most resistant: eliminating artemisinin-resistant malaria in Cambodia. *Malar J*, 8:31, 2009.

[203] F E McKenzie, J K Baird, J C Beier, A A Lal, and W H Bossert. A biologic basis for integrated malaria control. *Am J Trop Med Hyg*, 67(6):571–577, 2002.

[204] F E McKenzie and W H Bossert. An integrated model of Plasmodium falciparum dynamics. *J Theor Biol*, 232(3):411–426, 2005.

[205] F E McKenzie, M U Ferreira, J K Baird, G Snounou, and W H Bossert. Meiotic recombination, cross-reactivity, and persistence in Plasmodium falciparum. *Evolution*, 55(7):1299–1307, 2001.

[206] F E McKenzie, G F Killeen, J C Beier, and W H Bossert. Seasonality, parasite diversity, and local extinctions in Plasmodium falciparum malaria. *Ecology*, 82(10):2673–2681, 2001.

[207] F E McKenzie, R C Wong, and W H Bossert. Discrete-event simulation models of Plasmodium falciparum malaria. *Simulation*, 71(4):250–261, 1998.

[208] F E McKenzie, R C Wong, and W H Bossert. Discrete-event models of mixed-phenotype Plasmodium falciparum malaria. *Simulation*, 73(4):213–217, 1999.

[209] J Medlock, P M Luz, C J Struchiner, and A P Galvani. The impact of transgenic mosquitoes on dengue virulence to humans and mosquitoes. *Am Nat*, 174(4):565–577, 2009.

[210] C Mendis, P R J Herath, J Rajakaruna, S Weerasinghe, A C Gamagemendis, K N Mendis, and A P K Dezoysa. Method to estimate relative transmission efficiencies of Anopheles species (Diptera, Culicidae) in human malaria transmission. *Journal of Medical Entomology*, 29(2):188–196, 1992.

[211] E Michael and D A P Bundy. Herd immunity to filarial infection is a function of vector biting rate. *Proceedings of the Royal Society of London Series B-Biological Sciences*, 265(1399):855–860, 1998.

[212] E Michael and M Gambhir. Transmission models and management of lymphatic filariasis elimination. *Advances in experimental medicine and biology*, 673:157–171, 2010.

[213] E Michael and M Gambhir. Vector transmission heterogeneity and the population dynamics and control of lymphatic filariasis. *Advances in experimental medicine and biology*, 673:13–31, 2010.

[214] E Michael, M Malecela-Lazaro, B Maegga, P Fischer, and J Kazura. Mathematical models and lymphatic filariasis control: monitoring and evaluating interventions. *Trends in Parasitology*, 22(11):529–535, 2006.

[215] E Michael, M N Malecela-Lazaro, and J W Kazura. Epidemiological modelling for monitoring and evaluation of lymphatic filariasis control. *Adv Parasitol*, 65:191–237, 2007.

[216] P J M Milligan and D Y Downham. Models of superinfection and acquired immunity to multiple parasite strains. *Journal of Applied Probability*, 33(4):915–932, 1996.

[217] L Molineaux, K Dietz, and A Thomas. Further epidemiological evaluation of a malaria model. *Bull World Health Organ*, 56(4):565–571, 1978.

[218] L A Mosquera and M H Perera. Modelo matematico para la enfermedad del dengue. *Boletin de Matematicas*, 13(2):176–185, 2006.

[219] I Nå sell. *Hybrid Models of Tropical Infections*, volume 59. Springer-Verlag, Berlin, 1985.

[220] I Nå sell. On superinfection in malaria. *IMA J Math Appl Med Biol*, 3(3):211–227, 1986.

[221] I Nå sell. Malaria infection with relapses and misdiagnosis. In J-P Gabriel, C Lefevre, and P Picard, editors, *Stochastic processes in epidemic theory*, volume 86, pages 59–69. Springer-Verlag, Berlin, 1990.

[222] I Nå sell. On the quasi-stationary distribution of the Ross malaria model. *Mathematical Biosciences*, 107(2):187–207, 1991.

[223] Y Nagao and K Koelle. Decreases in dengue transmission may act to increase the incidence of dengue hemorrhagic fever. *Proceedings of the National Academy of Sciences of the United States of America*, 105(6):2238–2243, 2008.

[224] J A Najera. A critical review of the field application of a mathematical model of malaria eradication. *Bull World Health Organ*, 50(5):449–457, 1974.

[225] M Nakazawa, H Ohmae, A Ishii, and J Leafasia. Malaria infection and human behavioral factors: A stochastic model analysis for direct observation data in the Solomon Islands. *American Journal of Human Biology*, 10(6):781–789, 1998.

[226] J Nedelman. Inoculation and recovery rates in the malaria model of Dietz, Molineaux, and Thomas. *Mathematical Biosciences*, 69(2):209–233, 1984.

[227] J Nedelman. Estimation for a model of multiple malaria infections. *Biometrics*, 41(2):447–453, 1985.

[228] J Nedelman. Some New Thoughts About Some Old Malaria Models - Introductory Review. *Mathematical Biosciences*, 73(2):159–182, 1985.

[229] E A C Newton and P Reiter. A model of the transmission of dengue fever with an evaluation of the impact of ultra-low volume (ULV) insecticide applications on dengue epidemics. *American Journal of Tropical Medicine and Hygiene*, 47(6):709–720, 1992.

[230] G A Ngwa. Modelling the dynamics of endemic malaria in growing populations. *Discrete and Continuous Dynamical Systems-Series B*, 4(4):1173–1202, 2004.

[231] G A Ngwa and W S Shu. A mathematical model for endemic malaria with variable human and mosquito populations. *Mathematical and Computer Modelling*, 32(7-8):747–763, 2000.

[232] H Nishiura. Mathematical and statistical analyses of the spread of dengue. *Dengue Bulletin*, 30:51–67, 2006.

[233] H Nishiura. Duration of short-lived cross-protective immunity against a clinical attack of dengue: A preliminary estimate. *Dengue Bulletin*, 32:55–66, 2008.

[234] R A Norman, M S Chan, A Srividya, S P Pani, K D Ramaiah, P Vanamail, E Michael, P K Das, and D A Bundy. EPIFIL: the development of an age-structured model for describing the transmission dynamics and control of lymphatic filariasis. *Epidemiol Infect*, 124(3):529–541, 2000.

[235] N Nuraini, E Soewono, and K A Sidarto. Mathematical model of dengue disease transmission with severe DHF compartment. *Bulletin of the Malaysian Mathematical Sciences Society*, 30(2):143–157, 2007.

[236] L C Okell, C J Drakeley, T Bousema, C J M Whitty, and A C Ghani. Modelling the impact of artemisinin combination therapy and long-acting treatments on malaria transmission intensity. *Plos Medicine*, 5(11):1617–1628, 2008.

[237] F O Okumu, N J Govella, S J Moore, N Chitnis, and G F Killeen. Potential benefits, limitations and target product-profiles of odor-baited mosquito traps for malaria control in Africa. *PLoS One*, 5(7):e11573, 2010.

[238] G O Olaofe and K Olaofe. A simple model for tropical malaria epidemics. *Mathematical Biosciences*, 25:205–215, 1975.

[239] M Otero and H G Solari. Stochastic eco-epidemiological model of dengue disease transmission by Aedes aegypti mosquito. *Math Biosci*, 223(1):32–46, 2010.

[240] K Paaijmans, A Read, and M Thomas. Understanding the link between malaria risk and climate. *Proc Natl Acad Sci U S A*, 106(33):13844–13849, 2009.

[241] P E Parham and E Michael. Modelling climate change and malaria transmission. *Advances in experimental medicine and biology*, 673:184–199, 2010.

[242] Paul Edward Parham and Edwin Michael. Modeling the effects of weather and climate change on malaria transmission. *Environmental Health Perspectives*, 118(5):620–626, 2010.

[243] P R Parthasarathy. The effect of superinfection on the distribution of the infectious period–a continued fraction approximation. *IMA J Math Appl Med Biol*, 14(2):113–123, 1997.

[244] P Picard. A semi-hybrid model for malaria with limited superinfection. *Advances in Applied Probability*, 30(4):1027–1057, 1998.

[245] S T R Pinho, C P Ferreira, L Esteva, F R Barreto, V C Morato e Silva, and M G L Teixeira. Modelling the dynamics of dengue real epidemics. *Philosophical Transactions of the Royal Society A: Mathematical, Physical and Engineering Sciences*, 368(1933):5679–5693, 2010.

[246] A P Plaisier, S Subramanian, P K Das, W Souza, T Lapa, A F Furtado, C P B Van der Ploeg, J D F Habbema, and G J van Oortmarssen. The LYMFASIM simulation program for modeling lymphatic filariasis and its control. *Methods of Information in Medicine*, 37(1):97–108, 1998.

[247] P Pongsumpun, K Patanarapelert, M Sriprom, S Varamit, and I M Tang. Infection risk to travelers going to dengue fever endemic regions. *Southeast Asian J Trop Med Public Health*, 35(1):155–159, 2004.

[248] P Pongsumpun and I M Tang. A realistic age structured transmission model for dengue hemorrhagic fever in Thailand. *Southeast Asian J Trop Med Public Health*, 32(2):336–340, 2001.

[249] P Pongsumpun and I M Tang. Mathematical model for the transmission of Plasmodium vivax malaria. *International Journal of mathematical models and methods in applied statistics*, 1(3):117–121, 2007.

[250] W Pongtavornpinyo, S Yeung, I M Hastings, A M Dondorp, N P J Day, and N J White. Spread of anti-malarial drug resistance: Mathematical model with implications for ACT drug policies. *Malaria Journal*, 7:229, 2008.

[251] A Pugliese. Modelling multi-species parasite transmission. *Advances in experimental medicine and biology*, 673:32–50, 2010.

[252] J H Pull and B Grab. A simple epidemiological model for evaluating the malaria inoculation rate and the risk of infection in infants. *Bull World Health Organ*, 51(5):507–516, 1974.

[253] Z P Qiu. Dynamical behavior of a vector-host epidemic model with demographic structure. *Computers & Mathematics with Applications*, 56(12):3118–3129, 2008.

[254] J Radcliffe. The Initial Geographical Spread of Host-Vector and Carrier-Borne Epidemics. *Journal of Applied Probability*, 10(4):703–717, 1973.

[255] J Radcliffe. The Periodicity of Endemic Malaria. *Journal of Applied Probability*, 11(3):562–567, 1974.

[256] J Radcliffe. The Severity of a Viral Host-Vector Epidemic. *Journal of Applied Probability*, 13(4):791–794, 1976.

[257] N R Rao, O P Vig, and S N Agarwala. Transmission dynamics of malaria. Quantitative Studies - Part I: A stochastic model. *Bull Haffkine Inst*, 2(2):71–78, 1974.

[258] N R Rao, O P Vig, and S N Agarwala. Transmission dynamics of malaria. Quantitative Studies - Part II: Mathematical nature of expected happenings. *Bull Haffkine Inst*, 2(3):112–116, 1974.

[259] N R Rao, O P Vig, S N Agarwala, S S Sabnis, and V N Rao. Transmission dynamics of malaria. Part III Quantitiative studies: Computer simulation approach to measurement. *Journal of Communicable Diseases*, 8(4):246–254, 1976.

[260] Ana Rivero, Julien Vézilier, Mylène Weill, Andrew F Read, and Sylvain Gandon. Insecticide control of vector-borne diseases: when is insecticide resistance a problem? *PLoS Pathogens*, 6(8):e1001000, 2010.

[261] B Roche, J F Guegan, and F Bousquet. Multi-agent systems in epidemiology: A first step for computational biology in the study of vector-borne disease transmission. *BMC Bioinformatics*, 9:435, 2008.

[262] D J Rodriguez and L Torres-Sorando. Models of infectious diseases in spatially heterogeneous environments. *Bulletin of Mathematical Biology*, 63(3):547–571, 2001.

[263] D J Rogers and M J Packer. Vector-borne diseases, models, and global change. *Lancet*, 342(8882):1282–1284, 1993.

[264] A Ross, M Penny, N Maire, A Studer, I Carneiro, D Schellenberg, B Greenwood, M Tanner, and T Smith. Modelling the epidemiological impact of intermittent preventive treatment against malaria in infants. *PLoS One*, 3(7):e2661, 2008.

[265] P A Rossignol and A M Rossignol. Simulations of enhanced malaria transmission and host bias induced by modified vector blood location behaviour. *Parasitology*, 97 (Pt 3):363–372, 1988.

[266] S G Ruan, D M Xiao, and J C Beier. On the delayed Ross-Macdonald model for malaria transmission. *Bulletin of Mathematical Biology*, 70(4):1098–1114, 2008.

[267] F Rubel, K Brugger, M Hantel, S Chvala-Mannsberger, T Bakonyi, H Weissenbock, and N Nowotny. Explaining Usutu virus dynamics in Austria: model development and calibration. *Preventive Veterinary Medicine*, 85(3-4):166–186, 2008.

[268] D Ruiz, G Poveda, I D Velez, M L Quinones, G L Rua, L E Velasquez, and J S Zuluaga. Modelling entomological-climatic interactions of Plasmodium falciparum malaria transmission in two Colombian endemic-regions: contributions to a National Malaria Early Warning System. *Malaria Journal*, 5:66, 2006.

[269] L B L Santos, M C Costa, S T R Pinho, R F S Andrade, F R Barreto, M G Teixeira, and M L Barreto. Periodic forcing in a three-level cellular automata model for a vector-transmitted disease. *Physical Review E*, 80(1):Article Number: 016102, 2009.

[270] A Saul. Minimal efficacy requirements for malaria vaccines to significantly lower transmission in epidemic or seasonal malaria. *Acta Tropica*, 52(4):283–296, 1993.

[271] A Saul. Zooprophylaxis or zoopotentiation: The outcome of introducing animals on vector transmission is highly dependent on the mosquito mortality while searching. *Malaria Journal*, 2:32, 2003.

[272] A Saul. Efficacy model for mosquito stage transmission blocking vaccines for malaria. *Parasitology*, 135(13):1497–1506, 2008.

[273] H M Savage, D Aggarwal, C S Apperson, C R Katholi, E Gordon, H K Hassan, M Anderson, D Charnetzky, L McMillen, E A Unnasch, and T R Unnasch. Host choice and West Nile virus infection rates in blood-fed mosquitoes, including members of the Culex pipiens complex, from Memphis and Shelby County, Tennessee, 2002-2003. *Vector Borne Zoonotic Dis*, 7(3):365–386, 2007.

[274] I B Schwartz, L B Shaw, D A Cummings, L Billings, M McCrary, and D S Burke. Chaotic desynchronization of multistrain diseases. *Phys Rev E Stat Nonlin Soft Matter Phys*, 72(6 Pt 2):66201, 2005.

[275] T W Scott, R G McLean, D B Francy, and C S Card. A simulation model for the vector-host transmission system of a mosquito-borne avian virus, Turlock (Bunyaviridae). *Journal of Medical Entomology*, 20(6):625–640, 1983.

[276] J Shaman. Amplification due to spatial clustering in an individual-based model of mosquito-avian arbovirus transmission. *Transactions of the Royal Society of Tropical Medicine and Hygiene*, 101(5):469–483, 2007.

[277] L B Shaw, L Billings, and I B Schwartz. Using dimension reduction to improve outbreak predictability of multistrain diseases. *Journal of Mathematical Biology*, 55(1):1–19, 2007.

[278] H J Shi, Z S Duan, and G R Chen. An SIS model with infective medium on complex networks. *Physica a-Statistical Mechanics and Its Applications*, 387(8-9):2133–2144, 2008.

[279] A Slavova. Dynamics and traveling waves in CNN vector disease model. *IEEE Transactions on Circuits and Systems II-Express Briefs*, 53:1304–1307, 2005.

[280] D L Smith, J Dushoff, and F E McKenzie. The risk of a mosquito-borne infection in a heterogeneous environment. *PLoS Biol*, 2(11):e368, 2004.

[281] D L Smith, J Dushoff, R W Snow, and S I Hay. The entomological inoculation rate and Plasmodium falciparum infection in African children. *Nature*, 438(7067):492–495, 2005.

[282] D L Smith, C A Guerra, R W Snow, and S I Hay. Standardizing estimates of the Plasmodium falciparum parasite rate. *Malar J*, 6:131, 2007.

[283] D L Smith and S I Hay. Endemicity response timelines for Plasmodium falciparum elimination. *Malaria Journal*, 8:87, 2009.

[284] D L Smith, S I Hay, A M Noor, and R W Snow. Predicting changing malaria risk after expanded insecticide-treated net coverage in Africa. *Trends Parasitol*, 25(11):511–516, 2009.

[285] D L Smith, E Y Klein, F E McKenzie, and R Laxminarayan. Prospective strategies to delay the evolution of anti-malarial drug resistance: weighing the uncertainty. *Malar J*, 9:217, 2010.

[286] D L Smith and F E McKenzie. Statics and dynamics of malaria infection in Anopheles mosquitoes. *Malar J*, 3:13, 2004.

[287] D L Smith, F E McKenzie, R W Snow, and S I Hay. Revisiting the basic reproductive number for malaria and its implications for malaria control. *PLoS Biol*, 5(3):e42, 2007.

[288] T Smith, J L Hii, B Genton, I Muller, M Booth, N Gibson, A Narara, and M P Alpers. Associations of peak shifts in age–prevalence for human malarias with bednet coverage. *Trans R Soc Trop Med Hyg*, 95(1):1–6, 2001.

[289] T Smith, G Killeen, C Lengeler, and M Tanner. Relationships between the outcome of Plasmodium falciparum infection and the intensity of transmission in Africa. *Am J Trop Med Hyg*, 71(2 Suppl):80–86, 2004.

[290] T Smith, G F Killeen, N Maire, A Ross, L Molineaux, F Tediosi, G Hutton, J Utzinger, K Dietz, and M Tanner. Mathematical modeling of the impact of malaria vaccines on the clinical epidemiology and natural history of Plasmodium falciparum malaria: Overview. *American Journal of Tropical Medicine and Hygiene*, 75(2):1–10, 2006.

[291] T A Smith. Estimation of heterogeneity in malaria transmission by stochastic modelling of apparent deviations from mass action kinetics. *Malar J*, 7:12, 2008.

[292] T Sota. Variable host selection by mosquitoes and the dynamics of mosquito-borne diseases. *Japanese Journal of Sanitary Zoology*, 43(1):1–11, 1992.

[293] T Sota and M Mogi. Effectiveness of zooprophylaxis in malaria control: a theoretical inquiry, with a model for mosquito populations with two bloodmeal hosts. *Med Vet Entomol*, 3(4):337–345, 1989.

[294] C O A Sowunmi. Adler's deterministic model of vector-borne epidemics – a threshold theorem. *Mathematical Biosciences*, 35:47–54, 1977.

[295] M Sriprom, P Barbazan, and I M Tang. Destabilizing effect of the host immune status on the sequential transmission dynamic of the dengue virus infection. *Mathematical and Computer Modelling*, 45(9-10):1053–1066, 2007.

[296] S T Stoddard, A C Morrison, G M Vazquez-Prokopec, V Paz Soldan, T J Kochel, U Kitron, J P Elder, and T W Scott. The role of human movement in the transmission of vector-borne pathogens. *PLoS Negl Trop Dis*, 3(7):e481, 2009.

[297] W A Stolk, S Swaminathan, G J van Oortmarssen, P K Das, and J D F Habbema. Prospects for elimination of bancroftian filariasis by mass drug treatment in Pondicherry, India: A simulation study. *Journal of Infectious Diseases*, 188(9):1371–1381, 2003.

[298] C J Struchiner, M E Halloran, J M Robins, and A Spielman. The behaviour of common measures of association used to assess a vaccination programme under complex disease transmission patterns–a computer simulation study of malaria vaccines. *Int J Epidemiol*, 19(1):187–196, 1990.

[299] C J Struchiner, M E Halloran, and A Spielman. Modeling malaria vaccines. I: New uses for old ideas. *Mathematical Biosciences*, 94(1):87–113, 1989.

[300] A K Supriatna, E Soewono, and S A van Gils. A two-age-classes dengue transmission model. *Mathematical Biosciences*, 216(1):114–121, 2008.

[301] F C Tanser, B Sharp, and D le Sueur. Potential effect of climate change on malaria transmission in Africa. *Lancet*, 362(9398):1792–1798, 2003.

[302] A J Tatem, Y Qiu, D L Smith, O Sabot, A S Ali, and B Moonen. The use of mobile phone data for the estimation of the travel patterns and imported Plasmodium falciparum rates among Zanzibar residents. *Malar J*, 8:287, 2009.

[303] A J Tatem, D L Smith, P W Gething, C W Kabaria, R W Snow, and S I Hay. Ranking of elimination feasibility between malaria-endemic countries. *Lancet*, 376(9752):1579–1591, 2010.

[304] J M Tchuenche, C Chiyaka, D Chan, A Matthews, and G Mayer. A mathematical model for antimalarial drug resistance. *Math Med Biol*, 2010.

[305] Miranda I Teboh-Ewungkem, Chandra N Podder, and Abba B Gumel. Mathematical study of the role of gametocytes and an imperfect vaccine on malaria transmission dynamics. *Bull. Math. Biol.*, 72(1):63–93, 2010.

[306] F Tediosi, N Maire, M Penny, A Studer, and T A Smith. Simulation of the cost-effectiveness of malaria vaccines. *Malaria Journal*, 8:127, 2009.

[307] D M Thomas and B Urena. A model describing the evolution of West Nile-like encephalitis in New York City. *Mathematical and Computer Modelling*, 34:771–781, 2001.

[308] L Torres-Sorando and D J Rodriguez. Models of spatio-temporal dynamics in malaria . *Ecological Modelling*, 104(2-3):231–240, 1997.

[309] A Tran and M Raffy. On the dynamics of dengue epidemics from large-scale information. *Theoretical Population Biology*, 69(1):3–12, 2006.

[310] J Tumwiine, J Y T Mugisha, and L S Luboobi. A mathematical model for the dynamics of malaria in a human host and mosquito vector with temporary immunity. *Applied Mathematics and Computation*, 189(2):1953–1965, 2007.

[311] J Tumwiine, J Y T Mugisha, and L S Luboobi. Threshold and stability results for a malaria model in a population with protective intervention among high-risk groups. *Mathematical Modelling and Analysis*, 13(3):443–460, 2008.

[312] J Tumwiine, J Y T Mugisha, and L S Luboobi. A host-vector model for malaria with infective immigrants. *Journal of Mathematical Analysis and Applications*, 361(1):139–149, 2010.

[313] R Volz. Global asymptotic stability of a periodic solution to an epidemic model. *J Math Biol*, 15(3):319–338, 1982.

[314] H Wan and J A Cui. A model for the transmission of malaria. *Discrete and Continuous Dynamical Systems-Series B*, 11(2):479–496, 2009.

[315] H J Wearing and P Rohani. Ecological and immunological determinants of dengue epidemics. *Proceedings of the National Academy of Sciences of the United States of America*, 103(31):11802–11807, 2006.

[316] H M Wei, X Z Li, and M Martcheva. An epidemic model of a vector-borne disease with direct transmission and time delay. *Journal of Mathematical Analysis and Applications*, 342(2):895–908, 2008.

[317] M J Wonham, T De-Camino-Beck, and M A Lewis. An epidemiological model for West Nile virus: Invasion analysis and control applications. *Proceedings of the Royal Society of London Series B-Biological Sciences*, 271(1538):501–507, 2004.

[318] Marjorie J Wonham, Mark A Lewis, Joanna Renclawowicz, and P van den Driessche. Transmission assumptions generate conflicting predictions in host-vector disease models: a case study in West Nile virus. *Ecology Letters*, 9(6):706–725, 2006.

[319] M E Woolhouse, C Dye, J F Etard, T Smith, J D Charlwood, G P Garnett, P Hagan, J L Hii, P D Ndhlovu, R J Quinnell, C H Watts, S K Chandiwana, and R M Anderson. Heterogeneities in the transmission of infectious agents: implications for the design of control programs. *Proc Natl Acad Sci U S A*, 94(1):338–342, 1997.

[320] E Worrall, S J Connor, and M C Thomson. A model to simulate the impact of timing, coverage and transmission intensity on the effectiveness of indoor residual spraying (IRS) for malaria control. *Tropical Medicine & International Health*, 12(1):75–88, 2007.

[321] A P P Wyse, L Bevilacqua, and M Rafikou. Simulating malaria model for different treatment intensities in a variable environment. *Ecological Modelling*, 206(3-4):322–330, 2007.

[322] H M Yang. Malaria transmission model for different levels of acquired immunity and temperature-dependent parameters (vector). *Rev Saude Publica*, 34(3):223–231, 2000.

[323] H M Yang and C P Ferreira. Assessing the effects of vector control on dengue transmission. *Applied Mathematics and Computation*, 198(1):401–413, 2008.

[324] H M Yang, M L G Macoris, K C Galvani, M T M Andrighetti, and D M V Wanderley. Assessing the effects of temperature on dengue transmission. *Epidemiology and Infection*, 137(8):1179–1187, 2009.

[325] Y Ye, R Sauerborn, S Seraphin, and M Hoshen. Using modelling to assess the risk of malarial infection during the dry season, on a local scale in an endemic area of rural Burkina Faso. *Annals of Tropical Medicine and Parasitology*, 101(5):375–389, 2007.
