# Supplementary material for: A systematic review of mathematical models of mosquito-borne pathogen transmission: 1970–2010
Source: J R Soc Interface. 2013 Apr 6;10(81):20120921. doi: 10.1098/rsif.2012.0921 (PMC3627099; doi:10.1098/rsif.2012.0921)
Supplement: Supporting Information 2 [file rsif20120921supp2.pdf]

Determination of Model Inclusion using the Questionnaire

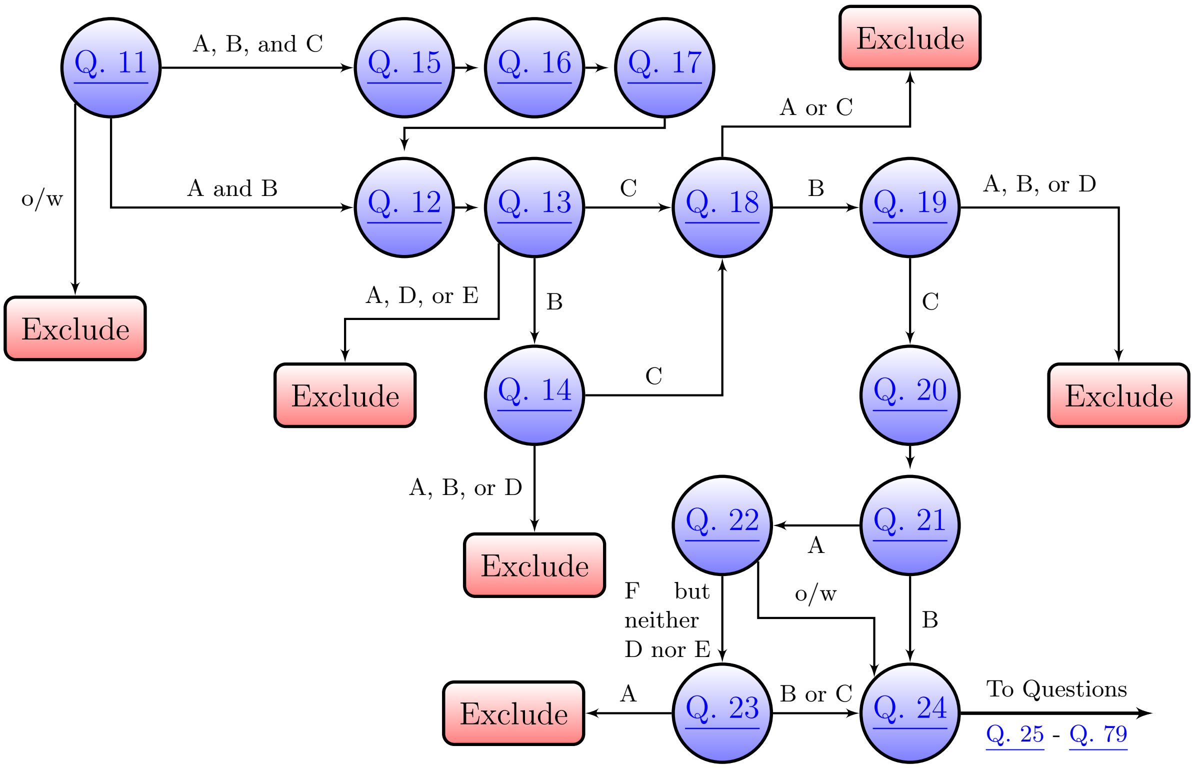

Figure S2.1. Q. 11-Q. 24 are used to exclude models from the inventory that do not meet our basic inclusion criteria. Models that meet all of these criteria then proceed to Q. 25 and others thereafter as appropriate.

Logic Flow of the Questionnaire

All models which were not eliminated by Q. 24 must then answer

Basic Questions: Q. 25 - Q. 32.
Mixing, Heterogeneous or Preferential Biting: Q. 72, Q. 74 - Q. 76.
Analysis : Q. 79.

The basic questions determine which additional questions are appropriate.

For Q. 25, an answer of
- (A) may then open Q. 45 (if the answer to Q. 26 was (E)),
- (B) will then open Q. 36, Q. 38, and Q. 39,
- (C) will then open Q. 37 - Q. 39.

For Q. 26, an answer of
- (B) will then open Q. 40. It may open Q. 41 (if the answer to Q. 28 was (B)),
- (C) will then open Q. 42 and Q. 43. It may open Q. 44 (if the answer to Q. 28 was (B)),
- (D) will then open Q. 40. It may open Q. 41 (if the answer to Q. 28 was (B)),
- (E) will then open Q. 46 - Q. 48, Q. 50, Q. 51, Q. 53, and Q. 55. It may open Q. 45 (if the answer to Q. 25 was (A)) and Q. 49 (if the answer to Q. 28 was (B)).

For Q. 27, an answer of
- (C) will then open Q. 58, Q. 59, and Q. 63 - Q. 68. It may open Q. 62 (if the answer to Q. 28 was (B)).

For Q. 28, an answer of
- (B) may then open Q. 41 (if the answer to Q. 26 was (? Back)) or Q. 44 (if the answer to Q. 26 was (?)) or Q. 49 (if the answer to Q. 26 was (E)) and may open Q. 62 (if the answer to Q. 27 was (C)).

For Q. 29, an answer of
- (C) will then open Q. 57 and Q. 58,
- (D) will then open Q. 57 and Q. 58.

For Q. 30, an answer of
- (B) may then open Q. 69 (if the answer to Q. 27 was (C) and then the answer to Q. 68 was (A)),
- (C) may then open Q. 69 (if the answer to Q. 27 was (C) and then the answer to Q. 68 was (A)).

For Q. 31, an answer of
- (C) will then open Q. 70 and Q. 71,
- (D) will then open Q. 70 and Q. 71.

For Q. 32, an answer of
- (A) will then open Q. 77 and Q. 78.

Within the following questions, certain responses result in follow-up questions. For all below example, for brevity, the conditions that lead to the situation (i.e. the specific necessary responses to Q. 25 - Q. 32) are not included.

If Q. 51 is asked and the answer is (C), (D), (E), (G) or (H), Q. 52 is opened.
If Q. 53 is asked and the answer is (B) or (C), Q. 54 is opened.
If Q. 59 is asked and the answer is (A), Q. 60 and Q. 61 are opened.
If Q. 68 is asked, the answer is (A) and the answer to Q. 30 was (B) or (C), then Q. 69 is opened.
If Q. 72 is asked and the answer is (B), Q. 73 is opened.

Questions and Possible Answers in the Questionnaire

1. Exclusion criteria

Question 11 - Which of the following statements best describes this paper? Back

(A) It is about mosquitoes or mosquito-transmitted pathogens.
(B) It contains a formula or equation, or one is implied or implicitly used.
(C) It contains estimates of transmission by some metric, or some data that can be used to estimate parameters in a mosquito-borne pathogen transmission model
(D) None of the above

Question 12 - Many studies of transmission are difficult to classify in relation to the Ross-Macdonald model, even if they were motivated by the Ross-Macdonald theory. This question is designed to filter out some particular kinds of studies that are likely to be difficult to classify. Please select one of the following if it describes this paper: Back

(A) It estimates R_0 from data describing the increase in the number of cases at the beginning of an epidemic.
(B) It describes or analyzes data from a longitudinal study or from cross-sectional prevalence or sero-prevalence data, or it develops theory explaining how to do so.
(C) It develops and presents a map of the geographical range of a pathogen, the risk of infection, or the potential for transmission.
(D) It develops an early warning system or presents a method for forecasting disease.
(E) It discusses or evaluates metrics of transmission.
(F) It develops a model for mosquito search or movement.
(G) It develops a model for vertebrate host movement.
(H) It is an economic analysis (e.g., cost-benefit analysis).
(I) It analyzes a time series.
(J) None of the above.
(K) Other.

Question 13 - Is the model or formula based on or motivated by an "a priori" or an "a posteriori" approach? Or in other words, does this take a mechanistic or process-based approach or an empirical approach to transmission? Back

(A) It was identified in the previous question, whether or not it is a priori or a posteriori.
(B) It is clearly a priori: the model or formula itself is based on assumptions about the process or mechanisms (e.g., Ross' or Macdonald's transmission models).
(C) It is not clearly either one, but the intent of the analysis itself was to understand, quantify, or describe a mechanism or some part of the process of transmission (e.g., estimating the force of infection from cross-sectional prevalence or seroprevalence data).
(D) It is clearly a posteriori or empirical: the model is used to describe patterns in data. The model itself could describe many different kinds of data (e.g., regression analysis).
(E) Other.

Question 14 - Based on your answers so far, the paper may use models or concepts in a very limited way. Please select any of the following that apply. Back

(A) There is no transmission model or part of it.
(B) There is some part of a transmission model, but it is very simple, and probably not worth comparing its structure to other transmission models.
(C) There is a transmission model or some part of a transmission model that is worth evaluating in relation to other transmission models.
(D) Other.

Question 15 - Which parameters have been (or could be) estimated by the data presented in this paper? Back

(A) Mosquito population density, abundance, or other measures of mosquito occurrence.
(B) Mosquito survival.
(C) Blood feeding rates.
(D) Host search or selection by mosquitoes.

(E) Nectar feeding.
(F) Other aspects of the feeding cycle.
(G) Heterogeneous biting.
(H) Duration of the pathogen latent period in mosquitoes.
(I) Vector competence or transmission efficiency.
(J) Risk factors for clinical disease or severe outcomes.
(K) Duration of the pathogen latent period in vertebrate hosts.
(L) Duration of the infectious period in vertebrate hosts.
(M) Mosquito movement.
(N) Vertebrate host movement.
(O) None of these.
(P) Other.

Question 16 - Which transmission metrics have been (or could be) estimated by data presented in this paper? Back

(A) The prevalence of infection in the vertebrate host (e.g. malaria parasite rates).
(B) Seroconversion rates (e.g., using age-stratified seroprevalence to an immune marker of infection).
(C) Clinical incidence of infection (e.g., dengue case data).
(D) Mortality data.
(E) The force of infection.
(F) Biting rates on humans (i.e., HBR) or other vertebrate hosts.
(G) The entomological inoculation rate.
(H) The proportion of infected or infectious mosquitoes.
(I) The infectious reservoir of pathogens in vertebrate hosts.
(J) Vectorial capacity (or a similar index).
(K) R_0 or some other reproductive number (e.g., R_c).
(L) The pathogen importation rate.
(M) Spatial clustering / spatial scale of transmission.
(N) Pathogen resistance to drugs.
(O) Mosquito resistance in insecticides.
(P) Inherited blood disorders related to mosquito-borne pathogen transmission.
(Q) Biological cost of drug resistance.
(R) Biological cost of insecticide.
(S) Coverage levels of some intervention.
(T) None.
(U) Other.

Question 17 - Which of the following describe the data included in this paper? Back

(A) The data are being published for the first time in peer review.
(B) The data have already been published.
(C) This is a compilation of data aggregated from multiple sources.
(D) None of the above.
(E) Other.

Question 18 - Which of the following best describes the manuscript? Back

(A) It describes only mosquitoes or mosquito populations or their control. The analysis itself does not consider a pathogen of vertebrate hosts.
(B) It describes a pathogen of vertebrate hosts.
(C) Other.

Question 19 - Which one of the following statements best describes this paper? Back

(A) It describes some aspects of pathogen infection dynamics, but only events taking place inside the skin of the vertebrate host.
(B) It describes some aspects of pathogen infection dynamics, but only events taking place inside the skin of the mosquito.

(C) It describes other or broader aspects of pathogen transmission.
(D) None of the above.
(E) Other.

Question 20 - Does this paper use, extend, or critique one of these recognizable bodies of work? Back

(A) The Ross-Macdonald theory of transmission.
(B) The Garki model.
(C) The Strain Theory of Malaria Transmission.
(D) The McKenzie model.
(E) DENSim / CIMSim.
(F) EPIFIL.
(G) LYMFASIM.
(H) The Swiss Tropical Institute Malaria Model.
(I) HYDREMATS.
(J) The Imperial College Malaria Model.
(K) The Malaria Atlas Project.
(L) None of the above.
(M) Other.

Question 21 - Is this paper concerned with population genetics or evolution? Back

(A) Yes.
(B) No.

Question 22 - What aspects of evolution were considered? Please choose all that apply. Back

(A) Evolving mosquito populations.
(B) Evolving pathogen populations.
(C) Evolving vertebrate host populations.
(D) Pathogen transmission dynamics (e.g., state variables describing infection status).
(E) Population dynamics (e.g., state variables describing birth-death processes).
(F) Gene or genotype frequencies.
(G) None of the above.
(H) Other.

Question 23 - Your answers suggest this is a population genetic model; i.e., it tracks gene frequencies. Is this actually a population genetic model, or is it an evolutionary model? Back

(A) It is a population genetic model: the state variables are gene frequencies.
(B) It is an evolutionary model that considers the dynamics of competing "types."
(C) Other.

Question 24 - Which pathogen or pathogens are discussed in this paper? Back

(A) A generic pathogen.
(B) Malaria parasites.
(C) Filarial parasites.
(D) Dengue Fever virus.
(E) West Nile Fever virus.
(F) Rift Valley Fever virus.
(G) Yellow Fever virus.
(H) A generic arbovirus.
(I) None of the above.
(J) Other.

2. Basic Questions.

Question 25 - Which one of the following best describes the way aquatic populations were modeled? Back

(A) Not at all.
(B) Implicitly: The emergence rate of adult mosquitoes was described by a parameter or function but no state variable.
(C) Explicitly: Aquatic populations were modeled with at least one state variable.
(D) Other.

Question 26 - Which one of the following best describes the way adult mosquito population and infection dynamics were modeled? Back

(A) Not at all.
(B) Implicitly: There was no state variable describing mosquitoes, but there was a parameter (e.g., h) or function describing vertebrate host exposure to pathogens over time (possibly fitted to data).
(C) Implicitly: There was no state variable describing mosquitoes, but transmission was modeled dynamically. The language and notation was similar to that used for directly transmitted pathogens (e.g., βI).
(D) Implicitly: There was no state variable describing mosquitoes, but transmission was modeled dynamically with a function that accepted two kinds of arguments (e.g., $h = cVX$): 1) terms associated with the Ross-Macdonald model or mosquitoes (i.e., vectorial capacity V or a similar term); and 2) terms describing the infectious reservoir in vertebrate hosts (e.g., cX)
(E) Explicitly: Adult mosquito populations were modeled with at least one state variable.
(F) Other.

Question 27 - Which one of the following best describes the way infections in vertebrate hosts were modeled? Back

(A) Not at all.
(B) Implicitly: mosquito exposure to the infectious reservoir in vertebrate hosts was described by a parameter.
(C) Explicitly: pathogen infection dynamics in vertebrate hosts were modeled with at least one state variable.
(D) Other.

Question 28 - How many spatial locations were included in or implied by the model? Back

(A) One place with no emigration or emigration; or location was undefined or vaguely defined (i.e., the model was spaceless).
(B) There was more than one location or place; or the model included terms describing immigration or emigration of mosquitoes or vertebrate hosts.
(C) Other.

Question 29 - How many mosquito taxa, genotypes or phenotypes were considered? Back

(A) 0.
(B) 1.
(C) 2.
(D) More than 2.

Question 30 - How many pathogen taxa, genotypes, or phenotypes were considered? Back

(A) 1.
(B) 2.
(C) More than 2.

Question 31 - How many vertebrate taxa, genotypes, or phenotypes were considered? Back

(A) 0.
(B) 1.
(C) 2.
(D) More than 2.

Question 32 - Did the model consider any type of control? Back

(A) Yes.
(B) No.

3. Spatial Dynamics

Question 33 - How many spatial locations were included in or implied by the model? Back

- (A) One patch (with migration).
- (B) Two "patches" or locations.
- (C) Three or more "patches" or locations.
- (D) Space was continuous (e.g., in PDEs or in an IBM).
- (E) The model described a connectivity network among individual hosts.
- (F) Other.

Question 34 - How many spatial locations were included in or implied by the model? Back

- (A) In an array.
- (B) In a grid or lattice.
- (C) A network of interconnected patches or points.
- (D) Other.

Question 35 - What moves? Back

- (A) Nothing.
- (B) Vertebrate hosts move.
- (C) Mosquito hosts move.
- (D) Both vertebrate hosts and mosquito hosts move.

4. Aquatic Mosquito Ecology

Question 36 - Which of the following best describes the parameter or function describing adult mosquito emergence? (If there are several locations, answer the question for a typical patch or location.) Back

(A) A constant.
(B) A sinusoidal function, arbitrarily defined.
(C) A sinusoidal function, fitted in some way to local data.
(D) Other.

Question 37 - What factors were included in the model of larval populations? (If there were multiple patches / locations, please answer the question for a typical location.) Back

(A) Larval dynamics were density independent.
(B) Logistic-style carrying capacity.
(C) Power-law.
(D) Resource-based competition.
(E) Predation.
(F) None of the above.
(G) Other.

Question 38 - What other features of aquatic populations were modeled? Back

(A) Aquatic habitat was created and destroyed by rainfall / desiccation.
(B) Aquatic populations were lost through flushing / overfilling of habitat.
(C) Development of immature stages was temperature dependent.
(D) There was larval competition with other mosquitoes.
(E) Egg populations were explicitly modeled.
(F) Larval age.
(G) Larval stages (i.e., instars).
(H) Dormancy / long-term persistence of the eggs was possible.
(I) None of the above.
(J) Other.

Question 39 - How did aquatic ecology differ across space? Back

(A) The same rates or functions applied everywhere.
(B) Different rates or functions were used to illustrate a general principle.
(C) Different rates or functions were used, based on data.
(D) Other.

5. Quasi-Direct Transmission

Question 40 - Which one of the following best describes the contact parameter? (If this was a spatial model, answer the question for a typical location.) Back

(A) Constant.
(B) Constant and fitted to local data.
(C) Seasonally forced, with a sinusoidal function.
(D) Seasonally forced, with a sinusoidal function and fitted to local data.
(E) Realistically forced.
(F) Other.

Question 41 - How did contact rates vary across space? Back

(A) Spatially homogeneous.
(B) Spatially heterogeneous, chosen strategically.
(C) Spatially heterogeneous, based on population density.
(D) Spatially heterogeneous, based on data.
(E) Other.

6. Minimized Mosquito Infection Dynamics

Question 42 - Was the "minimal mosquito" assumption implemented with a delay for the explicit incubation period? Back

(A) Yes.
(B) No.

Question 43 - Which one of the following best describes the way the minimal mosquito assumption was implemented? Back

(A) Vectorial capacity (or EIR or R_0) was constant.
(B) Vectorial capacity (or EIR or R_0) was constant and fitted to local data.
(C) Vectorial capacity (or EIR or R_0) was seasonally forced, with a sinusoidal function.
(D) Vectorial capacity (or EIR or R_0) was seasonally forced, based on a sinusoidal function fitted mosquito population data.
(E) Vectorial capacity (or EIR or R_0) was realistically forced using mosquito population data.
(F) Other.

Question 44 - How did vectorial capacity (or EIR or R_0) vary across space? Back

(A) Spatially homogeneous.
(B) Spatially heterogeneous, chosen strategically.
(C) Spatially heterogeneous, based on data.
(D) Other.

7. Adult Mosquito Demography and Feeding Behavior

Question 45 - How was adult mosquito density modeled? Back

(A) Constant.
(B) A sinusoidal function.
(C) Based on a pattern derived from data.
(D) None of the above.
(E) Other.

Question 46 - What assumptions were made about adult mosquito mortality in the absence of control? Back

(A) Constant per-capita mortality (i.e., exponentially or geometrically distributed lifespans).
(B) With temperature-dependent mortality.
(C) With humidty-dependent mortality.
(D) With senescence (i.e., age-dependent mortality).
(E) With frailty (i.e., different lifespans for different mosquito types).
(F) Mosquito mortality was not part of this model.
(G) None of the above.
(H) Other.

Question 47 - What assumptions were made about mosquito blood-feeding rates (i.e., the interval between two consecutive bloodmeals) in the absence of control? Back

(A) Blood feeding occurred at a constant per-capita rate.
(B) Blood-feeding rates depended explicitly on the outcome of searching.
(C) Blood-feeding rates varied with temperature.
(D) Blood-feeding rates varied with the availability of vertebrate hosts or their behavior.
(E) Blood-feeding rates varied were altered by control.
(F) Blood feeding was not considered in this model.
(G) None of the above.
(H) Other.

Question 48 - What assumption was made about the proportion of bloodmeals taken on pathogen's host(s)? Back

(A) Feeding on other vertebrate hosts was included only implicticly or not at all.
(B) A constant fraction of mosquitoes fed on each vertebrate host species.
(C) Feeding on vertebrate hosts was modeled with a frequency dependent function.
(D) Host selection was modeled as the outcome of a search algorithm (e.g., in an IBM).
(E) Other.

Question 49 - What aspects of mosquito behavior were spatially heterogeneous? Back

(A) Adult mosquito survival.
(B) Pathogen development rates, based on temperature.
(C) Feeding rates.
(D) The availability of vertebrate hosts.
(E) None.
(F) Other.

Question 50 - Which of these other aspects of adult mosquitoes were included in the model? Back

(A) Egg laying.
(B) Mating.
(C) Resting.
(D) Nectar feeding.
(E) Male mosquito populations.
(F) None of the above.
(G) Other.

8. Pathogen Infection Dynamics in Mosquitoes

Question 51 - Which one of the following best describes the way infections in mosquitoes were modeled? Back

(A) Not at all.
(B) A function or formula, but no state variable or equation.
(C) ODE: Some kind of a compartment model.
(D) With a stochastic simulation that was based on some kind of compartment model.
(E) A delay differential equation, and based on some kind of compartment model.
(F) PDE: One or more independent variable described the infection.
(G) PDE: Independent variables described host age or space, but the infection dynamics were based on a compartment model.
(H) With difference equations (i.e., a fixed time step) that were based on some kind of compartment model.
(I) With some kind of difference equation.
(J) With an individual-based computer simulation (other than the ones described above).
(K) Other.

Question 52 - Which compartments were considered? Back

(A) S: With a class for uninfected.
(B) E: One "exposed" or "infected but not infectious" class.
(C) E_n: With several exposed classes to model a "realistic incubation period."
(D) I: Infected and infectious.
(E) I_n: With mosquito superinfection.
(F) R: With a "recovered and immune" class.
(G) Other.

Question 53 - Did the model consider pathogen latency in mosquitoes? Back

(A) Not at all.
(B) Implicitly: mortality during the latent period was considered, but not with a state variable.
(C) Explicitly: the latent period was part of the model structure.
(D) Other.

Question 54 - Was the pathogen development rate in the mosquito temperature dependent? Back

(A) Yes.
(B) No.

Question 55 - Which of these other aspects of transmission by mosquitoes were included in the model? Back

(A) Vertical transmission.
(B) Recovery from infection / waning infection.
(C) A cost of infection (i.e., virulence).
(D) Mosquito superinfection.
(E) None of the above.
(F) Other.

Question 56 - How did the vector species or types differ? Back

(A) Population density.
(B) Lifespan.
(C) Feeding rates.
(D) Host feeding preferences.
(E) Resting behavior.
(F) Vertical transmission.
(G) Movement patterns.
(H) Seasonal patterns.
(I) Infectivity: their susceptibility to infection.

(J) Infectivity: the efficiency of transmission from an infectious mosquito.
(K) Insecticide resistance.
(L) None of the above.
(M) Other.

Question 57 - Did the paper call the different mosquito variants “species” or were they different types (e.g., genotypes / phenotypes) of the same species? Back

(A) Different species.
(B) Different types of the same species.
(C) There was no distinction made.
(D) Other.

9. Vertebrate Host Population Dynamics

Question 58 - Which of the following did the model consider? Back

(A) Vertebrate host nutritional status.
(B) Vertebrate host genotype.
(C) Vertebrate host defensive behavior (i.e., swatting).
(D) Co-infection with other pathogens (e.g., HIV, TB).
(E) Vertebrate host age.
(F) None of the above.
(G) Other.

Question 59 - Did the model include any vital dynamics for the vertebrate host populations? Back

(A) Yes.
(B) No.

Question 60 - How were birth rates modeled? Back

(A) Population birth rate was constant.
(B) Population birth rates were described by a time-dependent parameter or function.
(C) Population birth rates were constant, per-capita.
(D) Population change was described by the Lotka-Volterra equation.
(E) There was age-structure with age-specific birth rates.
(F) Birth rates were not modeled.
(G) Other.

Question 61 - How were vertebrate host deaths modeled in the absence of disease? Back

(A) Not at all.
(B) Constant per-capita death rates.
(C) Death rate dependent on nutritional status or coinfection.
(D) Other.

Question 62 - What aspects of the vertebrate host populations were spatially heterogeneous? Back

(A) Heterogeneous population distributions.
(B) Heterogeneous genetic composition.
(C) Heterogeneous behavior.
(D) Heterogeneous economic status.
(E) Heterogeneous cultural practices.
(F) Heterogeneous household types.
(G) None of the above.
(H) Other.

10. Vertebrate Host Infections

Question 63 - Which one of the following best describes the way infections in vertebrate hosts were modeled? Back

(A) ODE: Some kind of a compartment model with ordinary differential equations.
(B) With a stochastic simulation that was based on some kind of compartment model.
(C) A delay differential equation, and based on some kind of compartment model.
(D) PDE: One or more independent variable described the infection.
(E) PDE: Independent variables described host age or space, but the infection dynamics were based on a compartment model.
(F) DE: With difference equations (i.e., discrete time step) that were based on some kind of compartment model.
(G) With some other kind of difference equation.
(H) With an individual-based computer simulation (other than the ones described above).
(I) Other.

Question 64 - If the type of compartment model is in this list, please select it. Otherwise, select "Other compartment model." Back

(A) SIS.
(B) $SEIS$.
(C) SIR.
(D) $SIRS$.
(E) $SEIR$.
(F) $SEIRS$.
(G) $SIS - SIS$ (an SIS model with two immune stages).
(H) $SEIS - SEIS$ (an $SEIS$ model with two immune stages).
(I) $SEIN - SEN$ (as in the Garki model: N means infected but not infectious, the second set of compartments is partially immune).
(J) Other compartment model (not exactly in this list).

Question 65 - What compartments were considered? Back

(A) M - born with maternal antibodies.
(B) S - susceptible to infection.
(C) E - exposed but not yet infectious.
(D) E_n - exposed but not yet infectious with a "realistic" incubation period.
(E) I - infected and infectious.
(F) I_n - infected and infectious with several infection stages.
(G) R - recovered and immune to further infection.
(H) D - dormant infection (e.g., hypnozoite infections).
(I) G - infected and infectious only with non-self-propagating forms (e.g., gametocytes).
(J) S^* - susceptible to infection but with partial immunity (e.g., with stage-structured immunity).
(K) E^* - exposed but not yet infectious but with partial immunity (e.g., with stage-structured immunity).
(L) I^* - infected and infectious but with partial immunity (e.g., with stage-structured immunity).
(M) D^* - dormant infection (e.g., hypnozoite infections) but with partial immunity (e.g., with stage-structured immunity).
(N) G^* - infected and infectious only with non-self-propagating forms (e.g., gametocytes) but with partial immunity (e.g., with stage-structured immunity).
(O) Other.

Question 66 - Was it possible for immunity to wane? Back

(A) Yes, in uninfected immune or partially immune hosts.
(B) Yes, but waning was slowed down by re-exposure.
(C) No.
(D) Not relevant.
(E) Other.

Question 67 - What clinical outcomes were described? Back

(A) Symptomatic and asymptomatic infections.
(B) Mild versus severe symptoms.
(C) Disease-induced mortality.
(D) Chronic sequelae.
(E) Low birth weights in children born to infected mothers.
(F) None of the above.
(G) Other.

Question 68 - Was it possible for a vertebrate host to be “superinfected” or “coinfected.” In other words, could a vertebrate host carry multiplie infections with the same or different pathogen “types”? Back

(A) Yes.
(B) No.

Question 69 - How was “superinfection” or “coinfection” modeled? Back

(A) With a function describing waiting time to clear.
(B) With a Markov-chain model on the multiplicity of infection.
(C) Infection with two or more types was represented with a state variable.
(D) With an individual-based model.
(E) Other.

Question 70 - What were the important differences among the vertebrate host species or types? Back

(A) Population density.
(B) Population dynamics.
(C) Movement patterns.
(D) Their attractiveness to mosquitoes.
(E) The intensity of infection and infectiousness to mosquitoes.
(F) The duration of the infectious period.
(G) The development of immunity.
(H) Some species were sinks for the pathogen.
(I) None of the above.
(J) Other.

Question 71 - Did the paper call the different host variants “species” or different “genotypes / phenotypes” of the same species? Back

(A) Different species.
(B) Different types of the same species.
(C) The paper was deliberately vague.
(D) Other.

11. Mixing, Heterogeneous or Preferential Biting

Question 72 - How were bloodmeals distributed among vertebrate hosts? Back

(A) Homogeneously.
(B) Heterogeneously.
(C) Not relevant.
(D) Other.

Question 73 - Which of the following were associated with heterogeneous biting? Back

(A) Host age.
(B) Host body size.
(C) Host behavior of any sort.
(D) Host infection status.
(E) Biting rates were described by a continuous probability distribution.
(F) Biting rates were divided into a finite number of classes.
(G) None of the above.
(H) Other.

Question 74 - Which one of the following describes mixing? Back

(A) Well mixed.
(B) A specific contact network was specified or described.
(C) Mixing was the outcome of an individual-based model.
(D) Mixing was not part of this model.
(E) None of the above.
(F) Other.

Question 75 - Which of the following parameters or terms describe transmission from the infectious mosquito to its vertebrate host? Back

(A) It was implicitly set to 1.
(B) It was represented by a constant parameter b.
(C) The parameter b differed in some way with partial immunity.
(D) The parameter b differed in some way with transmission intensity.
(E) The parameter b differed with the intensity of infection in mosquitoes.
(F) None of the above.
(G) Other.

Question 76 - Which of the following parameters or terms describes transmission from the infectious host to the mosquito? Back

(A) It was implicitly set to 1.
(B) It was represented by a constant c.
(C) It varied with the level of immunity in the vertebrate host.
(D) It varied with the age of infection.
(E) It varied with pathogen loads.
(F) None of the above.
(G) Other.

12. Control

Question 77 - What types of control were considered? Back

(A) None.
(B) Introduction of genetically modified mosquito populations replacement (e.g., refractory mosquitoes).
(C) Control of mosquito populations with sterile male release.
(D) Control of aquatic mosquito populations with larvicides.
(E) Biological control of mosquito populations.
(F) Introduction of genetically modified mosquito populations for population supression.
(G) Control of adult mosquito populations with indoor spraying.
(H) Control of adult mosquito populations with mass spraying.
(I) Control of adult populations / transmission with bednets (insecticide treated or not).
(J) Control of disease or transmission with a vaccine.
(K) Control of disease or transmission by treating clinical cases with drugs.
(L) Control of disease or transmission with mass screening and treating or mass drug administration.
(M) Control of transmission by culling the vertebrate host population.
(N) Control with zooprophylaxis.
(O) Other.

Question 78 - Which of the following aspects of control did the model consider? Back

(A) Costs.
(B) Operational constraints.
(C) Targeted application.
(D) Uneven or heterogeneous distribution.
(E) How well the intervention works.
(F) Other.

13. Description of the Analysis

Question 79 - What aspects of an epidemic were analyzed? Back

(A) Thresholds.
(B) The evolution of an epidemic.
(C) Stochastic invasion of a pathogen.
(D) Controlling an outbreak.
(E) Modeling control effect sizes or responses.
(F) Seasonality and its consequences.
(G) Population cycles.
(H) Steady states / endemicity.
(I) Local persistence of the pathogen.
(J) Stochastic fadeout.
(K) Low intensity or declining transmission.
(L) Control response timelines.
(M) Spatial persistence.
(N) Mapping potential transmission.
(O) Pathogen evolution.
(P) Host evolution.
(Q) Mosquito evolution.
(R) Analysis of a time series.
(S) Economics or bioeconomics.
(T) Mosquito behavior.
(U) Vertebrate host behavior.
(V) None of the above.
(W) Other.
