# Supplementary material for: A systematic review of mathematical models of mosquito-borne pathogen transmission: 1970–2010
Source: J R Soc Interface. 2013 Apr 6;10(81):20120921. doi: 10.1098/rsif.2012.0921 (PMC3627099; doi:10.1098/rsif.2012.0921)
Supplement: Supporting Information 4 [file rsif20120921supp4.pdf]

Table S1. Number of models per publication.

	Malaria	Dengue	WNV	Filariasis	Other	Total
1	89% (171)	94% (68)	68% (15)	93% (13)	87% (47)	88% (286)
2	5% (10)	4% (3)	23% (5)	0% (0)	9% (5)	7% (23)
3	4% (7)	1% (1)	9% (2)	7% (1)	2% (1)	3% (11)
4	1% (2)	0% (0)	0% (0)	0% (0)	0% (0)	1% (2)
5	1% (2)	0% (0)	0% (0)	0% (0)	2% (1)	1% (3)
Total publications	192	72	22	14	54	325
Total models	230	77	31	16	65	388

1. Exclusion

Table S2. Question 11 - Basic Exclusion

	Malaria	Dengue	WNV	Filariasis	Other	Total
Is about mosquitoes or mosquito transmitted pathogens and contains a formula or equation	53% (123)	55% (42)	58% (18)	19% (3)	66% (43)	56% (218)
Additionally, estimates transmission or provides data that can be used to estimated transmission	47% (107)	45% (35)	42% (13)	81% (13)	34% (22)	44% (170)
Total	230	77	31	16	65	388

Table S3. Question 12 - Paper description

	Malaria	Dengue	WNV	Filariasis	Other	Total
It analyzes a time series.	1% (3)	6% (5)	0% (0)	0% (0)	0% (0)	2% (8)
It describes or analyzes data from a longitudinal study or from cross-sectional prevalence or sero-prevalence data, or it develops theory explaining how to do so.	10% (22)	8% (6)	0% (0)	31% (5)	0% (0)	9% (33)
It develops a model for mosquito search or movement.	3% (8)	1% (1)	3% (1)	0% (0)	0% (0)	3% (10)
It develops a model for vertebrate host movement.	0% (1)	1% (1)	0% (0)	0% (0)	0% (0)	1% (2)
It develops an early warning system or presents a method for forecasting disease.	3% (6)	0% (0)	0% (0)	6% (1)	2% (1)	2% (8)
It develops and presents a map of the geographical range of a pathogen, the risk of infection, or the potential for transmission.	5% (12)	0% (0)	3% (1)	0% (0)	0% (0)	3% (13)
It discusses or evaluates metrics of transmission.	32% (73)	22% (17)	52% (16)	25% (4)	26% (17)	30% (118)
It estimates Ro from data describing the increase in the number of cases at the beginning of an epidemic.	1% (2)	8% (6)	0% (0)	0% (0)	2% (1)	2% (9)
It is an economic analysis (e.g. cost-benefit analysis).	1% (2)	0% (0)	0% (0)	0% (0)	0% (0)	1% (2)
None of the above / Other	44% (101)	53% (41)	42% (13)	38% (6)	71% (46)	48% (185)
Total	230	77	31	16	65	388

Table S4. Question 13 - Model motivation

	Malaria	Dengue	WNV	Filariasis	Other	Total
It is clearly a priori: the model or formula itself is based on assumptions about the process or mechanisms (e.g. Ross' or Macdonald's transmission models).	95% (219)	99% (76)	100% (31)	81% (13)	100% (65)	96% (373)
It is not clearly either one, but the intent of the analysis itself was to understand, quantify, or describe a mechanism	5% (11)	1% (1)	0% (0)	19% (3)	0% (0)	4% (15)
Total	230	77	31	16	65	388

Table S5. Question 15 - Which parameters have been or could be estimated by data presented in this paper, either by themselves (Alone) or together with other parameters (Total)?

	Malaria		Dengue		WNV		Filariasis		Other		Total	
	Alone	Total	Alone	Total	Alone	Total	Alone	Total	Alone	Total	Alone	Total
Mosquito population density	2	47	2	7	0	4	0	3	1	12	5	63
Mosquito survival	1	47	0	7	0	7	0	5	0	10	1	67
Blood feeding rates	1	46	0	8	0	4	0	5	0	10	1	63
Host search or selection by mosquitoes	1	8	0	1	0	2	0	0	0	5	1	14
Nectar feeding	0	0	0	0	0	0	0	0	0	0	0	0
Other aspects of the feeding cycle	0	7	0	1	0	0	0	0	0	1	0	9
Heterogeneous biting	5	10	0	0	0	2	0	0	0	4	5	14
Duration of the pathogen latent period in mosquitoes	1	33	1	5	0	4	0	3	0	6	2	46
Vector competence or transmission efficiency	2	35	2	10	0	6	1	3	2	9	4	56
Risk factors for clinical disease or severe outcomes	0	3	1	2	0	0	0	1	1	1	1	6
Duration of the pathogen latent period in vertebrate hosts	0	18	0	5	0	2	0	4	0	4	0	30
Duration of the infectious period in vertebrate hosts	4	30	2	10	0	4	0	9	0	8	6	53
Mosquito movement	0	2	0	1	0	1	0	0	0	0	0	4
Vertebrate host movement	0	1	0	1	0	0	0	0	0	2	0	4
None of the above / Other	24	31	16	16	6	6	1	3	2	2	48	57

Table S6. Question 15 - Which parameters have been (or could be) estimated by the data presented in this paper?

Combination size	Malaria	Dengue	WNV	Filariasis	Other	Total
1	38% (41)	69% (24)	46% (6)	15% (2)	27% (6)	44% (74)
2	17% (18)	9% (3)	0% (0)	38% (5)	23% (5)	15% (26)
3	8% (9)	3% (1)	23% (3)	15% (2)	14% (3)	8% (14)
4	9% (10)	6% (2)	0% (0)	15% (2)	9% (2)	9% (15)
5	12% (13)	3% (1)	8% (1)	15% (2)	9% (2)	9% (15)
6	7% (7)	6% (2)	8% (1)	0% (0)	5% (1)	6% (11)
7	6% (6)	0% (0)	8% (1)	0% (0)	5% (1)	4% (7)
8	2% (2)	6% (2)	0% (0)	0% (0)	5% (1)	3% (5)
9	1% (1)	0% (0)	8% (1)	0% (0)	0% (0)	1% (2)
10	0% (0)	0% (0)	0% (0)	0% (0)	5% (1)	1% (1)
Total	107	35	13	13	22	170

Table S7. Question 16 - Which transmission metrics have been or could be estimated by data presented in this paper, either by themselves (Alone) or together with other metrics (Total)?

	Malaria		Dengue		WNV		Filariasis		Other		Total	
	Alone	Total	Alone	Total	Alone	Total	Alone	Total	Alone	Total	Alone	Total
The prevalence of infection in the vertebrate host	6	54	0	6	0	5	1	8	0	15	7	75
Seroconversion rates	1	9	0	2	0	0	0	1	0	2	1	12
Clinical incidence of infection	0	16	0	8	0	1	0	4	0	10	0	30
Mortality data	0	10	0	4	0	2	0	0	0	4	0	17
The force of infection	4	37	1	17	0	4	0	5	0	12	5	63
Biting rates on humans (i.e. HBR) or other vertebrate hosts	0	36	3	5	0	4	0	2	0	8	3	49
The entomological inoculation rate	1	44	0	1	0	4	0	2	0	6	1	54
The proportion of infected or infectious mosquitoes	0	30	0	1	0	5	1	3	0	6	1	42
The infectious reservoir of pathogens in vertebrate hosts	0	17	0	2	0	4	0	4	0	9	0	30
Vectorial capacity (or a similar index)	4	44	0	7	0	4	1	6	0	14	5	60
Ro or some other reproductive number)	6	41	7	25	5	9	0	8	3	19	20	85
The pathogen importation rate	0	2	0	1	1	1	0	0	0	0	1	4
Spatial clustering / spatial scale of transmission	0	4	0	0	0	0	0	0	0	1	0	5
Pathogen resistance to drugs	0	4	0	0	0	0	0	0	0	0	0	4
Mosquito resistance to insecticides	0	1	0	0	0	0	0	0	0	0	0	1
Inherited blood disorders related to mosquito-borne pathogen transmission	0	0	0	0	0	0	0	0	0	0	0	0
Biological cost of drug resistance	0	1	0	0	0	0	0	0	0	0	0	1
Biological cost of insecticide	0	0	0	0	0	0	0	0	0	0	0	0
Coverage levels of some intervention	0	8	0	2	0	1	0	2	0	1	0	13
None	10	12	6	6	2	2	0	0	0	0	18	20
Other	4	6	0	0	0	0	0	1	0	1	4	8

Table S8. Question 16 - Which transmission metrics have been (or could be) estimated by data presented in this paper?

Combination size	Malaria	Dengue	WNV	Filariasis	Other	Total
1	34% (36)	49% (17)	62% (8)	23% (3)	14% (3)	39% (66)
2	20% (21)	20% (7)	8% (1)	23% (3)	14% (3)	18% (31)
3	14% (15)	11% (4)	0% (0)	8% (1)	5% (1)	11% (19)
4	7% (7)	3% (1)	0% (0)	23% (3)	23% (5)	6% (11)
5	4% (4)	6% (2)	0% (0)	0% (0)	5% (1)	4% (6)
6	3% (3)	6% (2)	0% (0)	8% (1)	9% (2)	3% (5)
7	3% (3)	3% (1)	0% (0)	8% (1)	9% (2)	3% (5)
8	4% (4)	0% (0)	8% (1)	0% (0)	5% (1)	4% (6)
9	8% (9)	0% (0)	15% (2)	8% (1)	14% (3)	8% (13)
10	3% (3)	0% (0)	8% (1)	0% (0)	5% (1)	3% (5)
11	1% (1)	3% (1)	0% (0)	0% (0)	0% (0)	1% (2)
12	1% (1)	0% (0)	0% (0)	0% (0)	0% (0)	1% (1)
Total	107	35	13	13	22	170

Table S9. Question 17 - Data description

	Malaria		Dengue		WNV		Filariasis		Other		Total	
	Alone	Total	Alone	Total	Alone	Total	Alone	Total	Alone	Total	Alone	Total
The data are being published for the first time in peer review	18	37	8	8	0	1	0	0	4	4	25	45
The data have already been published	22	48	14	15	4	12	4	5	4	5	44	79
This is a compilation of data aggregated from multiple sources	19	49	5	6	1	8	5	6	5	6	32	70
None of the above / Other	13	18	7	7	0	1	3	3	8	8	25	31

Table S10. Question 17 - Data description

Combination size	Malaria	Dengue	WNV	Filariasis	Other	Total
1	67% (72)	97% (34)	38% (5)	92% (12)	95% (21)	74% (126)
2	23% (25)	3% (1)	54% (7)	8% (1)	5% (1)	19% (33)
3	9% (10)	0% (0)	8% (1)	0% (0)	0% (0)	6% (11)
Total	107	35	13	13	22	170

Table S11. Question 20 - Work based on / critique existing method?

	Malaria	Dengue	WNV	Filariasis	Other	Total
DENSIM/SIMSIM	0% (0)	4% (3)	0% (0)	0% (0)	0% (0)	1% (3)
EPIFIL	0% (0)	0% (0)	0% (0)	25% (4)	0% (0)	1% (4)
HYDREMATS	7% (16)	3% (2)	6% (2)	19% (3)	9% (6)	6% (24)
LYMFASIM	0% (0)	0% (0)	0% (0)	6% (1)	0% (0)	0% (1)
None of the above / Other	34% (78)	52% (40)	58% (18)	31% (5)	42% (27)	41% (160)
The Garki model	10% (24)	1% (1)	3% (1)	0% (0)	0% (0)	7% (26)
The Imperial College Malaria Model	1% (3)	0% (0)	0% (0)	0% (0)	0% (0)	1% (3)
The McKenzie model	3% (7)	0% (0)	0% (0)	0% (0)	0% (0)	2% (7)
The Ross-Macdonald theory of transmission	43% (100)	40% (31)	32% (10)	19% (3)	49% (32)	41% (158)
The Strain Theory of Malaria Transmission	0% (0)	0% (0)	0% (0)	0% (0)	0% (0)	0% (0)
The Swiss Tropical Institute Malaria Model	1% (2)	0% (0)	0% (0)	0% (0)	0% (0)	1% (2)
Total	230	77	31	16	65	388

Table S12. Question 21 - Is this paper concerned with population genetics or evolution?

	Malaria	Dengue	WNV	Filariasis	Other	Total
Yes	5% (12)	6% (5)	0% (0)	0% (0)	5% (3)	5% (18)
No	95% (218)	94% (72)	100% (31)	100% (16)	95% (62)	95% (370)
Total	230	77	31	16	65	388

Table S13. Question 22 - If evolution was considered, what aspects were included?

	Malaria		Dengue		WNV		Filariasis		Other		Total	
	Alone	Total	Alone	Total	Alone	Total	Alone	Total	Alone	Total	Alone	Total
Evolving mosquito populations	2	3	0	1	0	0	0	0	0	1	2	4
Evolving pathogen populations	3	9	0	3	0	0	0	0	1	3	4	13
Evolving vertebrate host populations	0	0	0	0	0	0	0	0	0	0	0	0
Pathogen transmission dynamics	1	6	1	4	0	0	0	0	0	1	2	10
Population dynamics	0	1	0	2	0	0	0	0	0	0	0	3
Gene or genotype frequencies	0	2	0	1	0	0	0	0	0	1	0	3
None of the above / Other	0	0	0	0	0	0	0	0	0	0	0	0

TABLE S14. Question 22 - If evolution was considered, what aspects were included?

Combination size	Malaria	Dengue	WNV	Filariasis	Other	Total
1	50% (6)	20% (1)	– (0)	– (0)	33% (1)	44% (8)
2	25% (3)	40% (2)	– (0)	– (0)	33% (1)	28% (5)
3	25% (3)	40% (2)	– (0)	– (0)	33% (1)	28% (5)
Total	12	5	0	0	3	18

TABLE S15. Question 24 - Which pathogens are discussed?

	Total	
	Alone	Total
A generic pathogen	19	28
Malaria parasites	212	230
Filarial parasites	10	16
Dengue fever	70	77
West Nile fever	30	31
Rift Valley fever	3	3
Yellow Fever Virus	0	3
A generic arbovirus	4	6
None of the above / Other	11	29

TABLE S16. Question 24 - Which pathogens are discussed?

Combination size	Malaria	Dengue	WNV	Filariasis	Other	Total
1	92% (212)	91% (70)	97% (30)	62% (10)	55% (36)	93% (359)
2	5% (12)	5% (4)	3% (1)	19% (3)	35% (23)	6% (23)
3	3% (6)	4% (3)	0% (0)	19% (3)	9% (6)	2% (6)
Total	230	77	31	16	65	388

2. Basic

Table S17. Question 25 - How were aquatic populations modeled?

	Malaria	Dengue	WNV	Filariasis	Other	Total
Explicitly: Aquatic populations were modeled with at least one state variable	5% (12)	22% (17)	32% (10)	0% (0)	9% (6)	12% (45)
Implicitly: The emergence rate of adult mosquitoes was described by a parameter or function but no state variable	20% (44)	21% (16)	29% (9)	0% (0)	14% (9)	20% (75)
Not at all	74% (162)	57% (43)	39% (12)	100% (13)	77% (50)	68% (252)
Other	0% (1)	0% (0)	0% (0)	0% (0)	0% (0)	0% (1)
Total	219	76	31	13	65	373

Table S18. Question 26 - Which one of the following best describes the way adult mosquito population and infection dynamics were modeled?

	Malaria	Dengue	WNV	Filariasis	Other	Total
Explicitly: Adult mosquito populations were modeled with at least one state variable	55% (121)	70% (53)	90% (28)	15% (2)	68% (44)	62% (231)
Implicitly : There was a parameter describing host exposure.	14% (31)	8% (6)	0% (0)	15% (2)	3% (2)	11% (40)
Implicitly : Transmission modeled dynamically with terms associated with Ross-Macdonald model and an infectious reservoir.	17% (37)	4% (3)	3% (1)	31% (4)	3% (2)	12% (45)
Implicitly : Transmission modeled dynamically similarly to directly transmitted disease.	5% (12)	16% (12)	3% (1)	23% (3)	15% (10)	9% (32)
Not at all	0% (1)	0% (0)	0% (0)	0% (0)	2% (1)	1% (2)
Other	8% (17)	3% (2)	3% (1)	15% (2)	9% (6)	6% (23)
Total	219	76	31	13	65	373

Table S19. Question 27 - How were infections in vertebrate hosts modeled modeled?

	Malaria	Dengue	WNV	Filariasis	Other	Total
Explicitly: pathogen infection dynamics in vertebrate hosts were modeled with at least one state variable	80% (175)	92% (70)	94% (29)	69% (9)	91% (59)	85% (318)
Implicitly: mosquito exposure to the infectious reservoir in vertebrate hosts was described by a parameter	11% (23)	4% (3)	3% (1)	8% (1)	2% (1)	7% (27)
Not at all	2% (4)	0% (0)	0% (0)	0% (0)	2% (1)	1% (5)
Other	8% (17)	4% (3)	3% (1)	23% (3)	6% (4)	6% (23)
Total	219	76	31	13	65	373

Table S20. Question 28 - How many spatial locations were considered?

	Malaria	Dengue	WNV	Filariasis	Other	Total
One place with no emigration or emigration; or location was undefined or vaguely defined (i.e. the model was spaceless)	4% (8)	9% (7)	3% (1)	0% (0)	6% (4)	5% (17)
There was more than one location or place; or the model included terms describing immigration or emigration of mosquitoes or vertebrate hosts	17% (37)	13% (10)	19% (6)	0% (0)	20% (13)	17% (64)
Other	79% (173)	77% (58)	77% (24)	100% (13)	74% (48)	78% (290)
Total	218	75	31	13	65	371

Table S21. Question 29 - How many mosquito taxa, genotypes or phenotypes were considered?

	Malaria	Dengue	WNV	Filariasis	Other	Total
0	8% (17)	7% (5)	0% (0)	33% (4)	10% (6)	8% (29)
1	89% (185)	88% (67)	100% (31)	42% (5)	83% (52)	88% (319)
2	3% (7)	5% (4)	0% (0)	25% (3)	8% (5)	4% (15)
More than 2	0% (0)	0% (0)	0% (0)	0% (0)	0% (0)	0% (0)
Total	209	76	31	12	63	363

Table S22. Question 30 - How many pathogen taxa, genotypes or phenotypes were considered?

	Malaria	Dengue	WNV	Filariasis	Other	Total
1	90% (180)	77% (50)	100% (31)	67% (8)	89% (54)	88% (303)
2	10% (19)	23% (15)	0% (0)	33% (4)	11% (7)	12% (40)
More than 2	0% (0)	0% (0)	0% (0)	0% (0)	0% (0)	0% (0)
Total	199	65	31	12	61	343

Table S23. Question 31 - How many vertebrate taxa, genotypes or phenotypes were considered?

	Malaria	Dengue	WNV	Filariasis	Other	Total
0	1% (3)	0% (0)	0% (0)	0% (0)	0% (0)	1% (3)
1	92% (193)	99% (74)	73% (19)	100% (13)	89% (55)	92% (330)
2	6% (13)	1% (1)	27% (7)	0% (0)	11% (7)	7% (24)
More than 2	0% (0)	0% (0)	0% (0)	0% (0)	0% (0)	0% (0)
Total	209	75	26	13	62	357

Table S24. Question 32 - Did the model consider any type of control?

	Malaria	Dengue	WNV	Filariasis	Other	Total
No	53% (117)	75% (57)	68% (21)	54% (7)	80% (52)	63% (234)
Yes	47% (102)	25% (19)	32% (10)	46% (6)	20% (13)	37% (139)
Total	219	76	31	13	65	373

3. Spatial Dynamics

Table S25. Question 33 - How many spatial locations were included in or implied by the model?

	Malaria	Dengue	WNV	Filariasis	Other	Total
One patch (with migration)	27% (10)	0% (0)	17% (1)	– (0)	8% (1)	19% (12)
Space was continuous (e.g. in PDEs or in an IBM)	16% (6)	20% (2)	50% (3)	– (0)	23% (3)	22% (14)
The model described a connectivity network among individual hosts	0% (0)	10% (1)	0% (0)	– (0)	0% (0)	2% (1)
Three or more patches or locations	46% (17)	50% (5)	33% (2)	– (0)	54% (7)	48% (31)
Two patches or locations	11% (4)	20% (2)	0% (0)	– (0)	8% (1)	8% (5)
Other	0% (0)	0% (0)	0% (0)	– (0)	8% (1)	2% (1)
Total	37	10	6	0	13	64

Table S26. Question 34 - How were the multiple pathes spatially oriented?

	Malaria	Dengue	WNV	Filariasis	Other	Total
A network of interconnected patches or points	33% (5)	60% (3)	0% (0)	– (0)	43% (3)	38% (11)
In a grid or lattice	60% (9)	40% (2)	0% (0)	– (0)	43% (3)	48% (14)
In an array	7% (1)	0% (0)	100% (2)	– (0)	14% (1)	14% (4)
Total	15	5	2	0	7	29

Table S27. Question 35 - What moves?

	Malaria	Dengue	WNV	Filariasis	Other	Total
Both vertebrate hosts and mosquito hosts move	22% (8)	50% (5)	67% (4)	– (0)	54% (7)	38% (24)
Mosquito hosts move	27% (10)	20% (2)	0% (0)	– (0)	15% (2)	22% (14)
Nothing	16% (6)	0% (0)	0% (0)	– (0)	0% (0)	9% (6)
Vertebrate hosts move	35% (13)	30% (3)	33% (2)	– (0)	31% (4)	31% (20)
Total	37	10	6	0	13	64

4. Aquatic Mosquito Ecology

Table S28. Question 36 - Which of the following best describes the parameter or function describing adult mosquito emergence?

	Malaria	Dengue	WNV	Filariasis	Other	Total
A constant	84% (37)	69% (11)	78% (7)	– (0)	56% (5)	79% (59)
A sinusoidal function, arbitrarily defined	2% (1)	6% (1)	0% (0)	– (0)	11% (1)	4% (3)
Other	14% (6)	25% (4)	22% (2)	– (0)	33% (3)	17% (13)
Total	44	16	9	0	9	75

Table S29. Question 37 - What factors were included in the model of larval populations?

	Malaria		Dengue		WNV		Filariasis		Other		Total	
	Alone	Total	Alone	Total	Alone	Total	Alone	Total	Alone	Total	Alone	Total
Larval dynamics were density independent	2	2	3	3	7	7	0	0	1	1	13	13
Logistic-style carrying capacity	5	6	6	8	0	0	0	0	2	3	13	17
Power-law	0	0	0	0	0	0	0	0	0	0	0	0
Resource-based competition	1	2	1	3	1	2	0	0	0	0	3	7
Predation	1	2	0	1	0	0	0	0	0	0	1	3
None of the above / Other	1	2	4	5	1	2	0	0	2	3	8	12

Table S30. Question 37 - What factors were included in the model of larval populations?

Combination size	Malaria	Dengue	WNV	Filariasis	Other	Total
1	83% (10)	82% (14)	90% (9)	– (0)	83% (5)	84% (38)
2	17% (2)	18% (3)	10% (1)	– (0)	17% (1)	16% (7)
Total	12	17	10	0	6	45

Table S31. Question 38 - What other features of aquatic populations were modeled, either by themselves (Alone) or together with other features (Total)?

	Malaria		Dengue		WNV		Filariasis		Other		Total	
	Alone	Total	Alone	Total	Alone	Total	Alone	Total	Alone	Total	Alone	Total
Aquatic habitat was created and destroyed by rainfall / dessication	0	7	0	2	0	0	0	0	0	2	0	11
Aquatic populations were lost through flushing / overfilling of habitat	0	4	0	2	0	0	0	0	0	0	0	6
Development of immature stages was temperature dependent	1	11	3	9	0	0	0	0	2	4	6	24
There was larval competition with other mosquito species	0	0	0	0	0	0	0	0	0	0	0	0
Egg populations were explicitly modeled	0	6	2	8	0	0	0	0	0	4	2	18
Larval age	0	1	0	4	1	1	0	0	0	0	1	6
Larval stages (i.e. instars)	0	3	0	3	0	0	0	0	0	0	0	6
Dormancy / long-term persistence of the eggs was possible	0	1	0	0	0	0	0	0	0	1	0	2
None of the above / Other	41	45	19	23	18	18	0	0	9	10	84	93

Table S32. Question 38 - What other features of aquatic populations were modeled?

Combination size	Malaria	Dengue	WNV	Filariasis	Other	Total
1	75% (42)	73% (24)	100% (19)	– (0)	73% (11)	78% (93)
2	16% (9)	12% (4)	0% (0)	– (0)	13% (2)	12% (15)
3	4% (2)	9% (3)	0% (0)	– (0)	13% (2)	6% (7)
4	5% (3)	0% (0)	0% (0)	– (0)	0% (0)	2% (3)
5	0% (0)	6% (2)	0% (0)	– (0)	0% (0)	2% (2)
Total	56	33	19	0	15	120

Table S33. Question 39 - How did aquatic ecology differ across space?

	Malaria	Dengue	WNV	Filariasis	Other	Total
Different rates or functions were used to illustrate a general principle	12% (2)	20% (1)	25% (1)	– (0)	50% (1)	19% (5)
Different rates or functions were used, based on data	38% (6)	20% (1)	0% (0)	– (0)	0% (0)	26% (7)
The same rates or functions applied everywhere	50% (8)	60% (3)	75% (3)	– (0)	50% (1)	56% (15)
Total	16	5	4	0	2	27

5. Quasi-Direct Transmission

Table S34. Question 40 - Which one of the following best describes the contact parameter (in models with quasi-direct transmission)?

	Malaria	Dengue	WNV	Filariasis	Other	Total
Constant	89% (8)	75% (9)	100% (1)	100% (1)	100% (5)	83% (20)
Constant and fitted to local data	11% (1)	0% (0)	0% (0)	0% (0)	0% (0)	4% (1)
Seasonally forced, with a sinusoidal function	0% (0)	25% (3)	0% (0)	0% (0)	0% (0)	12% (3)
Total	9	12	1	1	5	24

6. Minimized Mosquito Infection Dynamics

Table S35. Question 42 -Was the minimal mosquito assumption implemented with a delay for the explicit incubation period?

	Malaria	Dengue	WNV	Filariasis	Other	Total
No	66% (33)	83% (5)	0% (0)	100% (4)	100% (2)	70% (44)
Yes	34% (17)	17% (1)	100% (1)	0% (0)	0% (0)	30% (19)
Total	50	6	1	4	2	63

Table S36. Question 43 - Which one of the following best describes the way in which the minimal mosquito assumption was implemented?

	Malaria	Dengue	WNV	Filariasis	Other	Total
Vectorial capacity (or EIR or Ro) was constant	49% (23)	80% (4)	100% (1)	100% (1)	0% (0)	53% (29)
Vectorial capacity (or EIR or Ro) was constant and fitted to local data	26% (12)	20% (1)	0% (0)	0% (0)	100% (1)	25% (14)
Vectorial capacity (or EIR or Ro) was realistically forced using mosquito population data	26% (12)	0% (0)	0% (0)	0% (0)	0% (0)	22% (12)
Total	47	5	1	1	1	55

7. Adult Mosquito Demography and Feeding Behavior

Table S37. Question 45 - How was adult mosquito density modeled?

	Malaria	Dengue	WNV	Filariasis	Other	Total
A sinusoidal function	10% (7)	0% (0)	11% (1)	0% (0)	7% (2)	8% (10)
Based on a pattern derived from data	4% (3)	10% (2)	11% (1)	50% (1)	7% (2)	6% (7)
Constant	56% (40)	81% (17)	44% (4)	50% (1)	70% (21)	61% (72)
None of the above / Other	30% (21)	10% (2)	33% (3)	0% (0)	17% (5)	25% (30)
Total	71	21	9	2	30	119

Table S38. Question 46 - What assumptions were made about adult mosquito mortality in the absence of control?

	Malaria		Dengue		WNV		Filariasis		Other		Total	
	Alone	Total	Alone	Total	Alone	Total	Alone	Total	Alone	Total	Alone	Total
Constant per-capita mortality (i.e. exponentially or geometrically distributed lifespans)	93	99	46	46	23	24	1	1	35	36	182	190
With temperature dependent mortality	10	11	3	3	0	0	0	0	2	3	15	17
With humidity dependent mortality	0	0	0	0	0	0	0	0	0	0	0	0
With senescence (i.e. age dependent mortality)	1	1	1	1	0	0	0	0	1	1	2	2
With frailty (i.e. different lifespans for different mosquito types)	0	0	0	0	0	0	0	0	0	0	0	0
Mosquito mortality was not part of this model	3	3	2	2	1	1	1	1	3	3	10	10
None of the above / Other	8	13	1	1	3	4	0	0	2	2	14	20

Table S39. Question 46 - What assumptions were made about adult mosquito mortality in the absence of control?

Combination size	Malaria	Dengue	WNV	Filariasis	Other	Total
1	95% (115)	100% (53)	96% (27)	100% (2)	98% (43)	97% (223)
2	5% (6)	0% (0)	4% (1)	0% (0)	2% (1)	3% (8)
Total	121	53	28	2	44	231

TABLE S40. Question 47 - What assumptions were made about mosquito blood feeding rates in the absence of control?

	Malaria		Dengue		WNV		Filariasis		Other		Total	
	Alone	Total	Alone	Total	Alone	Total	Alone	Total	Alone	Total	Alone	Total
Blood feeding occurred at a constant per-capita rate	88	90	38	38	21	23	1	2	32	35	165	172
Blood feeding rates depended explicitly on the outcome of searching	5	7	1	1	0	0	0	0	0	1	6	9
Blood feeding rates varied with temperature	9	9	3	6	0	0	0	0	2	3	14	18
Blood feeding rates varied with the availability of vertebrate hosts or their behavior	1	6	2	3	0	3	0	0	2	5	5	16
Blood feeding rates varied were altered by control	1	7	0	0	0	1	0	0	0	1	1	8
Blood feeding was not considered in this model	2	2	1	1	3	3	0	0	0	1	6	7
None of the above / Other	8	10	5	7	1	3	0	1	2	4	16	24

Table S41. Question 47 - What assumptions were made about mosquito blood feeding rates in the absence of control?

Combination size	Malaria	Dengue	WNV	Filariasis	Other	Total
1	93% (114)	94% (50)	89% (25)	50% (1)	86% (38)	92% (213)
2	6% (7)	6% (3)	4% (1)	50% (1)	14% (6)	7% (16)
3	1% (1)	0% (0)	7% (2)	0% (0)	0% (0)	1% (3)
Total	122	53	28	2	44	232

Table S42. Question 48 - What assumption was made about the proportion of bloodmeals taken on the pathogen's host(s)?

	Malaria	Dengue	WNV	Filariasis	Other	Total
A constant fraction of mosquitoes fed on each vertebrate host species	14% (17)	6% (3)	14% (4)	0% (0)	18% (8)	12% (27)
Feeding on other vertebrate hosts was included only impliticly or not at all	79% (95)	89% (47)	68% (19)	100% (2)	75% (33)	82% (189)
Feeding on vertebrate hosts was modeled with a frequency dependent function	5% (6)	6% (3)	11% (3)	0% (0)	5% (2)	4% (10)
Host selection was modeled as the outcome of a search algorithm (e.g. in an ibm)	2% (2)	0% (0)	0% (0)	0% (0)	0% (0)	1% (2)
Other	1% (1)	0% (0)	7% (2)	0% (0)	2% (1)	1% (3)
Total	121	53	28	2	44	231

Table S43. Question 49 - What aspects of mosquito behavior were spatially heterogeneous?

	Malaria		Dengue		WNV		Filariasis		Other		Total	
	Alone	Total	Alone	Total	Alone	Total	Alone	Total	Alone	Total	Alone	Total
Adult mosquito survival	0	3	0	0	0	1	0	0	0	0	0	4
Pathogen development rates, based on temperature	0	3	0	0	0	0	0	0	0	0	0	3
Feeding rates	0	6	0	1	0	1	0	0	1	2	1	10
The availability of vertebrate hosts	4	9	1	3	0	0	0	0	4	5	7	15
None	16	16	6	6	5	5	0	0	4	4	31	31
Other	1	3	0	1	0	0	0	0	0	1	1	5

Table S44. Question 49 - What aspects of mosquito behavior were spatially heterogeneous?

Combination size	Malaria	Dengue	WNV	Filariasis	Other	Total
1	75% (21)	78% (7)	83% (5)	– (0)	90% (9)	78% (40)
2	11% (3)	22% (2)	17% (1)	– (0)	0% (0)	12% (6)
3	11% (3)	0% (0)	0% (0)	– (0)	10% (1)	8% (4)
4	4% (1)	0% (0)	0% (0)	– (0)	0% (0)	2% (1)
Total	28	9	6	0	10	51

Table S45. Question 50 - Which of these other aspects of adult mosquitoes were included in the model?

	Malaria		Dengue		WNV		Filariasis		Other		Total	
	Alone	Total	Alone	Total	Alone	Total	Alone	Total	Alone	Total	Alone	Total
Egg Laying	10	14	11	11	1	1	0	0	2	4	24	30
Mating	1	2	1	1	0	0	0	0	0	0	2	3
Resting	2	5	0	0	0	0	0	0	0	1	2	6
Nectar Feeding	0	0	0	0	0	0	0	0	0	0	0	0
Male Mosquito Populations	1	1	0	0	0	0	0	0	0	0	1	1
None of the above / Other	103	103	41	41	27	27	2	2	40	41	196	197

Table S46. Question 50 - Which of these other aspects of adult mosquitoes were included in the model?

Combination size	Malaria	Dengue	WNV	Filariasis	Other	Total
1	97% (117)	100% (53)	100% (28)	100% (2)	95% (42)	97% (225)
2	3% (4)	0% (0)	0% (0)	0% (0)	5% (2)	3% (6)
Total	121	53	28	2	44	231

8. Pathogen Infection Dynamics in Mosquitoes

Table S47. Question 51 - Which one of the following best describes the way infections in mosquitoes were modeled?

	Malaria	Dengue	WNV	Filariasis	Other	Total
A delay differential equation, and based on some kind of compartment model	9% (11)	13% (7)	0% (0)	0% (0)	7% (3)	9% (20)
A function or formula, but no state variable or equation	7% (9)	4% (2)	0% (0)	50% (1)	7% (3)	5% (11)
Not at all	2% (3)	0% (0)	0% (0)	0% (0)	0% (0)	1% (3)
ODE: Some kind of a compartment model	48% (58)	64% (34)	75% (21)	0% (0)	50% (22)	55% (126)
PDE: Independent variables described host age or space, but the infection dynamics were based on a compartment model	6% (7)	4% (2)	11% (3)	0% (0)	5% (2)	6% (13)
PDE: One or more independent variable described the infection	1% (1)	0% (0)	0% (0)	0% (0)	0% (0)	0% (1)
With a stochastic simulation that was based on some kind of compartment model	10% (12)	9% (5)	0% (0)	50% (1)	9% (4)	10% (22)
With an individual-based computer simulation (other than the ones described above)	4% (5)	2% (1)	0% (0)	0% (0)	11% (5)	5% (11)
With difference equations (i.e. a fixed time step) that were based on some kind of compartment model	8% (10)	4% (2)	11% (3)	0% (0)	11% (5)	8% (18)
With some other kind of difference equation	1% (1)	0% (0)	0% (0)	0% (0)	0% (0)	0% (1)
Other	3% (4)	0% (0)	4% (1)	0% (0)	0% (0)	2% (5)
Total	121	53	28	2	44	231

Table S48. Question 53 - Did the model consider pathogen latency in mosquitoes?

	Malaria	Dengue	WNV	Filariasis	Other	Total
Explicitly: the latent period was part of the model structure	56% (68)	56% (29)	50% (14)	100% (2)	43% (19)	54% (125)
Implicitly: mortality during the latent period was considered, but not with a state variable	11% (13)	10% (5)	0% (0)	0% (0)	14% (6)	7% (17)
Not at all	33% (40)	35% (18)	50% (14)	0% (0)	43% (19)	38% (88)
Total	121	52	28	2	44	230

TABLE S49. Question 54 - Was the pathogen development rate in the mosquito temperature dependent?

	Malaria	Dengue	WNV	Filariasis	Other	Total
No	81% (66)	85% (29)	100% (14)	100% (2)	88% (22)	84% (119)
Yes	19% (15)	15% (5)	0% (0)	0% (0)	12% (3)	16% (23)
Total	81	34	14	2	25	142

Table S50. Question 55 - Which of these other aspects of transmission by mosquitoes were included in the model?

	Malaria		Dengue		WNV		Filariasis		Other		Total	
	Alone	Total	Alone	Total	Alone	Total	Alone	Total	Alone	Total	Alone	Total
Vertical Transmission	0	0	7	7	4	4	0	0	2	2	13	13
Recovery from Infection / Waning Infection	0	0	0	0	2	2	0	0	0	0	2	2
A Cost of Infection (i.e. virulence)	6	6	0	0	0	0	0	0	2	2	7	7
Mosquito Superinfection	5	6	0	0	0	0	0	0	0	0	5	6
None of the above / Other	109	110	46	46	22	22	2	2	40	40	203	204

Table S51. Question 55 - Which of these other aspects of transmission by mosquitoes were included in the model?

Combination size	Malaria	Dengue	WNV	Filariasis	Other	Total
1	99% (120)	100% (53)	100% (28)	100% (2)	100% (44)	100% (230)
2	1% (1)	0% (0)	0% (0)	0% (0)	0% (0)	0% (1)
Total	121	53	28	2	44	231

Table S52. Question 56 - How did the vector species or types differ?

	Malaria		Dengue		WNV		Filariasis		Other		Total	
	Alone	Total	Alone	Total	Alone	Total	Alone	Total	Alone	Total	Alone	Total
Population density	0	5	0	2	0	0	0	0	0	3	0	6
Lifespan	0	7	0	2	0	0	0	0	0	3	0	8
Feeding rates	1	10	0	3	0	0	0	0	1	4	1	12
Host feeding preferences	1	8	0	0	0	0	0	1	0	1	1	8
Resting behavior	0	1	0	0	0	0	0	0	0	0	0	1
Vertical transmission	0	0	0	0	0	0	0	0	1	2	1	2
Movement patterns	0	0	0	1	0	0	0	0	0	0	0	1
Seasonal patterns	0	1	0	0	0	0	0	0	0	0	0	1
Infectivity: their susceptibility to infection	1	8	0	2	0	0	0	0	0	2	1	8
Infectivity: the efficiency of transmission from an infectious mosquito	0	6	0	2	0	0	3	4	0	3	3	9
Insecticide resistance	1	1	0	0	0	0	0	0	0	0	1	1
None of the above / Other	0	1	1	1	0	0	0	0	1	2	2	4

Table S53. Question 56 - How did the vector species or types differ?

Combination size	Malaria	Dengue	WNV	Filariasis	Other	Total
1	25% (4)	25% (1)	– (0)	75% (3)	43% (3)	42% (10)
2	25% (4)	25% (1)	– (0)	25% (1)	14% (1)	21% (5)
3	12% (2)	0% (0)	– (0)	0% (0)	0% (0)	8% (2)
4	12% (2)	0% (0)	– (0)	0% (0)	0% (0)	8% (2)
5	12% (2)	50% (2)	– (0)	0% (0)	43% (3)	12% (3)
6	12% (2)	0% (0)	– (0)	0% (0)	0% (0)	8% (2)
Total	16	4	0	4	7	24

Table S54. Question 57 - Did the paper call the different mosquito variants species or were they different types (e.g. genotypes / phenotypes) of the same species?

	Malaria	Dengue	WNV	Filariasis	Other	Total
Different species	88% (14)	50% (2)	– (0)	100% (4)	100% (7)	83% (20)
Different types of the same species	12% (2)	25% (1)	– (0)	0% (0)	0% (0)	12% (3)
There was no distinction made	0% (0)	25% (1)	– (0)	0% (0)	0% (0)	4% (1)
Other	0% (0)	0% (0)	– (0)	0% (0)	0% (0)	0% (0)
Total	16	4	0	4	7	24

9. Vertebrate Host Population Dynamics

Table S55. Question 58 - Which of the following did the model consider?

	Malaria		Dengue		WNV		Filariasis		Other		Total	
	Alone	Total	Alone	Total	Alone	Total	Alone	Total	Alone	Total	Alone	Total
Vertebrate host nutritional status	0	5	0	0	0	0	0	0	0	0	0	5
Vertebrate host genotype	0	0	0	0	1	1	0	0	0	0	1	1
Vertebrate host defensive behavior (i.e. swatting)	1	1	0	0	0	1	0	0	0	0	1	2
Co-infection with other pathogens (e.g. HIV, TB)	0	2	0	2	0	0	0	1	0	2	0	4
Vertebrate host age	29	36	6	6	2	2	4	7	7	10	47	57
None of the above / Other	136	140	62	64	25	26	2	5	48	52	255	264

Table S56. Question 58 - Which of the following did the model consider?

Combination size	Malaria	Dengue	WNV	Filariasis	Other	Total
1	95% (166)	97% (68)	97% (28)	67% (6)	93% (55)	96% (304)
2	5% (9)	3% (2)	3% (1)	22% (2)	5% (3)	4% (13)
3	0% (0)	0% (0)	0% (0)	11% (1)	2% (1)	0% (1)
Total	175	70	29	9	59	318

Table S57. Question 59 - Did the model include any vital dynamics for the vertebrate host populations?

	Malaria	Dengue	WNV	Filariasis	Other	Total
No	51% (90)	14% (10)	3% (1)	33% (3)	41% (24)	37% (119)
Yes	49% (85)	86% (60)	97% (28)	67% (6)	59% (35)	63% (199)
Total	175	70	29	9	59	318

Table S58. Question 60 - How were birth rates modeled?

	Malaria	Dengue	WNV	Filariasis	Other	Total
Birth rates were not modeled	11% (9)	5% (3)	39% (11)	0% (0)	17% (6)	13% (25)
Population birth rate was constant	35% (30)	53% (32)	29% (8)	17% (1)	31% (11)	39% (77)
Population birth rates were constant, per-capita	32% (27)	30% (18)	25% (7)	33% (2)	20% (7)	30% (59)
Population birth rates were described by a time dependent parameter or function	2% (2)	2% (1)	4% (1)	0% (0)	11% (4)	4% (7)
Population change was described by the Lotka-Volterra equation	2% (2)	2% (1)	0% (0)	0% (0)	0% (0)	2% (3)
There was age-structure with age-specific birth rates	2% (2)	3% (2)	0% (0)	0% (0)	3% (1)	3% (5)
Other	15% (13)	5% (3)	4% (1)	50% (3)	17% (6)	12% (23)
Total	85	60	28	6	35	199

Table S59. Question 61 - How were vertebrate host deaths modeled in the absence of disease?

	Malaria	Dengue	WNV	Filariasis	Other	Total
Constant per-capita death rates	86% (73)	92% (55)	79% (22)	100% (6)	89% (31)	87% (174)
Death rate dependent on nutritional status or coinfection	6% (5)	0% (0)	4% (1)	0% (0)	0% (0)	3% (6)
Not at all	4% (3)	2% (1)	11% (3)	0% (0)	9% (3)	4% (8)
Other	5% (4)	7% (4)	7% (2)	0% (0)	3% (1)	6% (11)
Total	85	60	28	6	35	199

Table S60. Question 62 - What aspects of the vertebrate host populations were spatially heterogeneous?

	Malaria		Dengue		WNV		Filariasis		Other		Total	
	Alone	Total	Alone	Total	Alone	Total	Alone	Total	Alone	Total	Alone	Total
Heterogeneous population distributions	9	10	2	3	0	0	0	0	7	7	18	20
Heterogeneous genetic composition	0	0	0	0	0	0	0	0	0	0	0	0
Heterogeneous behavior	0	0	0	1	0	0	0	0	0	0	0	1
Heterogeneous economic status	0	0	0	0	0	0	0	0	0	0	0	0
Heterogeneous cultural practices	0	0	0	0	0	0	0	0	0	0	0	0
Heterogeneous household types	0	0	0	0	0	0	0	0	0	0	0	0
None of the above / Other	18	19	6	6	6	6	0	0	6	6	34	35

Table S61. Question 62 - What aspects of the vertebrate host populations were spatially heterogeneous?

Combination size	Malaria	Dengue	WNV	Filariasis	Other	Total
1	96% (27)	89% (8)	100% (6)	– (0)	100% (13)	96% (52)
2	4% (1)	11% (1)	0% (0)	– (0)	0% (0)	4% (2)
Total	28	9	6	0	13	54

10. Vertebrate Host Infections

Table S62. Question 63 - Which one of the following best describes the way infections in vertebrate hosts were modeled?

	Malaria	Dengue	WNV	Filariasis	Other	Total
A delay differential equation, and based on some kind of compartment model	5% (8)	3% (2)	0% (0)	0% (0)	0% (0)	3% (10)
DE: With difference equations (i.e. discrete time step) that were based on some kind of compartment model	6% (10)	0% (0)	14% (4)	0% (0)	8% (5)	5% (17)
ODE: Some kind of a compartment model with ordinary differential equations	59% (102)	81% (57)	72% (21)	22% (2)	59% (35)	63% (200)
PDE: Independent variables described host age or space, but the infection dynamics were based on a compartment model	6% (10)	1% (1)	10% (3)	0% (0)	5% (3)	5% (17)
PDE: One or more independent variable described the infection	2% (4)	0% (0)	0% (0)	0% (0)	2% (1)	1% (4)
With a stochastic simulation that was based on some kind of compartment model	10% (17)	13% (9)	0% (0)	0% (0)	10% (6)	10% (32)
With an individual-based computer simulation (other than the ones described above)	7% (13)	1% (1)	0% (0)	0% (0)	8% (5)	6% (19)
With some other kind of difference equation	1% (2)	0% (0)	0% (0)	11% (1)	0% (0)	1% (3)
Other	5% (8)	0% (0)	3% (1)	67% (6)	7% (4)	5% (15)
Total	174	70	29	9	59	317

Table S63. Question 64 - Select the type of compartment model for the host.

	Malaria	Dengue	WNV	Filariasis	Other	Total
Other compartment model (not exactly in this list)	31% (54)	19% (13)	31% (9)	67% (6)	20% (12)	27% (85)
SEIN-SEN (As in the Garki Model: N means infected but not infectious, the second set of compartments is partially immun	5% (8)	0% (0)	0% (0)	0% (0)	0% (0)	3% (8)
SEIR	6% (10)	33% (23)	0% (0)	0% (0)	15% (9)	13% (40)
SEIRS	3% (6)	1% (1)	0% (0)	0% (0)	2% (1)	2% (7)
SEIS	5% (8)	1% (1)	0% (0)	0% (0)	0% (0)	3% (9)
SEIS-SEIS (an SEIS model with two immune stages)	0% (0)	0% (0)	0% (0)	0% (0)	0% (0)	0% (0)
SIR	6% (11)	36% (25)	52% (15)	11% (1)	36% (21)	20% (65)
SIRS	10% (17)	3% (2)	7% (2)	0% (0)	10% (6)	7% (23)
SIS	33% (57)	1% (1)	10% (3)	22% (2)	17% (10)	23% (73)
SIS-SIS (an SIS model with two immune stages)	2% (4)	6% (4)	0% (0)	0% (0)	0% (0)	3% (8)
Total	175	70	29	9	59	318

Table S64. Question 66 - Was it possible for immunity to wane?

	Malaria	Dengue	WNV	Filariasis	Other	Total
No	22% (38)	86% (60)	69% (20)	22% (2)	63% (37)	45% (144)
Not relevant	33% (58)	4% (3)	21% (6)	78% (7)	24% (14)	26% (82)
Yes, but waning was slowed down by re-exposure	18% (32)	0% (0)	0% (0)	0% (0)	2% (1)	10% (32)
Yes, in uninfected immune or partially immune hosts	26% (45)	6% (4)	7% (2)	0% (0)	10% (6)	17% (54)
Other	1% (2)	4% (3)	3% (1)	0% (0)	2% (1)	2% (6)
Total	175	70	29	9	59	318

Table S65. Question 67 - What clinical outcomes were described?

	Malaria		Dengue		WNV		Filariasis		Other		Total	
	Alone	Total	Alone	Total	Alone	Total	Alone	Total	Alone	Total	Alone	Total
Symptomatic and asymptomatic infections	16	20	2	4	2	2	0	0	1	2	20	26
Mild vs. severe symptoms	2	4	3	5	0	0	0	0	0	0	5	9
Disease induced mortality	13	17	3	4	14	15	1	1	8	10	37	44
Chronic sequelae	0	0	0	0	0	0	1	1	0	0	1	1
Low birth weights in children born to infected mothers	0	0	0	0	0	0	0	0	0	0	0	0
None of the above / Other	139	140	58	61	12	13	7	7	48	49	244	250

Table S66. Question 67 - What clinical outcomes were described?

Combination size	Malaria	Dengue	WNV	Filariasis	Other	Total
1	97% (170)	94% (66)	97% (28)	100% (9)	97% (57)	97% (307)
2	2% (4)	6% (4)	3% (1)	0% (0)	3% (2)	3% (10)
3	1% (1)	0% (0)	0% (0)	0% (0)	0% (0)	0% (1)
Total	175	70	29	9	59	318

Table S67. Question 68 - Was it possible for a vertebrate host to be superinfected or coinfected?

	Malaria	Dengue	WNV	Filariasis	Other	Total
No	70% (123)	91% (64)	100% (29)	33% (3)	92% (54)	80% (253)
Yes	30% (52)	9% (6)	0% (0)	67% (6)	8% (5)	20% (65)
Total	175	70	29	9	59	318

Table S68. Question 69 - How was superinfection or coinfection modeled?

	Malaria	Dengue	WNV	Filariasis	Other	Total
Infection with two or more types was represented with a state variable	32% (6)	100% (6)	– (0)	0% (0)	0% (0)	41% (12)
With a function describing waiting time to clear	5% (1)	0% (0)	– (0)	0% (0)	0% (0)	3% (1)
With an individual-based model	26% (5)	0% (0)	– (0)	0% (0)	25% (1)	21% (6)
Other	37% (7)	0% (0)	– (0)	100% (3)	75% (3)	34% (10)
Total	19	6	0	3	4	29

Table S69. Question 70 - What were the important differences among the vertebrate host species or types, included either by themselves (Alone) or together with other differences (Total)?

	Malaria		Dengue		WNV		Filariasis		Other		Total	
	Alone	Total	Alone	Total	Alone	Total	Alone	Total	Alone	Total	Alone	Total
Population density	0	9	0	2	0	3	0	0	0	5	0	14
Population dynamics	0	0	0	0	0	3	0	0	0	2	0	5
Movement patterns	0	1	0	1	0	1	0	0	0	1	0	2
Their attractiveness to mosquitoes	3	14	0	0	0	1	0	0	0	5	3	18
The intensity of infection and infectiousness to mosquitoes	1	9	0	2	0	4	0	0	0	5	1	15
The duration of the infectious period	0	6	0	2	1	7	0	0	0	4	1	15
The development of immunity	2	5	0	0	0	1	0	0	1	3	2	8
Some species were sinks for the pathogen	0	6	0	0	2	5	0	0	0	1	2	11
None of the above / Other	0	3	0	0	1	3	0	0	0	0	1	6

Table S70. Question 70 - What were the important differences among the vertebrate host species or types?

Combination size	Malaria	Dengue	WNV	Filariasis	Other	Total
1	27% (6)	0% (0)	33% (4)	– (0)	10% (1)	26% (10)
2	23% (5)	0% (0)	17% (2)	– (0)	40% (4)	26% (10)
3	36% (8)	50% (1)	33% (4)	– (0)	30% (3)	33% (13)
4	9% (2)	50% (1)	17% (2)	– (0)	20% (2)	13% (5)
5	5% (1)	0% (0)	0% (0)	– (0)	0% (0)	3% (1)
Total	22	2	12	0	10	39

Table S71. Question 71 - Did the paper call the different host variants species or different genotype / phenotype of the same species?

	Malaria	Dengue	WNV	Filariasis	Other	Total
Different species	50% (11)	100% (2)	92% (11)	– (0)	50% (5)	62% (24)
Different types of the same species	41% (9)	0% (0)	0% (0)	– (0)	20% (2)	26% (10)
The paper was deliberately vague	9% (2)	0% (0)	0% (0)	– (0)	10% (1)	5% (2)
Other	0% (0)	0% (0)	8% (1)	– (0)	20% (2)	8% (3)
Total	22	2	12	0	10	39

11. Mixing, Heterogeneous or Preferential Biting

Table S72. Question 72 - How were bloodmeals distributed among vertebrate hosts?

	Malaria	Dengue	WNV	Filariasis	Other	Total
Heterogeneously	13% (28)	3% (2)	7% (2)	18% (2)	14% (9)	11% (39)
Homogenously	80% (175)	91% (69)	87% (26)	36% (4)	70% (44)	82% (303)
Not relevant	7% (15)	7% (5)	7% (2)	45% (5)	16% (10)	7% (27)
Other	0% (0)	0% (0)	0% (0)	0% (0)	0% (0)	0% (0)
Total	218	76	30	11	63	369

TABLE S73. Question 73 - Which of the following were associated with heterogeneous biting?

	Malaria		Dengue		WNV		Filariasis		Other		Total	
	Alone	Total	Alone	Total	Alone	Total	Alone	Total	Alone	Total	Alone	Total
Host age	3	4	0	1	1	1	0	2	3	3	6	10
Host body size	5	7	0	0	0	0	0	0	0	0	5	7
Host behavior of any sort	1	1	0	0	0	0	0	0	0	0	1	1
Host infection status	4	5	0	0	0	0	0	0	0	1	4	5
Biting rates were described by a continuous probability distribution distribution	8	9	1	1	0	0	0	2	0	1	9	12
Biting rates were divided into a finite number of classes	1	1	0	1	0	0	0	0	0	0	1	2
None of the above / Other	3	4	0	0	1	1	0	0	4	4	7	8

Table S74. Question 73 - Which of the following were associated with heterogeneous biting?

Combination size	Malaria	Dengue	WNV	Filariasis	Other	Total
1	89% (25)	50% (1)	100% (2)	0% (0)	88% (7)	85% (33)
2	11% (3)	50% (1)	0% (0)	100% (2)	12% (1)	15% (6)
Total	28	2	2	2	8	39

Table S75. Question 74 - Which one of the following describes mixing?

	Malaria	Dengue	WNV	Filariasis	Other	Total
A specific contact network was specified or described	1% (2)	3% (2)	0% (0)	0% (0)	6% (4)	2% (8)
Mixing was not part of this model	11% (23)	0% (0)	6% (2)	31% (4)	9% (6)	8% (30)
Mixing was the outcome of an individual-based model	4% (9)	3% (2)	0% (0)	0% (0)	3% (2)	3% (13)
None of the above / Other	6% (14)	11% (8)	10% (3)	31% (4)	17% (11)	8% (31)
Well mixed	78% (171)	84% (64)	84% (26)	38% (5)	65% (42)	78% (291)
Total	219	76	31	13	65	373

Table S76. Question 75 - Which of the following parameters or terms describe transmission from the infectious mosquito to its vertebrate host?

	Malaria		Dengue		WNV		Filariasis		Other		Total	
	Alone	Total	Alone	Total	Alone	Total	Alone	Total	Alone	Total	Alone	Total
It was implicitly set to 1	40	41	16	16	0	0	2	2	10	11	65	67
It was represented by a constant parameter b	150	155	53	53	29	29	5	5	39	40	254	260
The parameter b differed in some way with partial immunity	6	7	0	0	0	0	0	3	0	0	6	10
The parameter b differed in some way with transmission intensity	0	0	0	0	0	0	0	1	1	1	1	2
The parameter b differed with the intensity of infection in mosquitoes	0	0	1	1	0	0	0	3	0	0	1	4
None of the above / Other	17	22	6	6	2	2	3	3	14	14	36	41

Table S77. Question 75 - Which of the following parameters or terms describe transmission from the infectious mosquito to its vertebrate host?

Combination size	Malaria	Dengue	WNV	Filariasis	Other	Total
1	97% (213)	100% (76)	100% (31)	77% (10)	98% (64)	97% (363)
2	3% (6)	0% (0)	0% (0)	15% (2)	2% (1)	2% (9)
3	0% (0)	0% (0)	0% (0)	8% (1)	0% (0)	0% (1)
Total	219	76	31	13	65	373

Table S78. Question 76 - Which of the following parameters or terms describes transmission from the infectious host to the mosquito?

	Malaria		Dengue		WNV		Filariasis		Other		Total	
	Alone	Total	Alone	Total	Alone	Total	Alone	Total	Alone	Total	Alone	Total
It was implicitly set to 1	38	40	19	19	1	1	2	3	12	13	67	69
It was represented by a constant c	132	138	47	48	28	28	3	3	34	35	229	236
It varied with the level of immunity in the vertebrate host	8	13	0	1	0	0	0	0	0	1	8	14
It varied with the age of the infection	2	2	0	0	0	0	0	1	0	0	2	3
It varied with pathogen loads	1	5	3	3	0	0	1	2	0	0	5	10
None of the above / Other	26	34	6	6	2	2	5	6	17	18	48	56

Table S79. Question 76 - Which of the following parameters or terms describe transmission from the infectious mosquito to its vertebrate host?

Combination size	Malaria	Dengue	WNV	Filariasis	Other	Total
1	95% (207)	99% (75)	100% (31)	85% (11)	97% (63)	96% (359)
2	5% (11)	1% (1)	0% (0)	15% (2)	3% (2)	3% (13)
3	0% (1)	0% (0)	0% (0)	0% (0)	0% (0)	0% (1)
Total	219	76	31	13	65	373

12. Control

Table S80. Question 77 - What types of control were considered, either by themselves (Alone) or together with other types of control (Total)?

	Malaria		Dengue		WNV		Filariasis		Other		Total	
	Alone	Total	Alone	Total	Alone	Total	Alone	Total	Alone	Total	Alone	Total
None	2	2	2	2	0	0	0	0	0	0	4	4
Introduction of genetically modified mosquito populations for population replacement (e.g. refractory mosquitoes)	1	1	0	0	0	0	0	0	0	0	1	1
Control of mosquito populations with sterile male release	0	0	0	0	0	0	0	0	0	0	0	0
Control of aquatic mosquito populations with larvicides	1	7	1	4	1	4	0	1	0	1	3	15
Biological control of mosquito populations	0	1	0	0	0	0	0	0	0	0	0	1
Introduction of genetically modified mosquito populations for population suppression	0	0	1	1	0	0	0	0	0	0	1	1
Control of adult mosquito populations with indoor spraying	2	11	0	1	0	1	0	1	0	1	2	13
Control of adult mosquito populations with mass spraying	2	6	4	7	3	7	0	2	0	2	9	22
Control of adult populations / transmission with bednets (insecticide treated or not)	11	27	0	0	0	0	0	1	1	4	11	28
Control of disease or transmission with a vaccine	16	22	7	8	0	0	0	0	6	7	26	34
Control of disease or transmission by treating clinical cases with drugs	23	35	0	0	0	0	0	1	0	0	23	36
Control of disease or transmission with mass screening and treating or mass drug administration	3	11	0	0	0	0	2	3	0	1	5	14
Control of transmission by culling the vertebrate host population	0	0	0	0	0	2	0	0	0	0	0	2
Control with zooprophylaxis	2	4	0	0	0	0	0	0	0	0	2	4
Other	15	20	0	2	0	5	1	2	3	3	15	28

Table S81. Question 77 - What types of control were considered?

Combination size	Malaria	Dengue	WNV	Filariasis	Other	Total
1	76% (78)	79% (15)	40% (4)	50% (3)	77% (10)	73% (102)
2	11% (11)	11% (2)	30% (3)	33% (2)	8% (1)	13% (18)
3	6% (6)	11% (2)	30% (3)	0% (0)	8% (1)	9% (12)
4	6% (6)	0% (0)	0% (0)	17% (1)	8% (1)	4% (6)
5	1% (1)	0% (0)	0% (0)	0% (0)	0% (0)	1% (1)
Total	102	19	10	6	13	139

Table S82. Question 78 - Which of the following aspects of control did the model consider, either by themselves (Alone) or together with other aspects of control (Total)?

	Malaria		Dengue		WNV		Filariasis		Other		Total	
	Alone	Total	Alone	Total	Alone	Total	Alone	Total	Alone	Total	Alone	Total
Costs	1	11	0	0	0	0	0	0	1	1	1	11
Operational constraints	0	0	0	0	0	0	2	2	0	0	2	2
Targeted application	0	6	0	1	0	0	0	0	0	0	0	7
Uneven or heterogeneous distribution	3	10	1	3	0	0	0	1	2	4	4	15
How well the intervention works	54	72	13	17	8	8	3	4	6	9	79	103
Other	23	30	1	3	2	2	0	0	1	2	26	35

Table S83. Question 78 - Which of the following aspects of control did the model consider?

Combination size	Malaria	Dengue	WNV	Filariasis	Other	Total
1	79% (81)	79% (15)	100% (10)	83% (5)	77% (10)	81% (112)
2	17% (17)	16% (3)	0% (0)	17% (1)	23% (3)	16% (22)
3	2% (2)	5% (1)	0% (0)	0% (0)	0% (0)	2% (3)
4	2% (2)	0% (0)	0% (0)	0% (0)	0% (0)	1% (2)
Total	102	19	10	6	13	139

13. Description of the Analysis

Table S84. Question 79 - What aspects of an epidemic were analyzed, either by themselves (Alone) or together with other aspects of an epidemic (Total)?

	Malaria		Dengue		WNV		Filariasis		Other		Total	
	Alone	Total	Alone	Total	Alone	Total	Alone	Total	Alone	Total	Alone	Total
Thresholds	26	115	3	44	7	24	0	7	8	34	41	212
The evolution of an epidemic	4	36	5	32	0	10	0	1	0	21	9	95
Stochastic invasion of a pathogen	0	4	0	5	0	0	0	3	0	11	0	18
Controlling an outbreak	0	18	0	8	1	7	0	0	0	2	1	35
Modeling control effect sizes or responses	6	38	0	11	0	6	1	3	0	3	7	60
Seasonality and its consequences	1	23	1	17	0	3	0	0	0	11	2	52
Population cycles	0	7	0	10	0	2	0	0	0	5	0	22
Steady states / endemicity	16	102	4	34	0	16	0	7	2	22	21	171
Local persistence of the pathogen	0	15	0	5	0	4	0	3	0	6	0	33
Stochastic fadeout	0	2	0	1	0	0	0	0	0	2	0	5
Low intensity or declining transmission	0	11	0	1	0	0	0	1	0	1	0	14
Control response timelines	1	25	0	3	0	1	0	3	0	0	1	32
Spatial persistence	0	5	0	2	0	1	0	0	0	2	0	10
Mapping potential transmission	4	12	0	5	0	0	0	0	1	3	5	19
Pathogen evolution	0	24	0	3	0	0	0	0	0	3	0	28
Host evolution	0	0	0	0	0	0	0	0	0	0	0	0
Mosquito evolution	0	4	0	0	0	0	0	0	0	1	0	4
Analysis of a time series	1	15	0	6	0	0	0	0	0	1	1	22
Economics or bioeconomics	0	6	0	0	0	0	0	0	0	1	0	6
Mosquito behavior	5	8	0	2	0	0	0	0	1	1	5	10
Vertebrate host behavior	0	0	0	0	0	0	0	0	0	0	0	0
None of the above / Other	13	42	5	9	2	4	4	4	8	12	21	60

Table S85. Question 79 - What aspects of an epidemic were analyzed?

Combination size	Malaria	Dengue	WNV	Filariasis	Other	Total
1	35% (77)	24% (18)	32% (10)	38% (5)	31% (20)	31% (114)
2	29% (64)	29% (22)	29% (9)	8% (1)	37% (24)	30% (112)
3	20% (44)	26% (20)	10% (3)	31% (4)	23% (15)	21% (80)
4	7% (16)	12% (9)	16% (5)	15% (2)	5% (3)	9% (34)
5	4% (9)	4% (3)	10% (3)	8% (1)	2% (1)	5% (17)
6	1% (2)	4% (3)	3% (1)	0% (0)	3% (2)	2% (8)
7	1% (3)	1% (1)	0% (0)	0% (0)	0% (0)	1% (4)
8	1% (3)	0% (0)	0% (0)	0% (0)	0% (0)	1% (3)
9	0% (1)	0% (0)	0% (0)	0% (0)	0% (0)	0% (1)
Total	219	76	31	13	65	373
